# Lattice-Nitrogen-Mediated Chemistry Suppresses Hydrogen Evolution for Record Faradaic Efficiency in Ammonia Synthesis

**DOI:** 10.1021/jacs.5c09104

**Published:** 2025-07-31

**Authors:** David Kumar Yesudoss, Hao-En Lai, Denis Johnson, Mark Lee, Benjamin Reinhart, Perla B. Balbuena, Abdoulaye Djire

**Affiliations:** † Artie McFerrin Department of Chemical Engineering, 14736 Texas A&M University, College Station, Texas 77843, United States; ‡ Wm Michael Barnes Department of Industrial and Systems Engineering, Texas A&M University, College Station, Texas 77843, United States; § X-ray Science Division, 1291 Argonne National Laboratory, Argonne, Lemont, Illinois 60439, United States; ∥ Department of Chemistry, Texas A&M University, College Station, Texas 77843, United States; ⊥ Department of Materials Science and Engineering, Texas A&M University, College Station, Texas 77843, United States

## Abstract

Ammonia (NH_3_) production using air, water, and electricity offers a transformative route to carbon-free chemical synthesis, addressing global sustainability challenges. However, the hydrogen evolution reaction (HER) in aqueous systems significantly hinders NH_3_ selectivity, limiting the Faradaic efficiency (FE) to below ∼15%. Herein, we report an FE of approximately 48% for aqueous NH_3_ synthesis, using two-dimensional (2D) nitride catalysts. These catalysts enable lattice nitrogen protonation through the Mars-van Krevelen (MvK) mechanism, effectively suppressing HER. Using operando spectroelectrochemistry, we identified active sites and tracked nitrogen vacancy cycles, providing unprecedented insights into the reaction pathways. Our findings, supported by advanced computational techniques and complementary spectroscopic analyses, highlight the stability and efficiency of the MvK cycle, setting a new benchmark for sustainable NH_3_ production.

## Graphical abstract

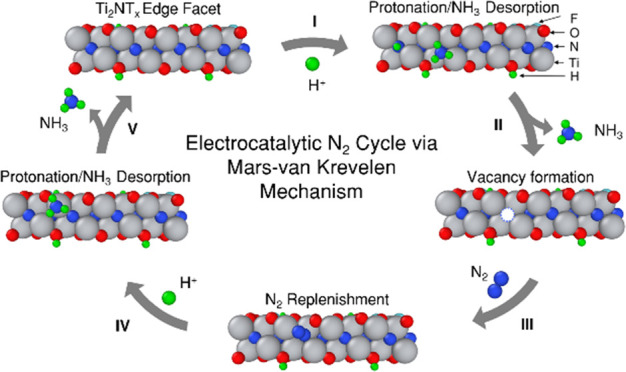

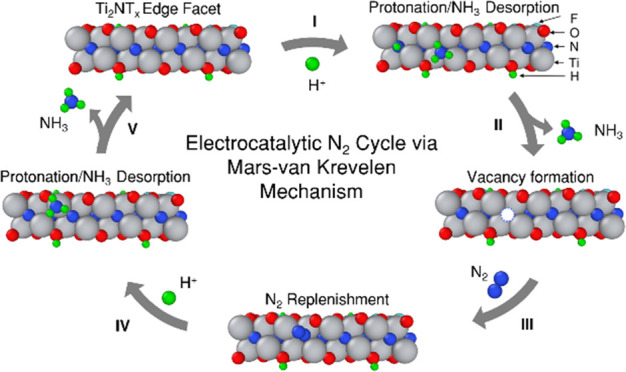

## Introduction

Ammonia (NH_3_) is a cornerstone of modern industry, enabling global food security and economic growth.^1–3^ However, its large-scale production via the Haber–Bosch process is energy-intensive, heavily reliant on fossil fuels, and inaccessible in some regions due to infrastructure limitations. Electrochemical NH_3_ synthesis through the nitrogen reduction reaction (NRR) offers a transformative alternative, capable of operating under ambient conditions and achieving carbon neutrality when powered by renewable energy.^4–6^ This approach also opens the door to decentralized NH_3_ production, addressing global inequities in access to this critical resource.^7–9^

Despite its promise, the NRR efficiency is hindered by the competing hydrogen evolution reaction (HER) and the high activation energy required to break the N≡N triple bond. Traditional NRR mechanisms, whether associative or dissociative, struggle with low energy efficiency.^10–14^ Recently, single-atom catalysts such as Fe_SA_–N–C^15^ and Rh_1_/MnO_2_ ^16^ have demonstrated promising FE of up to 56.55 and 73.3%, respectively. However, atomically dispersing metals in a stable and uniform manner often involves complex synthesis procedures, which can be difficult to scale or reproduce. The Mars-van Krevelen (MvK) mechanism offers an innovative pathway, leveraging lattice nitrogen atoms as intermediates.^17–20^ Protonation of the lattice N leads to the formation of N vacancies following the NH_3_ desorption, which are then replenished via the N_2_ feed gas. For instance, thermocatalytic studies on Co_3_Mo_3_N have demonstrated that lattice nitrogen can be reversibly exchanged during ammonia synthesis, with direct experimental evidence from isotopic labeling confirming the MvK pathway.^21,22^ Recent theoretical studies have also shown that the MvK pathway is kinetically more favorable than the conventional Langmuir–Hinshelwood type pathway on several metal nitrides, particularly those with stable lattice N vacancy and moderate vacancy formation energies.^20^ Microkinetic modeling on rock salt-type VN(100), for example, has revealed that the rate-determining step for MvK is significantly more accessible than N_2_ dissociation in associative routes and that lattice hydrogenation proceeds more readily at lattice N sites than metal sites. However, realizing this mechanism requires catalysts with stable nitrogen vacancies and high electronic conductivity.^23,24^ Transition-metal nitrides, including MNenes, have emerged as strong candidates due to their tunable stoichiometry, electronic properties, and structural stability.^25–30^ However, existing bulk transition-metal nitrides, like VN and TiN, degrade over the course of NRR because of the instability of N vacancies.^31,32^

Prior theoretical investigations on the MvK mechanism have largely focused on surface functional groups and axial reaction mechanisms,^33–36^ with limited exploration of its edges, apart from one study on porous MNenes.^37^ Similarly, studies on related MXene materials such as Ti_3_C_2_T_*x*_ ^38,39^ and Ti_2_CO_2_ ^40^ have not adequately addressed edge phenomena. To fully understand the existence and evolution of nitrogen vacancies at MNene edges for NRR, an integrated computational and experimental approach is urgently needed.

In this work, we investigate the Ti_2_NT_*x*_ MNene as a catalyst for NRR in aqueous environments, achieving an FE of 47.5 ± 2.8%. Through integrated experimental and computational approaches, including isotopic labeling, operando X-ray absorption spectroscopy (XAS), density functional theory (DFT), and ab initio molecular dynamics (AIMD), we demonstrate that the Ti_2_NT_*x*_ catalyst operates via a stable and sustained MvK mechanism. Notably, we identify edge sites as the primary loci of nitrogen vacancy formation and regeneration, supported by experimental results and theoretical calculations. These findings represent a significant step toward scalable, sustainable NH_3_ synthesis and advance the fundamental understanding of MNene catalysis. A schematic of our proof-of-concept of the MvK mechanism compared with the conventional associative and dissociative pathways is shown in Scheme 1.

**Scheme 1:**
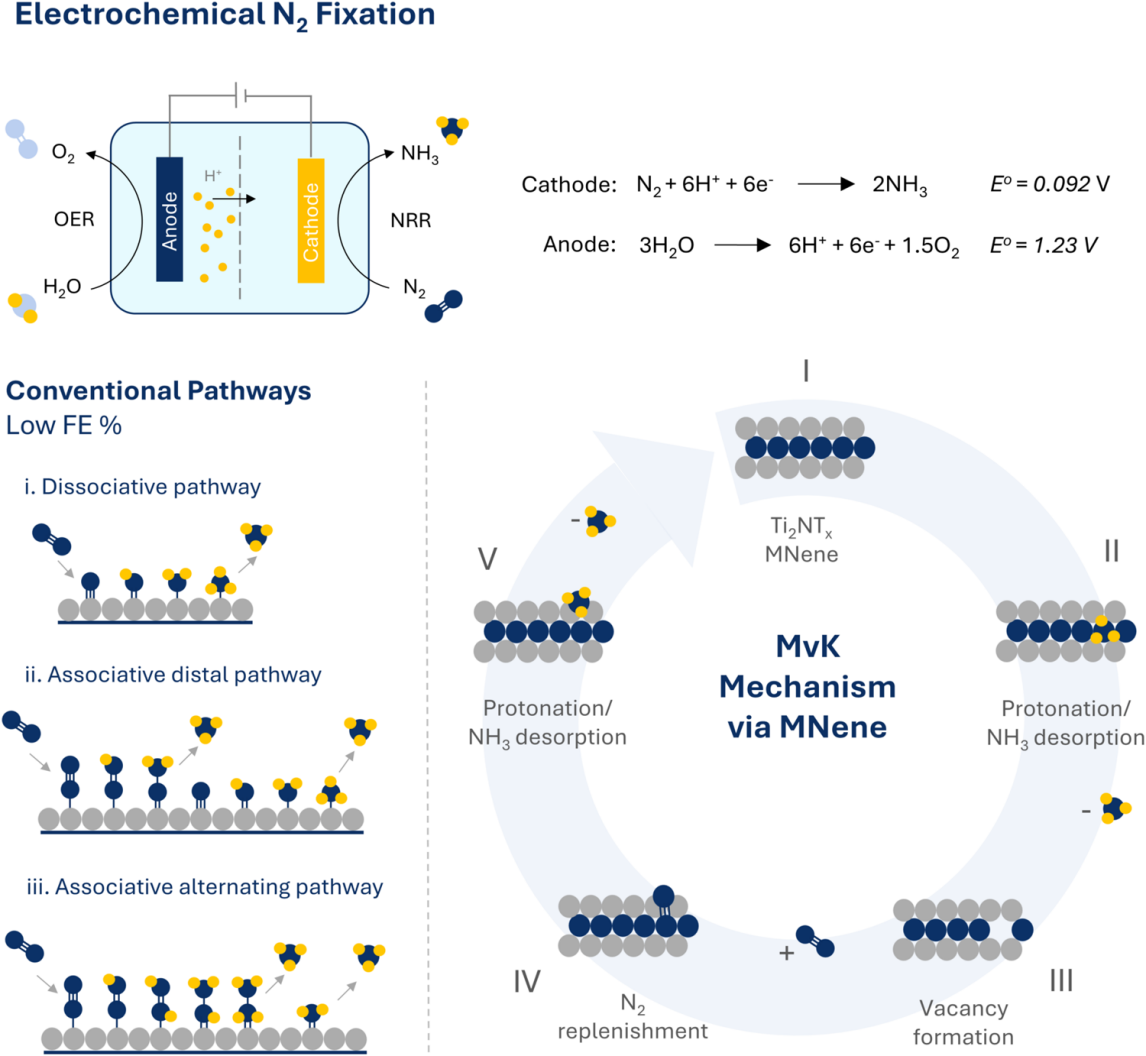
Illustration of conventional electrochemical surface N_2_ adsorption/activation mechanisms vs. unique MvK pathway exhibited by Ti_2_NT*_*x*_* MNene.^a^

a The MvK mechanism enables high NH_3_ FE in aqueous environments.

## Results and Discussion

### Synthesis and Characterization of Ti_2_NT_*x*_ MNene

The Ti_2_NT_*x*_ MNene catalyst was synthesized through a modified etching process adapted from our prior work (Figure 1a).^17^ Characterization by X-ray diffraction (XRD) and Raman spectroscopy confirmed phase purity and structural integrity, consistent with previous reports (Figure 1b,c).^17^ In the XRD patterns (Figure 1b), the characteristic (002) peak shifted from 12.9° in the Ti_2_AlN precursor to 9.6° in the Ti_2_NT_*x*_ MNene, corresponding to an increase in interlayer spacing from 0.68 to 0.92 nm. This expansion indicates the removal of the Al layer in Ti_2_AlN and the formation of well-exfoliated Ti_2_NT_*x*_ sheets. Notably, no diffraction peaks associated with residual fluoride salts (LiF, KF, and NaF) were observed, indicating the effectiveness of the acid washing step and the high phase purity of the final material. Raman spectroscopy (Figure 1c) further supported the Al etching, showing the disappearance of the Ti_2_AlN precursor’s characteristic vibrational modes and the emergence of a distinct ∼400 cm^–1^ band associated with Ti atomic plane vibrations in Ti_2_NT_*x*_. Additionally, a shift and broadening of the A_1g_ mode was observed, consistent with lattice distortions and modifications induced by surface terminations during the etching process. To elucidate the chemical state and atomic structure, ex situ XAS was performed at the Ti K-edge. X-ray absorption near-edge structure (XANES) and Fourier-transformed extended X-ray absorption fine structure (FT-EXAFS) analyses revealed changes in Ti valence states and bond environments during synthesis (Figure 1d,e). Using a calibration curve derived from first-derivative spectra of Ti reference materials (Figure S1, Supporting Information), we determined Ti valence states of 2.1 for the Ti_2_AlN MAX phase, 3.6 for the multilayer (ML) MNene, and 3.9 for the delaminated (DL) MNene. These changes correspond to the replacement of Ti–Al metallic bonds in the MAX phase with Ti–N and Ti–O bonds in MNene, which is induced by etching and surface functionalization. EXAFS analysis (Figure S2) identified peaks at 1.5 Å (Ti–N/Ti-O), 2.5 Å (Ti–Ti), and 3.7 Å (Ti–N–Ti multiple scattering), consistent with literature values.^41–43^ These features were preserved across all synthesis stages (Figure 1e), indicating that the conversion from MAX to MNene retains the necessary local bonding motifs essential for catalytic performance.

**Figure 1:**
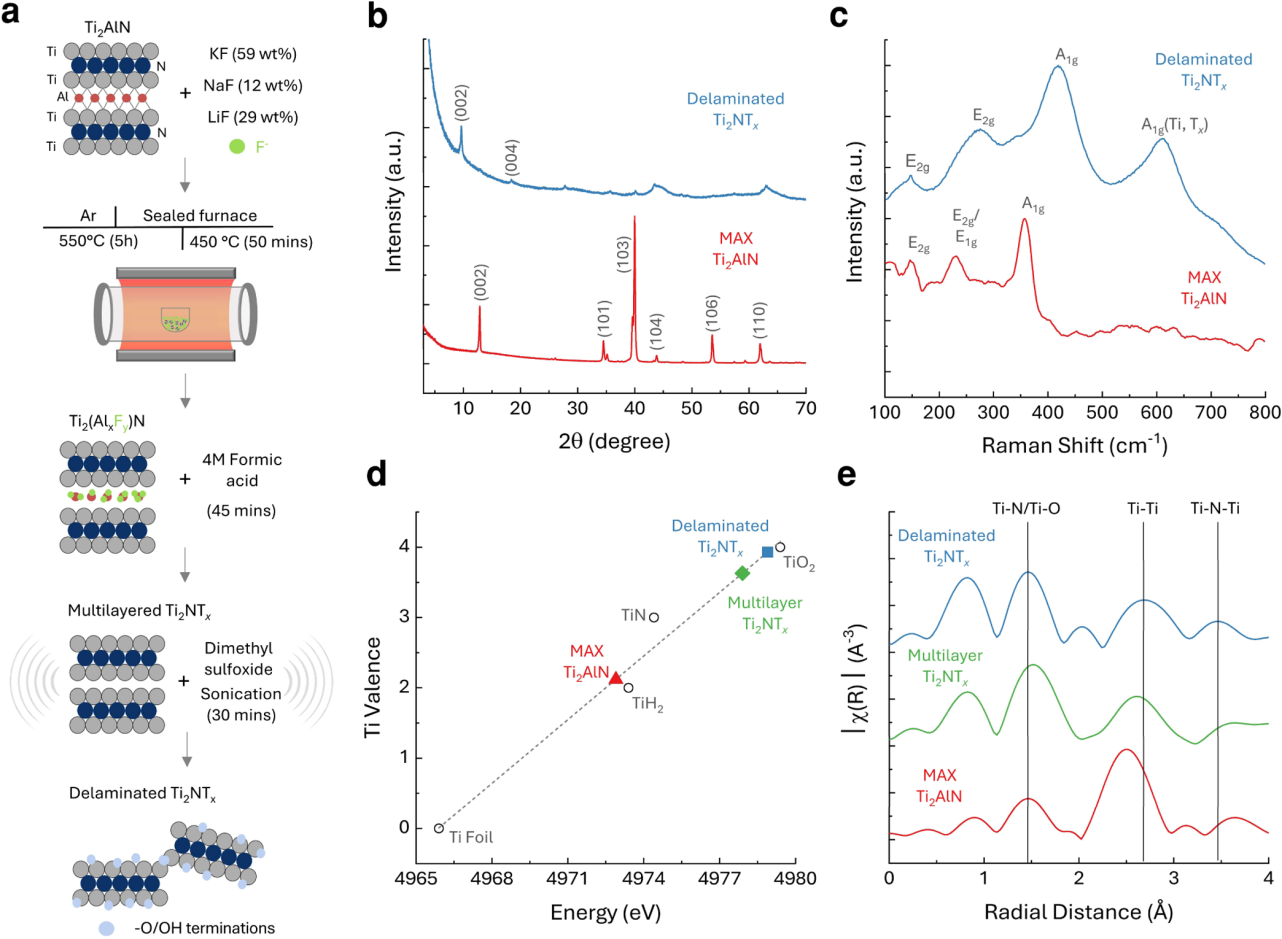
(a). Schematic of Ti_2_NT_*x*_ MNene synthesis via oxygen-assisted molten salt fluoride treatment of the Ti_2_AlN MAX phase (detailed procedure in Experimental Section). (b). X-ray diffraction patterns of Ti_2_AlN MAX (red) and delaminated Ti_2_NT_*x*_ MNene (blue) were measured on a zero-diffraction silicon holder. (c). Raman spectra of Ti_2_AlN MAX (red) and delaminated Ti_2_NT_*x*_ MNene (blue) were collected with a 532 nm laser. (d). Ti valence as a function of edge energy from XANES spectra for reference compounds (hollow circles), Ti_2_AlN MAX (red), multilayer Ti_2_NT_*x*_ (green diamond), and delaminated Ti_2_NT_*x*_ MNene (blue square). (e). Fourier transform of normalized EXAFS spectra for Ti_2_AlN MAX (red), multilayer Ti_2_NT_*x*_ (green), and delaminated Ti_2_NT_*x*_ MNene (blue), using a k weight of 2 for EXAFS fitting.

### NRR Performance of Ti_2_NT_*x*_ MNene Catalyst

The Ti_2_NT_*x*_ MNene catalyst demonstrated an excellent NRR performance in neutral electrolytes (LiCl, NaCl, and Na_2_SO_4_, 0.1 M). Cyclic voltammetry (CV) and linear sweep voltammetry (LSV) measurements identified an NRR onset potential near 0 V (vs RHE) and an HER onset at approximately −0.8 V, providing a favorable electrochemical window for NRR, especially compared with acidic systems (Figures 2a, S3 and S4, Supporting Information). The FE increases with decreasing cathodic potential in Na_2_SO_4_ and achieved an FE of 47.5 ± 2.82% for NH_3_ production, with comparable performance observed in LiCl and NaCl electrolytes (Figures 2a,b, S13 and S14). Post-experiment XAS revealed a correlation between the Ti oxidation state and catalytic performance. For example, an increase in Ti oxidation state from ∼3.6 at 0 V to ∼4.0 at −0.2 V could be responsible for the decline in FE from 47.5 to 12.2% (Figures 2c, S17). Chronoamperometry and CV measurements further supported this observation, showing that the sample operated at lower cathodic potentials retained higher current density, whereas performance degradation is observed at higher cathodic potentials (Figures 2d, S18). This observation is likely due to the MvK mechanism, as lattice nitrogen is extracted to form NH_3_ under NRR conditions, and nitrogen vacancies are created within the Ti_2_NT_*x*_ lattice. In the absence of complete nitrogen replenishment from the N_2_ gas phase at higher cathodic potentials, these vacancies become susceptible to the incorporation of oxygen from the electrolyte, leading to localized surface oxidation. These results underscore the importance of potential-dependent control over vacancy dynamics and the conditions under which the MvK cycle remains reversible.

**Figure 2:**
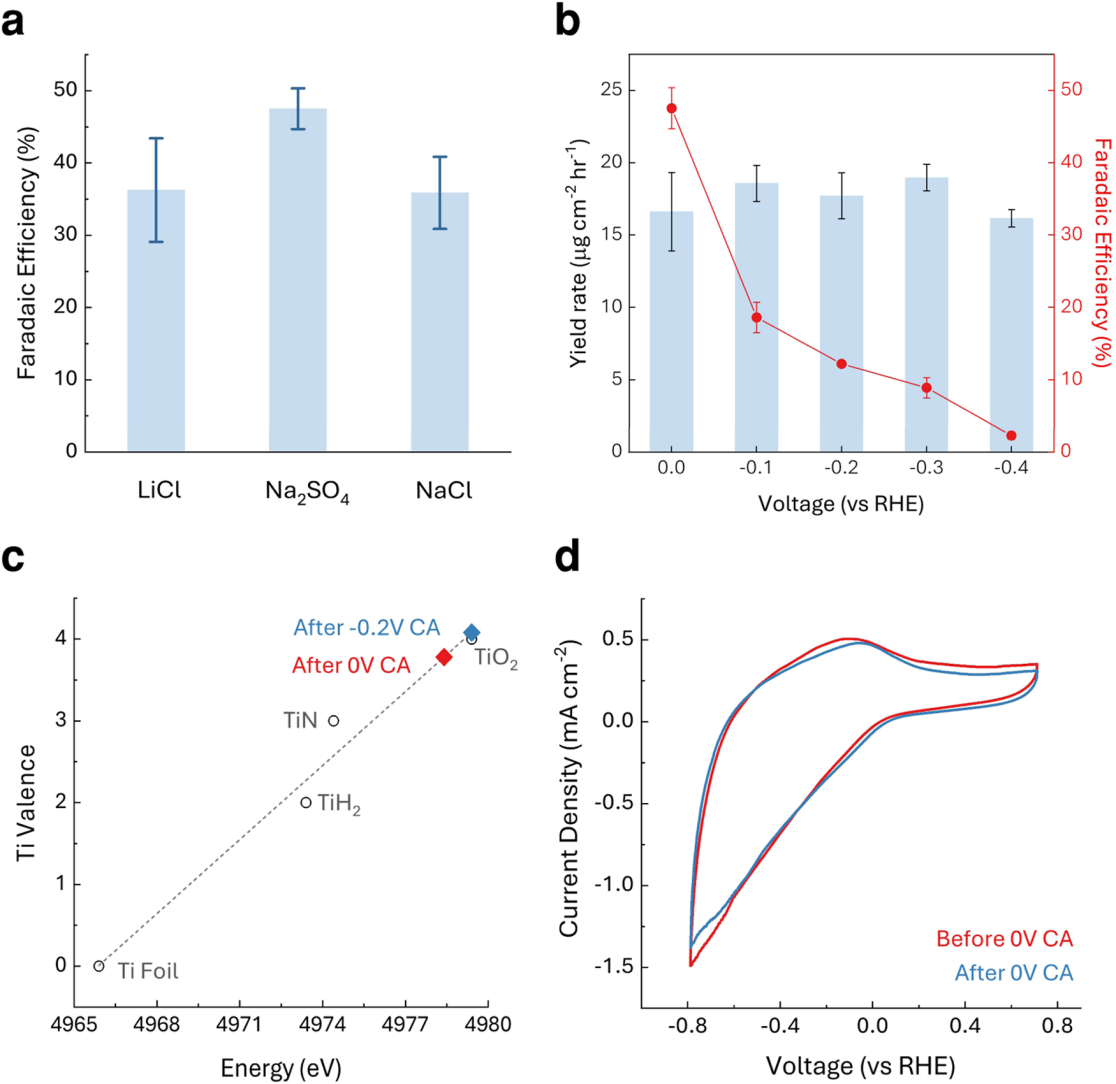
(a). Faradaic efficiencies for NH_3_ synthesis were obtained across various neutral electrolytes. (b). NH_3_ Faradaic efficiency and yield rate of Ti_2_NT*_*x*_* MNene in N_2_-saturated 0.1 M Na_2_SO_4_ electrolyte at different potentials after 4 h of chronoamperometry. (c). XANES-derived edge positions of the MNene catalyst after 4 h of chronoamperometry at 0 V vs RHE (red diamond) and −0.2 V vs RHE (blue diamond), indicating Ti valence changes. (d). Cyclic voltammograms before (red) and after (blue) 4 h chronoamperometry at 0 V vs RHE, measured at a scan rate of 50 mV s^–1^.

Recent studies have highlighted that ubiquitous nitrogen-containing species, such as NH_4_^+^ and NO_*x*_, can reach levels up to ∼40 nmol, even when using ultra high purity gas supplies.^44^ Additionally, ammonia contamination from ambient air and the prolonged sitting of the electrolyte can introduce further complications, potentially leading to overestimation of NRR performance, a challenge particularly critical for catalysts with inherently low ammonia yield rates. While control experiments, such as isotope labeling with ^15^N_2_, are typically used to resolve such ambiguities, they become difficult to apply in systems governed by the MvK mechanism, where the catalyst inherently contains lattice ^14^N. As a result, one effective strategy to reduce the influence of ammonia contamination is to move away from low yield regimes, that is, to increase the total amount of electrochemically produced ammonia so that background contamination becomes negligible relative to the signal. To address this, we strategically employed a Ti_2_NT_*x*_ catalyst spray-coated onto a gas diffusion layer (GDL) electrode with a geometric surface area of 4 cm^2^ for the NRR experiments (Figure S15a). This GDL electrode includes a lid on the backside through which N_2_ gas was purged at 2 mL min^–1^, ensuring direct and continuous access of N_2_ to the catalyst surface via microporous channels, effectively overcoming N_2_ solubility limitations. Incorporating this setup, the observed total NH_3_ produced increased significantly compared with the experiments carried out using a low geometric area (0.192 cm^2^) glassy carbon electrode. Therefore, the observed NH_3_ production is primarily from electrochemical NRR instead of contamination. Overall, the use of the GDL configuration provides greater confidence in the NRR results, as the total ammonia production is well above the threshold of interference from ambient ammonia contamination.

### Determining the Mars-van Krevelen Mechanism

The observed high FE of the MNene garners interest in the NH_3_ formation mechanism. To study this, the feed gas was changed to analyze the production rate and product identity (Figures 3, S19–S21). First, Ar rather than N_2_ was used to achieve an inert atmosphere within the reaction vessel for 68 h. If N from the catalyst was the N source, then there would be considerable NH_3_ production. In Figure S19a, during the first four hours of catalytic conditions, ∼6.1 μg of NH_3_ was produced, corresponding to 70% of the total lattice N being converted to NH_3_. Over 68 h of catalytic conditions, the amount of produced NH_3_ stays below the maximum theoretical in the case of 100% lattice N conversion. The slight NH_3_ decrease produced after 50 h is attributed to membrane crossover.^45^ Furthermore, the NH_3_ yield rate of the catalyst starts at ∼7.8 μg cm^–2^ h^–1^ before exponentially decaying toward zero with time, due to bulk N being protonated and desorbing. The same trend exists for FE due to applied charge going toward stabilizing the vacancy-filled MNene structure (Figure S19b, Supporting Information). The source of the N for NH_3_ production was further corroborated through N_2_ isotopic exchange experiments repeated on the same electrode in a semibatch mode (Figure 3). It can be observed that upon first introduction of ^15^N_2_ into the system, a mixture of ^14^NH_4_^+^ and ^15^NH_4_^+^ is observed in the 1H-NMR spectrum. After the first introduction of ^15^N_2_, the electrode was removed from the system and the cell thoroughly cleaned before fresh electrolyte was filled and the cell reassembled. This process was repeated 3 times, and it was observed that the ratio of ^15^NH_4_^+^ to ^14^NH_4_^+^ increases over these trials indicating that bulk nitrogen atoms are being involved in the reaction sequence and getting replaced with the ^15^N atoms. This is further shown when switching back to ^14^N_2_ as the feed, where the ratio of ^15^NH_4_^+^ to ^14^NH_4_^+^ can be seen to rapidly decrease before only ^14^NH_4_^+^ is observed in the NMR spectrum. This sequence is only possible if the bulk nitrogen atoms in the structure are involved in the reaction, leading to mixtures of isotopes. This further explains the stability of nitrogen vacancies as ^15^N is filled in the structure.

**Figure 3:**
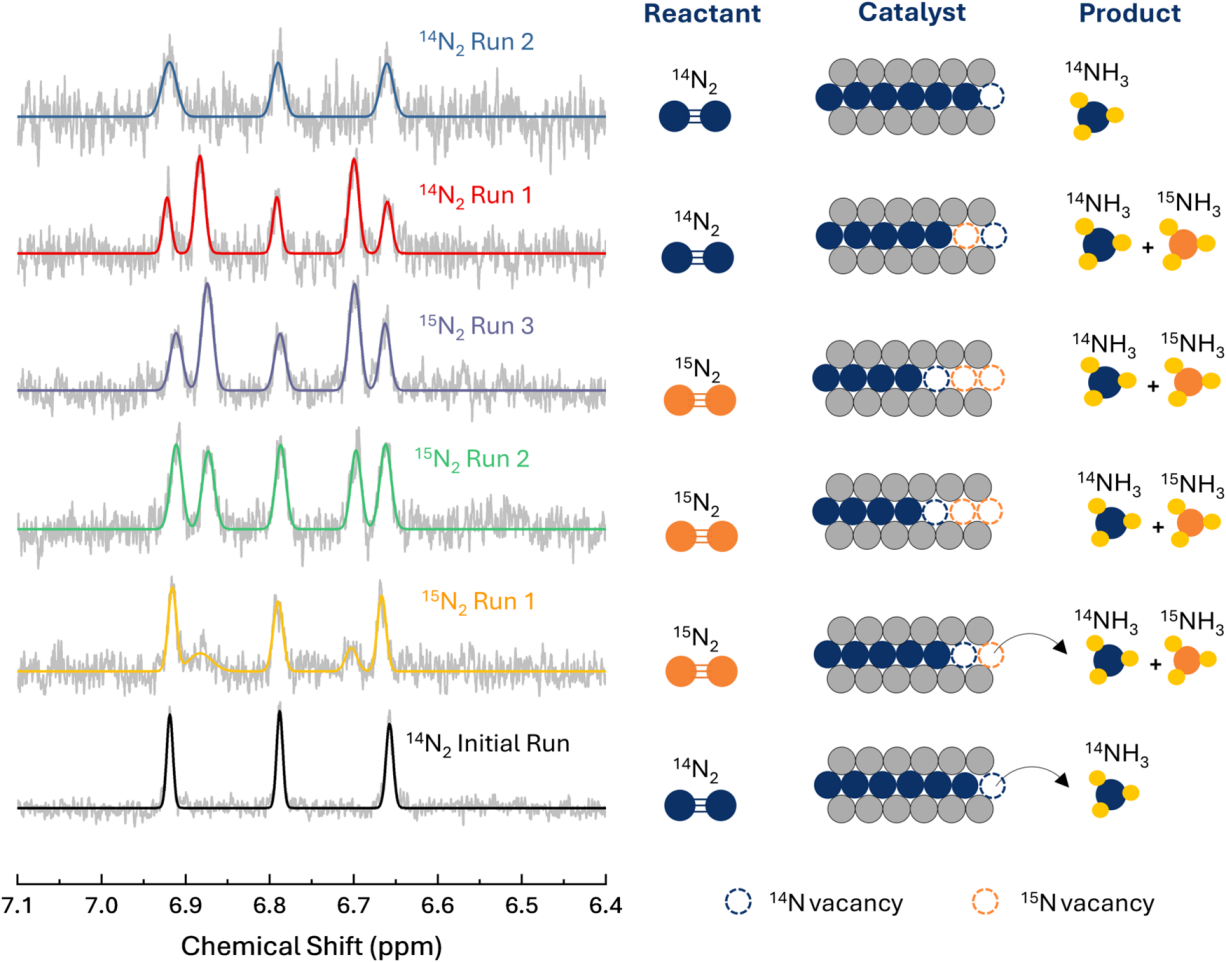
1H-NMR results from isotopic exchange experiments for the NH_3_ detection. Detected products from each experimental run are indicated to the right of their respective spectrum. Results demonstrate the existence of the MvK mechanism in MNenes.

### Investigation of Mars-van Krevelen Mechanism Pathway through DFT Free Energy Profiles

We explore three possible MvK mechanisms via DFT: the associative Heyrovsky MvK, MvK_as_, (hydrogenations occur on the N-site), the dissociative Heyrovsky MvK, MvK_dis_, (N_2_ dissociates on the N-site, then undergoes associative steps on the Ti bridge site), and a new associative-dissociative Heyrovsky MvK, MvK_as‑dis_, (dissociation occurs after NH_2_ formation, followed by migration to the Ti bridge site), further defined in the methods section in SI. The surfaces under investigation include Ti_2_N­(OH)­O, Ti_2_N­(OH)_2,_ Ti_2_NO_2_, and Ti_2_NO_2_ with edge −OH terminations. These mechanisms are studied under both neutral and acidic electrolyte conditions to provide insights into their MvK behavior. The newly proposed MvK_as‑dis_ mechanism is additionally investigated (Figures S22 and S23, Supporting Information). We include all three possible MvK mechanisms in our energy profile calculations (Table S1, Supporting Information). Detailed discussion of different MvK mechanisms on various Ti_2_NT_*x*_ MNene edge surfaces (Figures S24–S27, Supporting Information) is elaborated in the Supporting Information. MvK_as_ is found to be the most favorable mechanism across all surfaces, with MvK_as‑dis_ potentially occurring concurrently. Ti_2_N­(OH)O has the highest energy barrier for nitrogen vacancy formation, while Ti_2_NO_2_ requires the least energy. Edge −OH terminations on Ti_2_NO_2_ increase the energy barrier for vacancy formation but facilitate the spontaneous protonation of replenished vacancies. Controlling pH can modify edge surface protonation, potentially promoting the MvK mechanism. However, a trade-off exists between nitrogen vacancy formation and hydrogenation of replenished vacancies, influenced by the ratio of −OH edge terminations on the Ti_2_NO_2_ surface.

### Timing and Extent of Lattice Nitrogen Protonation

AIMD simulations suggest that nearby hydronium ions in neutral and acidic solutions play a crucial role in the N replenishment step. Specifically, hydrogenation by nearby hydronium ions impedes complete embedding of N_2_ molecules, leading to partial embedding that favors the MvK mechanism (Figures 4a–f, S28–S31, Supporting Information). By “partially embedded,” we refer to one nitrogen atom from nitrogen gas filling into a single nitrogen vacancy site; and “fully embedded” refers to two nitrogen atoms from nitrogen gas filling into a single nitrogen vacancy site. This occurs primarily due to the steric hindrance from N_2_H or N_2_H_2_ molecules and the reduced electronegativity of the N atom after protonation. This distinctive embedding behavior is closely related to whether the N–N bond breaking can happen spontaneously. When full embedding occurs (i.e., both nitrogen atoms fill the single vacancy) (Figures 4g–l, S33 and S34, Supporting Information), N–N bond breaking does not occur due to strong Ti–N coordination. Alternatively, in cases of partial embedding, N–N bond breaking is observed (Figures 4c,d, S28–S31, Supporting Information), facilitated by reduced steric hindrance that allows the formation of N_2_H or N_2_H_2_ at the vacancy sites, weakening the N–N bond. We note that prompt hydrogenation from the hydronium ions prevents all of the N_2_ gas molecules from entering the N vacancy on the edge sites. Additionally, multiple Ti–N coordination prevents the N atom from leaving the surface, hindering multiple hydrogenations on a single nitrogen, resulting in mostly embedded NHNH as well as if N_2_ is close to nearby vacancies and bridge sites before filling into vacancy. Therefore, if two or more protonation steps yield NNH_2_ and NNH_3_, the adsorbent is likely to move to nearby bridge sites instead of filling into vacancy sites (Figures S34–S37, Supporting Information). Without the replenishment step, the energy barrier for N_2_ bond breaking becomes challenging, and no N–N bond breaking is observed in the bridge NRR pathway during our AIMD simulations.

**Figure 4:**
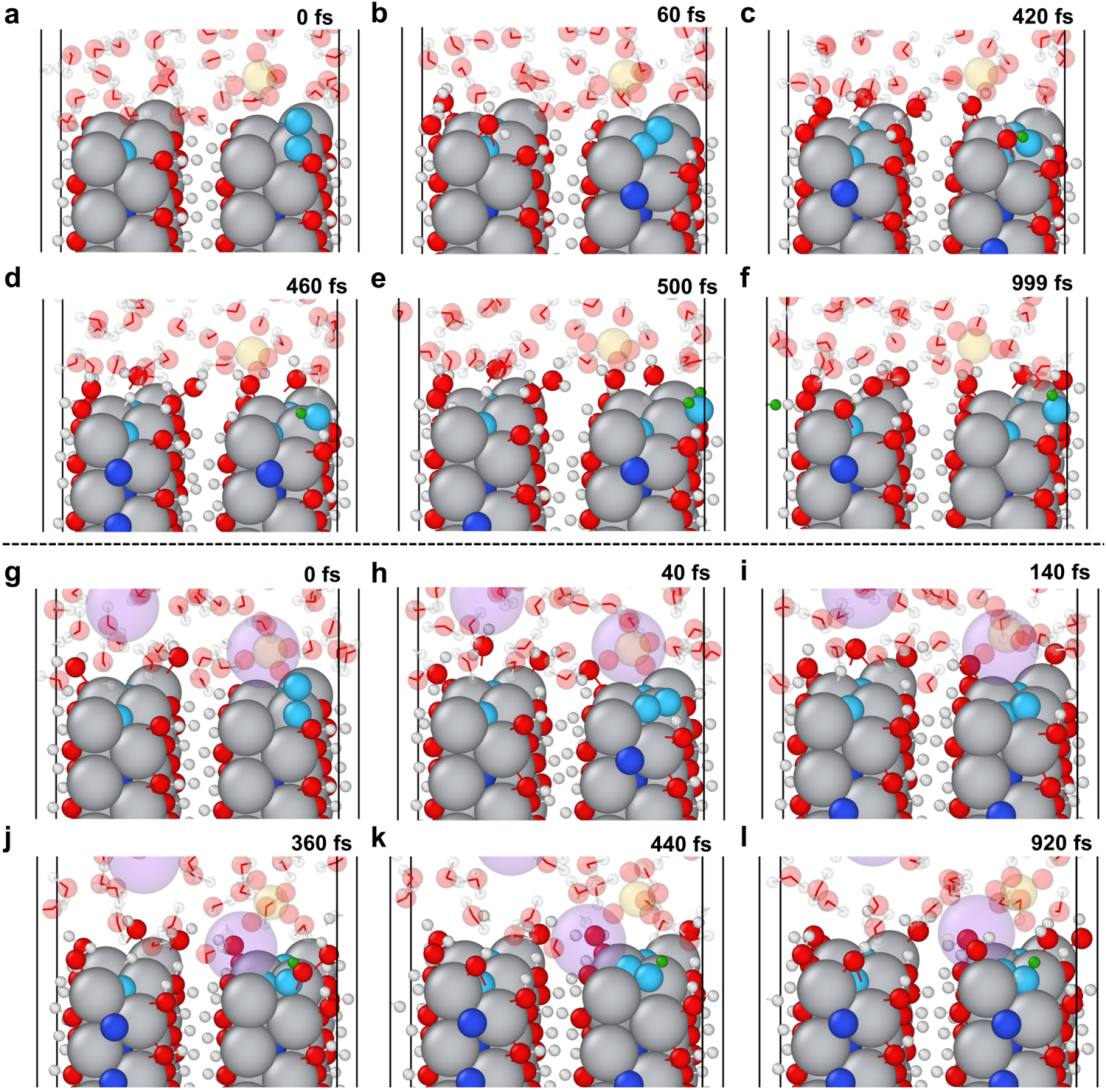
Selected snapshots from 1 ps AIMD simulations of adsorbed N_2_ near a nitrogen vacancy N_vac_ on the Ti_2_N­(OH)_2_ bilayer model (θ = 1/4). The vacancy filling process may vary from partial to full under different hydronium concentrations, potentially influencing the feasibility of the MvK mechanism. **a–f.** N_2_ partially embeds in *_vac_ and follows the MvK_as‑dis_ pathway on the surface in 1.25 M H_2_SO_4_. (a). N_2_ was positioned near *_vac_. (b). N_2_ partially filled *_vac_. (c). After the first protonation, the N–N bond begins breaking. (d). The N–N bond breaks. (e). Protonation of NH leads to NH_2_ formation. (f). NH_2_ remains on the Ti_top_ near the interlayer. **g–l** N_2_ fully embeds in *_vac_ without undergoing NRR in a 1.25 M Na_2_SO_4_ solution. (g). N_2_ was positioned near *_vac_. (h). N_2_ begins filling the nitrogen vacancy. (i). Without prompt protonation, N_2_ fully occupies the nitrogen vacancy. (j). After the first protonation, the NNH formed. (k). The embedded N atom moves upward, and N_2_ becomes horizontally embedded and gets trapped in the vacancy site. (l). NNH remains fully embedded, with the N–N bond intact. Cyan, blue, white, green, gray, red, and yellow represent nitrogen atoms above the first layer, nitrogen atoms below the first layer, hydrogen, hydrogen adsorbed on nitrogen, titanium, oxygen, and sulfur, respectively.

### Importance of −OH Termination on Edge Surface and Proton Concentration of H Source

Analyzing the −OH/O ratio on the Ti_2_NT*_*x*_* surface is crucial for understanding the MvK mechanisms. −OH termination influences water splitting equilibrium in neutral electrolyte, hydronium concentration in acidic electrolyte, and the embedding and breaking of N_2_. AIMD simulations of nitrogen vacancy replenishment demonstrate the Ti_2_N­(OH)_2_ surface undergoing an associative-dissociative MvK mechanism shown in Figure 4b–d. From an overall comparison, both OH_edge_O and O_edge_OH terminations promote N_2_ embedding, while O_edge_O terminations discourage it (Figures 5a–h, S38 and S39, Supporting Information). Interestingly, the accessibility of −OH terminations near the edge surface influences the MvK via hydrogen bonding. Among different surfaces, Ti_2_N­(OH)O (OHO_edge_O) exhibits more frequent and stronger interlayer −OH bonds, stabilizing the interlayer, but making OH bonds more difficult to break.^36^ Therefore, the −OH interlayer termination near the edge may not serve as a hydrogen source, so hydrogen from water splitting or hydronium is required in neutral and acidic systems, respectively, for N_2_ reduction.

**Figure 5:**
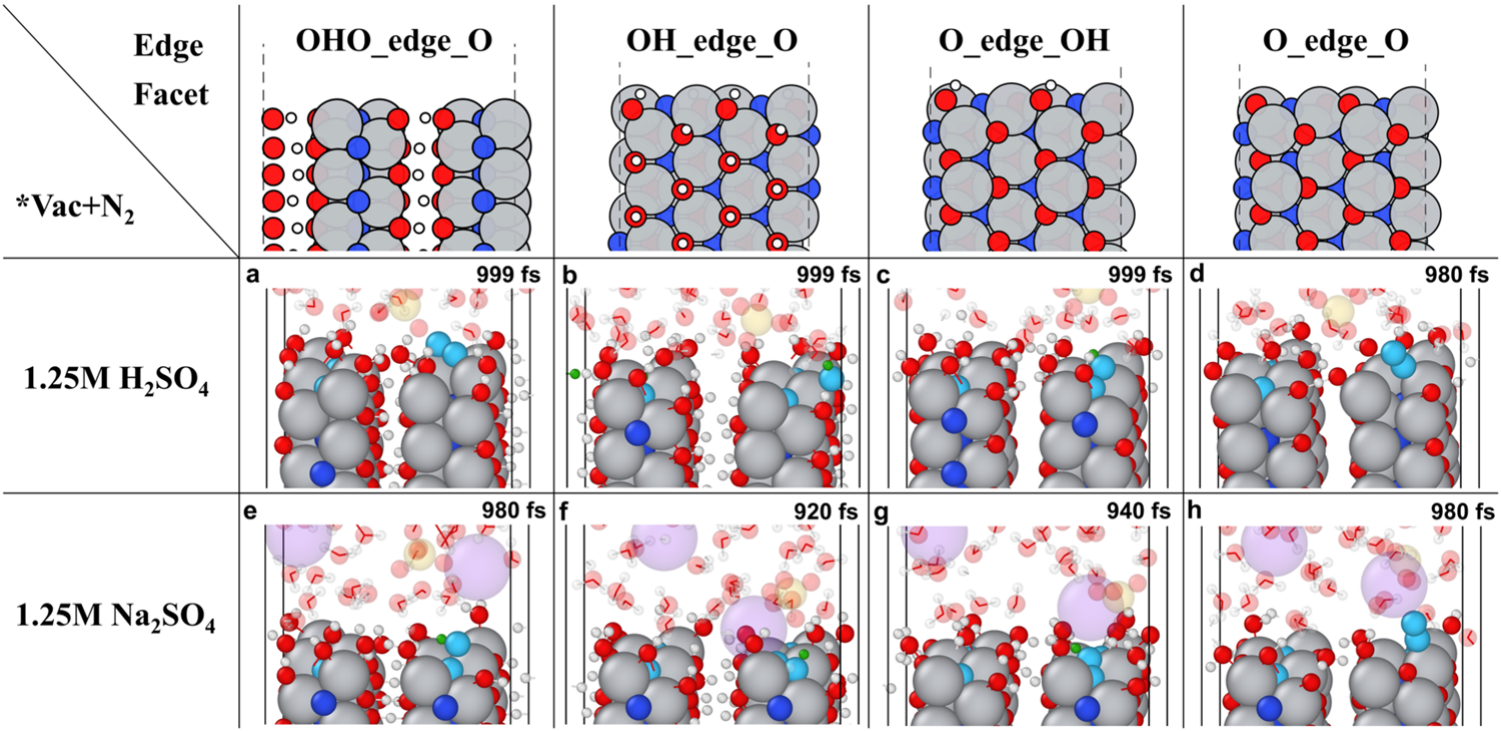
Summary figure of 1 ps AIMD calculation for MvK mechanism on different Ti_2_NT_*x*_ edge facets in 2 × 2 × 9 supercell (a–d). in neutral (1.25 M H_2_SO_4_) and in (e–h). acidic electrolyte (1.25 M NaSO_4_) with nitrogen vacancy ( $\theta =\frac{1}{4}$ ) and adsorbed N_2_ initially near the vacancy. Illustrations are selected at different snapshots for better visualization. (a). Instead of filling *_vac_, N_2_ went to *_bri_ without undergoing NRR. (b). Proceeded and formed NH_2_ and NH. (c). Formed N_2_H while maintaining the N–N bond. (d). N_2_ located on *_bri_ and approached *_vac_ without undergoing NRR. (e). N_2_H formed while partially embedding into *_vac_. (f). N_2_ fully embedded horizontally into *_vac_ and formed N_2_H. (g). N_2_H formed while partially embedding into *_vac_. (h). N_2_ stayed on Ti_top_ without undergoing NRR. Color code: gray, red, blue, cyan, white, green, yellow, and purple represent Ti, O, N below the first layer, N above the first layer, H, and H bonded with N, S, and Na, respectively.

### Presence of Nearby NH_3_ Adsorption Effect on MvK Mechanism

We investigate the influence of pH and nearby NH_3_ on the MvK mechanism on different Ti_2_NT_*x*_ MNene edge surfaces (Figures 4a–l, S28, S30–S33, S35–S37, S40–S43, Supporting Information) and summarized in Figures 5 and S44. Overall, we found that higher hydronium concentrations accelerate N_2_ reduction but impede vacancy replenishment. SO_4_^2–^ ions stabilize hydronium and facilitate proton transfer. AIMD studies reveal that NH_3_ desorption and hopping occur on Ti_2_N­(OH)­O surfaces, while neither NH_3_ nor H_2_O embeds defects in all kinds of Ti_2_NT_*x*_ surface. NH_3_ can act as a proton source for nearby NH or NH_2_ hydrogenation (Figures S26, S27, S41, and S45–S48 Supporting Information). The presence of NH_3_ on the surface promotes traditional NRR pathways over vacancy filling, while N_2_ and N_2_H filling is more favorable in the absence of NH_3_. The complex interactions among NH_3_, N_2_, and N_2_H in desorption and vacancy filling during NRR are further discussed in the Supporting Information.

### Stability of N Vacancies during the MvK Mechanism and Catalyst Regeneration

A shortcoming of catalysts that utilize the MvK mechanism is vacancy instability, leading to material degradation. Therefore, it is necessary to investigate the nitrogen vacancy stability; thus, Raman and operando XAS experiments were performed. To analyze the capability for N_2_ replenishment, Raman analysis was conducted on the same electrode before and after 68 h of Ar-saturated NRR to induce N vacancy formation, then after 68 h of additional N_2_-saturation to replenish the N vacancies (Figure 6). This cyclic behavior is observed via analyzing 19 locations on the electrode. On the pristine electrode (Figures 6a, S49a), 17 of the 19 spots present a spectrum that aligns with the expected Ti_2_NT*_*x*_* spectrum, while 2 match that of the Ti_2_NT*_*x*_* MNene with an emphasized Ti–O vibration peak. This enhanced Ti–O peak aligns with that from TiO_2_ materials, therefore the spectra were labeled as a hybrid Ti_2_NT*_*x*_*/TiO_2_. Following 68 h under Ar-saturated NRR conditions, the electrode was removed from the cell, briefly dried, and analyzed (Figures 6b, S49b). Of the 19 spectra, 6 aligned with pristine Ti_2_NT*_*x*_* material and 13 aligned with the hybrid Ti_2_NT*_*x*_*/TiO_2_ material. This demonstrates that lattice N is being extracted, forming N vacancies, which are then temporarily filled with oxygen. Finally, the electrode was placed into a fresh cell with electrolyte, and 68 h of NRR conditions were applied with N_2_-saturation to replenish the generated N vacancies (Figures 6c, S49c). Afterward, 16 of the 19 spectra were converted back to pristine Ti_2_NT*_*x*_*, and 3 resembled the hybrid material.

**Figure 6:**
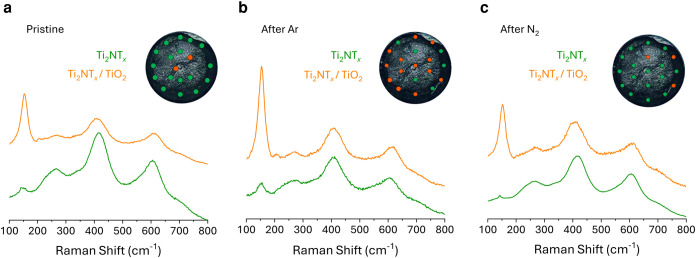
Raman white light images (inset) and spectra of Ti_2_NT*_*x*_* MNene coated on glassy carbon electrode (a) prior to NRR conditions, (b) after 68 h NRR under Ar-saturation to generate N vacancies, and (c) after 68 h NRR under N_2_-saturation after Ar-saturation to replenish previous N vacancies. Color coding on the white light images corresponds to the Raman spectrum immediately to the right.

To further investigate the stability of nitrogen vacancy creation and replenishment, we performed FT-EXAFS analysis of the catalyst under various atmospheres to monitor real-time structural changes (Figure 7a–c). The corresponding XANES data are shown in Figure S51. Under a continuous N_2_ feed (Figure 7a), the material largely retains its structural integrity instead of bulk oxidation, with only minor shifts in Ti–N/Ti-O bond lengths and cyclic changes in the Ti–N–Ti network, consistent with the formation and replenishment of vacancies. Notably, the final spectra closely match the initial, indicating the preservation of the local coordination environment after 2 h of reaction time. The EXAFS features at short radial distances ∼0.9 Å in Figures 1e and 7 are regarded as nonphysical, arising from Fourier transform artifacts due to limited k-space resolution and do not correspond to real interatomic distances (Figure S50).

**Figure 7:**
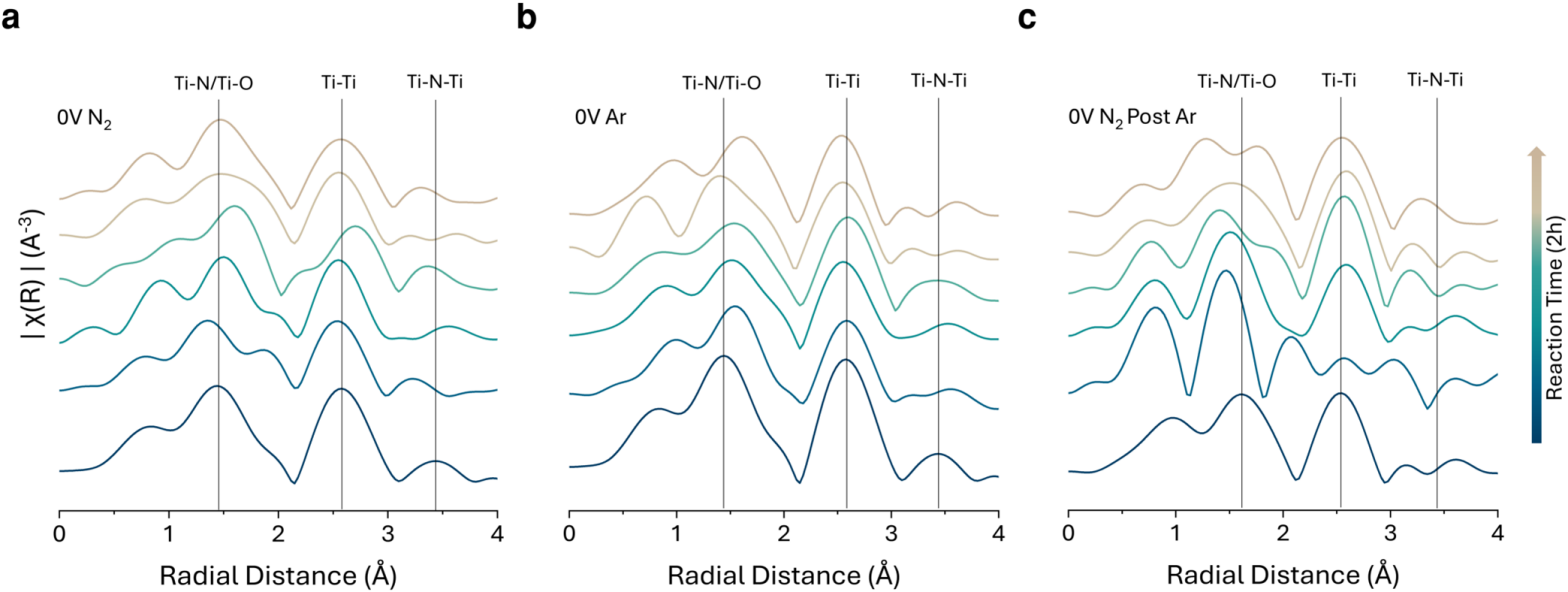
Operando Fourier transform of the normalized EXAFS spectra for Ti_2_NT*_*x*_* after being exposed to (a). N_2_ as a reactant gas, (b). Ar as an inert atmosphere gas, and (c). N_2_ reintroduction as a regeneration gas. A k-weight of 2 was used for EXAFS fitting.

When the feed gas is switched to Ar (Figure 7b), vacancy formation persists; however, in the absence of N_2_, these vacancies remain unfilled, leading to oxide formation as indicated by post-reaction spectra resembling TiO_2_ (Figure S2, Supporting Information). Reintroducing N_2_ to the system regenerates the pristine MNene structure as the material undergoes catalytic cycles to produce NH_3_ (Figure 7c). The resemblance of the final spectrum in Figure 7c to the initial spectrum in Figure 7a and to the spectrum in Figure 1e further supports this interpretation. Nevertheless, these results highlight the dynamic interplay between vacancy creation, temporary oxide formation, and structural regeneration, underscoring the material’s resilience and catalytic adaptability.

A continuous N_2_-purging long-term experiment demonstrated sustained NH_3_ formation over 100 h, while also maintaining decently stable current density (Figure S52a). Moreover, XRD analysis of the Ti_2_NT_*x*_-coated GDL before and after the 100 h NRR experiment confirmed the retention of the MNene structure (Figure S52b). Raman mapping of the electrode before and after the 100 h NRR, performed over a 1 × 1 cm^2^ area with 441 data points, also indicated the preservation of Ti–N bonds (Figure S52c, S52d). In addition, N_2_/Ar feed cycling was also carried out for 14 h with the cycle lasting 2 h (Figure S53). Postelectrolysis XRD and Raman also suggest the preservation of Ti_2_NT_*x*_ structure after N_2_/Ar cycling, which is consistent with the results shown in Figure 6. Together, these studies only support the presence of a stable MvK cycle and highlight the reversible interplay between Ti–N and Ti–O bonding. However, to fully understand the structural and morphological stability, including possible surface reconstruction or compositional changes, operando and postelectrolysis characterizations should be considered.

### Vacancy Filling Stability during MvK Mechanism

To compare the possible oxide formation at vacancy sites, we conducted adsorption free energy calculations for O_2_/N_2_/Na in the vacancy sites (Table S2, Supporting Information). Overall, the adsorption energy is smaller on Ti_2_NO_2_ (O_edge_O) for all adsorption sites, and the adsorption tendency for refilling MvK surfaces is O_2_ ≫ N_2_ > Na for all studied surfaces. The enhanced adsorption affinity of the O_2_ bond is primarily attributed to the breaking of the O_2_ bond, with one oxygen atom occupying the nitrogen vacancy site and the other occupying the Ti–Ti bridge site. This phenomenon implies potential deactivation due to oxide formation during the MvK mechanism, but as demonstrated earlier, the catalyst can be regenerated with N_2_ to continue the catalytic cycle.

### DFT Insights into Pair Distributions during MvK Mechanism Filling– Exploring Layer Changes, Charges, and Varied Adsorptions/Vacancies

To investigate changes in the pairwise radial distribution during the MvK mechanism, we analyzed Ti–O, Ti–N, and Ti–Ti pair distances using a combination of DFT calculations and experimental EXAFS data (Figure 8). Median pairwise distances were found to be approximately 1.8–2.2 Å for Ti–O pairs, 2.0–2.3 Å for Ti–N pairs, 2.7–3.3 Å for first-neighbor Ti–Ti pairs, and 3.9–4.5 Å for second-neighbor Ti–Ti pairs (Ti–N–Ti).

**Figure 8:**
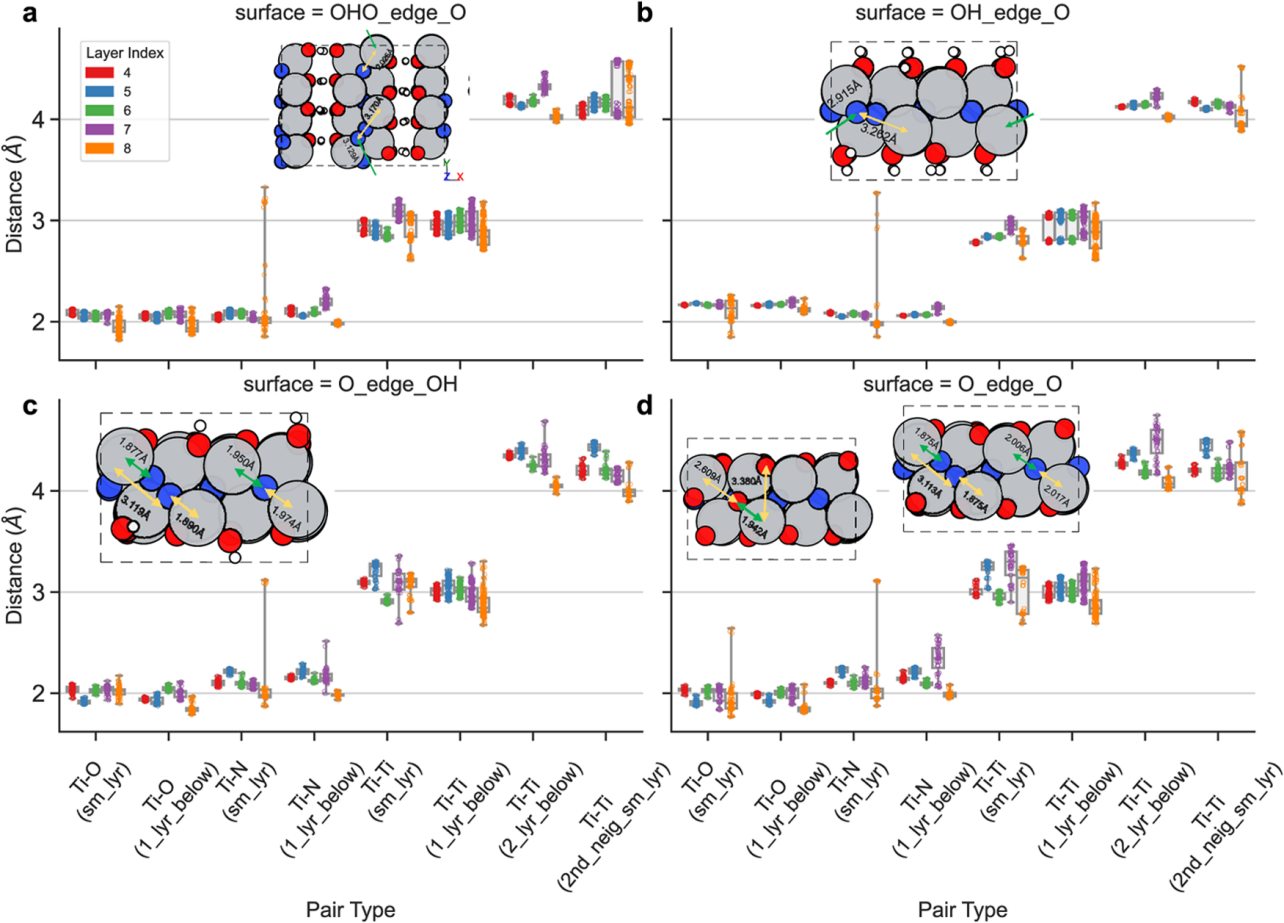
Pairwise distance boxplot of Ti–O/Ti-N/Ti–Ti of different coordinates grouped by surface layer index (bottom layer 0, top layer 8), pairwise type, and different Ti_2_NT_*x*_ surface including (a). surface Ti_2_N­(OH)O (OHO_edge_O), (b). surface Ti_2_N­(OH)_2_ (OH_edge_O), (c). surface Ti_2_NO_2_ with edge −OH termination (O_edge_OH), and (d). surface Ti_2_NO_2_ (O_edge_O). For each boxplot, pairwise distance of different adsorption type and charge (includes 0, 1, 4 extra electrons for 1 × 2 × 9 slab model; 0, 2, 8 extra electrons for 2 × 2 × 9 slab model for bilayer model) from DFT optimized geometry are included. Each hollow circle represents an overlap for each pair count on each boxplot. It should be noted that we only include the pair distance analysis from layer 4 to 8 due to the fixation of atoms from layers 0 to 3. Neutral charged optimized geometries for different adsorption types (Figures S54–S57, Supporting Information) explain the pairwise distance change of Ti–N due to the MvK mechanism at the vacancy filling step and possibly deactivation by oxygen vacancy filling for the TiNO_2_ (O_edge_O) surface.

For Ti–N pairs, a wide distribution and binodal trends were observed across all of the studied surfaces. These trends correspond to nitrogen positions in either half-embedded N_2_ molecules (2.8–3.1 Å from Ti–N pairs; Figure 8a,b) or fully embedded N_2_ molecules (3.1–3.2 Å; Figure 8c,d). Despite a systematic offset between DFT and EXAFS results, these findings align with the increased peaks and fluctuations observed in Figure 7a,c, which suggest Ti–N and Ti–O bonds between 1.5 and 2 Å.

Variations in Ti–Ti pair distances were more prominent in the top two layers, where vacancy-induced relaxation influenced neighboring Ti atoms up to the second coordination shell. The observed shifts in the Ti–N–Ti peak (EXAFS) were primarily attributed to outer-edge surface relaxation. A violin plot comparison of adsorption types further corroborated these observations, confirming that radial changes stemmed from nitrogen vacancy filling by N_2_ or O_2_ rather than surface charging effects (Figures 8a–d, S54–S57).

An intriguing observation was the broad range of same-layer Ti–O distances, attributed to O_2_ filling N vacancies. In Ti_2_NO_2_, for instance, stabilization occurred via interlayer oxygen atoms moving closer to the edge surface (Figures 8d, S54d). Convergence tests (Figure S58a–c, Supporting Information) on the Ti_2_NO_2__(sur-N) surface revealed that edge stabilization was achieved when oxygen coordinated with three Ti atoms rather than two, a behavior unique to Ti_2_NO_2_.

For surfaces with −OH terminations, hydrogen bonding increased interlayer interactions, reducing edge oxygen electronegativity, and stabilizing −OH terminations. This stabilization prevented the deactivation of nitrogen vacancy sites, thereby preserving catalytic functionality. These findings highlight the critical role of vacancy interactions and terminations in governing the catalytic stability of Ti-based systems for NH_3_ synthesis.

## Conclusions

This work demonstrates a novel mechanism for NRR electrocatalysis using an emerging class of 2D MNene materials. In neutral aqueous conditions (0.1 M Na_2_SO_4_), the MNene catalyst achieved an FE of 47.5 ± 2.8%. Comprehensive analyses, including isotopic labeling and operando XAS, DFT, and AIMD simulations, revealed that the MvK mechanism is both thermodynamically favorable and kinetically efficient, with nitrogen vacancies regenerated by N_2_ in the electrolyte. Proposed pathways (MvK_as_, MvK_as‑dis_, and MvK_dis_) highlight the critical role of edge and interlayer terminations in the catalytic activity. These findings establish MNenes as a powerful platform for electrochemical NH_3_ synthesis, offering significant insights into NRR mechanisms and advancing the development of sustainable, carbon-free ammonia production technologies.

## Experimental Section

### Synthesis of Ti_2_NT_*x*_ MNene

Ti_2_AlN MAX phase was synthesized via ambient pressure sintering. Ti and AlN were mixed and ground in a 2:1 molar ratio for 10 min. The mix was then placed in a tube furnace and heated to 1600 °C at a ramp rate of 10 °C min^–1^ and held for 1 h under continuous Ar flow. The resulting Ti_2_AlN pellet was ground in preparation for etching to MNene.

The Ti_2_AlN MAX phase was etched through an oxygen-assisted molten salt fluoride etching technique as previously utilized.^46^ In short, the MAX phase was mixed in a 1:1 mass ratio with a 59:29:12 wt % mixture of KF, LiF, and NaF, respectively. This mixture was then placed in a tube furnace and heated to 550 °C at a ramp rate of 10 °C min^–1^ under an Ar flow. The furnace was kept at 550 °C for 5 h, where halfway through the Ar flow was cut off and the furnace was sealed to the environment. The sealed furnace was then cooled to 450 °C and maintained there for 50 min before cooling to room temperature. The molten salt-treated MAX phase (Ti_2_AlN-MST) was collected for acid washing to remove excess salts and etched species.

The Ti_2_AlN-MST was washed in 4 M formic acid for 45 min before being filtered and washed with water until a neutral supernatant was achieved. The material was then dried in a vacuum oven at 50 °C overnight, and the multilayer material (Ti_2_NT*_*x*_* ML) was collected.

The Ti_2_NT*_*x*_* ML was delaminated via an intercalation and sonication strategy. Tetramethylammonium hydroxide was used as the intercalating agent by mixing for 4 h. The suspension was then sonicated for 30 min before centrifuging at 3500 rpm until a neutral supernatant was achieved. The material was then resuspended in a small amount of water and left to rest to allow the remaining MAX phase to precipitate from suspension. The colored supernatant was filtered and dried in a vacuum oven at 50 °C overnight.

### Material Characterization

Crystalline structure of the material was analyzed by using X-ray diffraction (XRD, Rigaku Miniflex 6G). Raman spectroscopy was performed with a Renishaw inVia Qontor equipped with a 532 nm excitation wavelength laser, an 1800 lines mm^–1^ grating, and a 50× long objective lens. XAS measurements were performed in fluorescence mode at the multipurpose beamline for spectroscopy, 12-BM, at the Advanced Photon Source (APS) located at the Argonne National Laboratory (ANL). A defined beam size of 0.5 × 0.8 mm^2^ using slits and an incident photon flux of ∼10^11^ photons s^–1^ were used. XANES data were collected in the vicinity of the Ti K-edge (4966 eV) at ambient temperature. Ti foil, TiH_2_, TiN, and TiO_2_ rutile were investigated in fluorescence to obtain the reference spectra. XAS data were processed by using the Demeter software package with the built-in AUTOBK algorithm used to normalize the absorption coefficient.

### Electrode Preparation for Electrochemistry

MNene was mixed into a mixture of ethanol and 5 wt % Nafion in ethanol and then sonicated for 30 min. This suspension was then dropcast onto a glassy carbon electrode and dried for 30 min at 50 °C under vacuum.

### Electrochemical Measurements, Product Quantification, and Determination of Performance

The methods for electrochemical measurements were detailed previously and summarized here.^17^ Additionally, suggested protocols from the literature were employed to ensure that produced NH_3_ did not originate from contamination sources.^47,48^ To avoid potential NOx contaminants, 0.1 M KOH base trap and 0.05 M H_2_SO_4_ acid trap were used to purify the N_2_ gas feed (ultrahigh pure, 99.999%). A photograph of the NRR electrochemical setup is provided in Figure S64. The counter and reference electrodes were graphite and Ag/AgCl, respectively. A Nafion-117 membrane was used to separate the anode and cathode chambers of the H-cell. Measurements were conducted using a Bio-Logic SP-300 potentiostat.

NH_3_ concentration was detected via UV–vis spectroscopy using the indophenol blue method, and FE was calculated using the same methods previously discussed.^17,46^

### Isotopic Exchange Experiments

Isotopic exchange experiments were performed in a semibatch process to minimize gas usage to the system. ^14^N_2_ and ^15^N_2_ gases were pressurized to the back of a gas diffusion layer electrode with MNene material spray-coated on. The electrolyte was collected and analyzed via proton nuclear magnetic resonance (1H-NMR) spectroscopy with a 400 MHz Bruker Avance III system. For the ^15^N_2_ measurements shown in Figure 3, the electrode was deliberately removed from the electrochemical cell after each 2 h run and immersed in fresh electrolyte for the subsequent run. The primary objective of this experimental design was to demonstrate that via the MvK mechanism, ^15^N_2_ gas substitutes lattice ^14^N within the catalyst. When the ^15^N-substituted electrode is placed into fresh electrolyte, the ammonia formed originates from lattice ^15^N, as confirmed by the data in Figure 3. This provides direct evidence of the lattice substitution.

### DFT Optimization of Structures

The research utilized first-principles methods implemented in the Vienna Ab initio Simulation Package (VASP)^49^ to carry out all computations. Density functional theory (DFT) was employed within the generalized gradient approximation (GGA), Perdew–Burke–Ernzerhof (PBE) functional^50^ for electron correlation, and the projector augmented wave (PAW) pseudopotentials for electron–ion interactions.^51^ Structural optimization was sampled in the Brillouin zone with the Monkhorst–Pack method^52^ using 2 × 3 × 1 for (system 1 × 2 × 9) and 2 × 2 × 1 for (system 2 × 2 × 9) k-point grid. The cutoff energy of 500 eV is deployed, and the convergence tolerance of force is set to be 0.01 eV A^–1^, and 10^–5^ eV for energy with a Gaussian smearing width of 0.1 eV. The empirical DFT-D3 correction^53^ was utilized to describe the VDW interactions to avoid the potential errors of physical adsorption. Moreover, spin polarization is used to accurately describe the electron distribution of Ti_2_NT*_*x*_*.^33,54–56^ The free energy correction terms of the gas phase, including zero-point energy correction and vibrational entropy, are implemented using normal-mode analysis as in previous work.^57,58^

### Ti_2_NT_*x*_ DFT Edge MNene Model Setup Feasibility

The details of convergence test for the edge slab model and adsorption energy *E*_ads_ and N vacancy formation energies *E*_vac_ are shown in Figure S58 and Table S3 (Supporting Information). Overall, we found that the 1 × 2 × 9 slab model with the fixbot4 setup can maintain the change of adsorption energy within 0.02 eV when we further increase system layers. Whereas, when we consider the Ti_2_N­(OH)O MNene (OHO_edge_O model), we employed the 2 × 2 × 9 slab model to keep vacancy defect ratio at 50% for each surface, which is further explained in the Supporting Information.

### Ab Initio Molecular Dynamics (AIMD)

AIMD simulations were employed to test the nitrogen vacancy filling and ammonia desorption for the MvK mechanism. Dynamics were run with the NVT ensemble at a temperature of 298 K with the energy convergence tolerance set to 10^–4^ eV, without a force convergence tolerance. The Nose thermostat with a Nose-mass parameter of 0.5 was used with a time step of 1 fs, totaling 1 ps over each simulation.

The simulation cell was reoptimized using the supercell of our previous work’s unit cell.^36^ Four different surface terminations were analyzed, starting from Ti_2_NO_2_ to Ti_2_NO_2_ with an edge −OH surface termination, Ti_2_N­(OH)_2_, and the bilayer model of Ti_2_N­(OH)O (Figure S54a, S55a, S56a, S57a, Supporting Information). To prevent interaction between the cleaved surface and the periodic system, a 10 Å vacuum layer was added after cleaving the surface along the edge direction of the MNene. To simulate the electrolyte system, we employed pure water, 2.5 M Na_2_SO_4_, and 1.25 M Na_2_SO_4_ to represent a neutral electrolyte system. Additionally, 2.5 M H_2_SO_4_ and 1.25 M H_2_SO_4_ solutions were utilized to simulate the acidic electrolyte system. The quantities of solutes were determined based on the water density at 298 K (0.997 g cm^–3^). To achieve a 2.5 M solution system, only one salt molecule was required due to the cell’s volume. As for simulating 1.25 M solution systems, a larger supercell with twice the length along the basal plane was created. To shorten diffusion distance onto surface in simulation, the adsorbed molecules N_2_ and/or NH_3_ were placed near the Ti_2_NT_*x*_ vacancy site on the surface, and then salt was placed around 5 Å away from the surface. Subsequently, solvent molecules (H_2_O) were added using the amorphous packing tool in Materials Studio Software^59^ to pack the system.

## Supplementary Material



## Notes

Computational methods; proposed electrochemical reaction mechanism of Mars-van Krevelen mechanism; XANES and EXAFS spectra for reference materials; CV and LSV of MNene in 0.1 M Na_2_SO_4_; chronoamperometry curves; ammonia quantification; ammonia yield and Faradaic efficiency in various neutral electrolytes; NRR experiments using gas diffusion layer electrodes; postelectrolysis characterizations; ^1^H NMR quantification; NRR using ^15^N_2_ isotope; AIMD snapshots and free energy diagrams, and long-term studies using Ar and N_2_ gases (PDF)

## References

[1] Chen G.-F., Cao X., Wu S., Zeng X., Ding L.-X., Zhu M., Wang H. (2017). Ammonia Electrosynthesis
with High Selectivity under Ambient Conditions
via a Li^+^ Incorporation Strategy. J. Am. Chem. Soc..

[2] Krishnamurthy D., Lazouski N., Gala M. L., Manthiram K., Viswanathan V. (2021). Closed-Loop Electrolyte Design for
Lithium-Mediated
Ammonia Synthesis. ACS Cent. Sci..

[3] Mao X., Bai X., Wu G., Qin Q., O’Mullane A. P., Jiao Y., Du A. (2024). Electrochemical
Reduction of N_2_ to Ammonia Promoted by Hydrated Cation
Ions: Mechanistic
Insights from a Combined Computational and Experimental Study. J. Am. Chem. Soc..

[4] Paul S., Sarkar S., Adalder A., Kapse S., Thapa R., Ghorai U. K. (2023). Strengthening the
Metal Center of Co–N_4_ Active Sites in a 1D–2D
Heterostructure for Nitrate and Nitrogen
Reduction Reaction to Ammonia. ACS Sustainable
Chem. Eng..

[5] Yao C., Guo N., Xi S., Xu C.-Q., Liu W., Zhao X., Li J., Fang H., Su J., Chen Z. (2020). Atomically-precise
dopant-controlled single cluster catalysis for electrochemical nitrogen
reduction. Nat. Commun..

[6] Ling C., Zhang Y., Li Q., Bai X., Shi L., Wang J. (2019). New Mechanism for N_2_ Reduction:
The Essential Role of
Surface Hydrogenation. J. Am. Chem. Soc..

[7] Koestoer R. H., Ligayanti T., Kartohardjono S., Susanto H. (2024). Down-streaming Small-Scale
Green Ammonia to Nitrogen-Phosphorus Fertilizer Tablets for Rural
Communities. Emerging Sci. J..

[8] Wang M., Khan M. A., Mohsin I., Wicks J., Ip A. H., Sumon K. Z., Dinh C.-T., Sargent E. H., Gates I. D., Kibria M. G. (2021). Can sustainable
ammonia synthesis pathways compete
with fossil-fuel based Haber–Bosch processes?. Energy Environ. Sci..

[9] Ren Y., Li S., Yu C., Zheng Y., Wang C., Qian B., Wang L., Fang W., Sun Y., Qiu J. (2024). NH_3_ Electrosynthesis
from N_2_ Molecules: Progresses, Challenges,
and Future Perspectives. J. Am. Chem. Soc..

[10] Zhang X., Xiong W., Wang T., Chai E., Lin J., Huang L., Feng Y., Wu M., Wang Y. (2024). Cascade electrosynthesis
of LiTFSI and N-containing analogues via a looped Li–N_2_ battery. Nat. Catal..

[11] Xue Z.-H., Zhang S.-N., Lin Y.-X., Su H., Zhai G.-Y., Han J.-T., Yu Q.-Y., Li X.-H., Antonietti M., Chen J.-S. (2019). Electrochemical Reduction of N_2_ into NH_3_ by Donor–Acceptor Couples of Ni
and Au Nanoparticles
with a 67.8% Faradaic Efficiency. J. Am. Chem.
Soc..

[12] Ye T.-N., Park S.-W., Lu Y., Li J., Sasase M., Kitano M., Hosono H. (2020). Contribution of Nitrogen Vacancies
to Ammonia Synthesis over Metal Nitride Catalysts. J. Am. Chem. Soc..

[13] Fu X., Niemann V. A., Zhou Y., Li S., Zhang K., Pedersen J. B., Saccoccio M., Andersen S. Z., Enemark-Rasmussen K., Benedek P. (2024). Calcium-mediated
nitrogen reduction for electrochemical
ammonia synthesis. Nat. Mater..

[14] Li L., Xu L., Wang H., Wei H., Tang C., Li G., Dou Y., Liu H. K., Dou S. X. (2024). Electrocatalytic nitrogen cycle:
mechanism, materials, and momentum. Energy Environ.
Sci..

[15] Wang M., Liu S., Qian T., Liu J., Zhou J., Ji H., Xiong J., Zhong J., Yan C. (2019). Over 56.55% Faradaic
efficiency of ambient ammonia synthesis enabled by positively shifting
the reaction potential. Nat. Commun..

[16] Shen P., Li X., Luo Y., Zhang N., Zhao X., Chu K. (2022). Ultra-efficient
N_2_ electroreduction achieved over a rhodium single-atom
catalyst (Rh_1_/MnO_2_) in water-in-salt electrolyte. Appl. Catal., B.

[17] Johnson D., Hunter B., Christie J., King C., Kelley E., Djire A. (2022). Ti_2_N nitride MXene evokes
the Mars-van Krevelen mechanism
to achieve high selectivity for nitrogen reduction reaction. Sci. Rep..

[18] Daisley A., Hargreaves J. S. J. (2023). Metal nitrides, the Mars-van Krevelen mechanism and
heterogeneously catalysed ammonia synthesis. Catal. Today.

[19] Shetty A. U., Sankannavar R. (2024). Exploring nitrogen reduction reaction
mechanisms in
electrocatalytic ammonia synthesis: A comprehensive review. J. Energy Chem..

[20] Yuan H., Zhu C., Hou Y., Yang H. G., Wang H. (2024). Optimizing the Lattice
Nitrogen Coordination to Break the Performance Limitation of Metal
Nitrides for Electrocatalytic Nitrogen Reduction. JACS Au.

[21] Zeinalipour-Yazdi C. D., Hargreaves J. S. J., Catlow C. R. A. (2015). Nitrogen Activation in a Mars–van
Krevelen Mechanism for Ammonia Synthesis on Co_3_Mo_3_N. J. Phys. Chem. C.

[22] Hunter S. M., Gregory D. H., Hargreaves J. S. J., Richard M., Duprez D., Bion N. (2013). A Study of ^15^N/^14^N Isotopic Exchange over Cobalt
Molybdenum Nitrides. ACS Catal..

[23] He H.-y., Wang S., Ji L.-l. (2022). Fabrication
of self-supported Cu_3_N electrode for electrocatalytic nitrogen
reduction reaction. J. Fuel Chem. Technol..

[24] Liu L., Min L., Zhang W., Wang Y. (2021). Dual roles of graphitic carbon nitride
in the electrosynthesis of ammonia under ambient conditions. J. Mater. Chem. A.

[25] Zhao J., Chen Z. (2017). Single Mo Atom Supported
on Defective Boron Nitride Monolayer as
an Efficient Electrocatalyst for Nitrogen Fixation: A Computational
Study. J. Am. Chem. Soc..

[26] Chen J. G. (1996). Carbide
and Nitride Overlayers on Early Transition Metal Surfaces: Preparation,
Characterization, and Reactivities. Chem. Rev..

[27] Tang S., Dang Q., Liu T., Zhang S., Zhou Z., Li X., Wang X., Sharman E., Luo Y., Jiang J. (2020). Realizing
a Not-Strong-Not-Weak Polarization Electric Field in Single-Atom Catalysts
Sandwiched by Boron Nitride and Graphene Sheets for Efficient Nitrogen
Fixation. J. Am. Chem. Soc..

[28] Oyama S. T. (1992). Preparation
and catalytic properties of transition metal carbides and nitrides. Catal. Today.

[29] Zhang Z., Feng X., Zhang Z., Chen L., Liu W., Tong L., Gao X., Zhang J. (2024). Graphdiyne Enabled
Nitrogen Vacancy Formation in Copper Nitride for Efficient Ammonia
Synthesis. J. Am. Chem. Soc..

[30] Li Z., Attanayake N. H., Blackburn J. L., Miller E. M. (2021). Carbon dioxide and
nitrogen reduction reactions using 2D transition metal dichalcogenide
(TMDC) and carbide/nitride (MXene) catalysts. Energy Environ. Sci..

[31] Yang X., Nash J., Anibal J., Dunwell M., Kattel S., Stavitski E., Attenkofer K., Chen J. G., Yan Y., Xu B. (2018). Mechanistic
insights into electrochemical nitrogen reduction reaction
on vanadium nitride nanoparticles. J. Am. Chem.
Soc..

[32] Yang X., Kattel S., Nash J., Chang X., Lee J. H., Yan Y., Chen J. G., Xu B. (2019). Quantification of Active Sites and
Elucidation of the Reaction Mechanism of the Electrochemical Nitrogen
Reduction Reaction on Vanadium Nitride. Angew.
Chem., Int. Ed..

[33] Xie Y., Kent P. (2013). Hybrid density functional
study of structural and electronic properties
of functionalized Ti_n+1_X_n_ (X = C, N) monolayers. Phys. Rev. B.

[34] Zhang N., Hong Y., Yazdanparast S., Zaeem M. A. (2018). Superior structural,
elastic and electronic properties of 2D titanium nitride MXenes over
carbide MXenes: a comprehensive first principles study. 2D Mater..

[35] Onyia I. C., Ezeonu S. O., Bessarabov D., Obodo K. O. (2021). Density functional
theory studies of transition metal doped Ti_3_N_2_ MXene monolayer. Comput. Mater. Sci..

[36] Lai H.-E., Yoo R. M., Djire A., Balbuena P. B. (2024). Investigation of
the Vibrational Properties of 2D Titanium Nitride MXene Using DFT. J. Phys. Chem. C.

[37] Zhang T., Zheng Z., Lu H., Liu H., Chen G., Xia S., Zhou L., Qiu M. (2023). Rational design
of periodic porous
titanium nitride MXene as a multifunctional catalytic membrane. Nanoscale.

[38] Yang X., Gao N., Zhou S., Zhao J. (2018). MXene nanoribbons as electrocatalysts
for the hydrogen evolution reaction with fast kinetics. Phys. Chem. Chem. Phys..

[39] Jiang B., Yang T., Wang T., Chen C., Yang M., Yang X., Zhang J., Kou Z. (2022). Edge stimulated hydrogen
evolution reaction on monodispersed MXene quantum dots. Chem. Eng. J..

[40] Vénosová B., Karlický F. (2023). Modeling size
and edge functionalization of MXene-based
quantum dots and their effect on electronic and magnetic properties. Nanoscale Adv..

[41] Lützenkirchen-Hecht D., Wagemaker M., Keil P., van Well A. A., Frahm R. (2003). Ex situ reflection
mode EXAFS at the Ti K-edge of lithium intercalated TiO_2_ rutile. Surf. Sci..

[42] Timchenko N. A., Zubavichus Y. V., Krysina O. V., Kuznetsov S. I., Syrtanov M. S., Bondarenko S. V. (2016). Local structure
of titanium nitride-based
coatings. J. Surf. Invest.: X-Ray, Synchrotron
Neutron Tech..

[43] Djire A., Zhang H., Reinhart B. J., Nwamba O. C., Neale N. R. (2021). Mechanisms
of Hydrogen Evolution Reaction in Two-Dimensional Nitride MXenes Using
In Situ X-Ray Absorption Spectroelectrochemistry. ACS Catal..

[44] Rosli Z. D., Tesana S., Malone N., Ahmed M. I., Jovic V., Giddey S., Kennedy J. V., Gupta P. (2025). Adapting Stringent
Electrochemical Nitrogen Reduction Protocols for Catalysts with Ultralow
Production Rates. ACS Catal..

[45] Greenlee L. F., Renner J. N., Foster S. L. (2018). The use
of controls for consistent
and accurate measurements of electrocatalytic ammonia synthesis from
dinitrogen. ACS Catal..

[46] Johnson D., Djire A. (2023). Effect of pH on the
Electrochemical Behavior and Nitrogen Reduction
Reaction Activity of Ti_2_N Nitride MXene. Adv. Mater. Interfaces.

[47] Tang C., Qiao S.-Z. (2019). How to explore ambient
electrocatalytic nitrogen reduction
reliably and insightfully. Chem. Soc. Rev..

[48] Andersen S.
Z., Čolić V., Yang S., Schwalbe J. A., Nielander A. C., McEnaney J. M., Enemark-Rasmussen K., Baker J. G., Singh A. R., Rohr B. A. (2019). A rigorous electrochemical ammonia synthesis protocol
with quantitative isotope measurements. Nature.

[49] Kresse G., Furthmüller J. (1996). Efficient iterative schemes for ab initio total-energy
calculations using a plane-wave basis set. Phys.
Rev. B.

[50] Perdew J. P., Burke K., Ernzerhof M. (1996). Generalized
Gradient Approximation
Made Simple. Phys. Rev. Lett..

[51] Kresse G., Joubert D. (1999). From ultrasoft pseudopotentials to the projector augmented-wave
method. Phys. Rev. B.

[52] Monkhorst H. J., Pack J. D. (1976). Special points for
Brillouin-zone integrations. Phys. Rev. B.

[53] Grimme S., Ehrlich S., Goerigk L. (2011). Effect of the damping function in
dispersion corrected density functional theory. J. Comput. Chem..

[54] He X., Gui Y., Xie J., Li Q. (2020). First-Principles Study on the Potential
of Monolayer Ti_2_N as an Adsorbent for Dissolved H_2_ and C_2_H_2_ Gases in Oil. ACS Appl. Nano Mater..

[55] Limbu Y., Kaphle G. C., Karn A. L., Shah N. K., Paudyal H., Paudyal D. (2022). Electronic structure
and magnetism of pristine, defected,
and strained Ti_2_N MXene. J. Magn.
Magn. Mater..

[56] Gao G., Ding G., Li J., Yao K., Wu M., Qian M. (2016). Monolayer MXenes: promising
half-metals and spin gapless semiconductors. Nanoscale.

[57] Wang V., Xu N., Liu J.-C., Tang G., Geng W.-T. (2021). VASPKIT: A user-friendly
interface facilitating high-throughput computing and analysis using
VASP code. Comput. Phys. Commun..

[58] Skúlason E., Tripkovic V., Björketun M. E., Gudmundsdóttir S., Karlberg G., Rossmeisl J., Bligaard T., Jónsson H., Nørskov J. K. (2010). Modeling the electrochemical hydrogen oxidation and
evolution reactions on the basis of density functional theory calculations. J. Phys. Chem. C.

[59] Meunier M., Robertson S. (2021). Materials
studio 20th anniversary. Mol. Simul..

