# Supplementary material for: Lattice-Nitrogen-Mediated Chemistry Suppresses Hydrogen Evolution for Record Faradaic Efficiency in Ammonia Synthesis
Source: J Am Chem Soc. 2025 Jul 31;147(32):29327–39. doi: 10.1021/jacs.5c09104 (PMC12356539; doi:10.1021/jacs.5c09104)
Supplement: Supplementary file 1 [file ja5c09104_si_001.pdf]

Lattice-Nitrogen-Mediated Chemistry Suppresses Hydrogen Evolution for Record Faradaic Efficiency in Ammonia Synthesis

David Kumar Yesudoss[1#], Hao-En Lai[1, #], Denis Johnson[1], Mark Lee[2], Benjamin Reinhart[3], Perla B. Balbuena[1,4*], Abdoulaye Djire[1,5*]

[1]Artie McFerrin Department of Chemical Engineering, Texas A&M University, College Station, TX 77843, USA

[2]Wm Michael Barnes Department of Industrial and Systems Engineering, Texas A&M University, College Station, TX 77843, USA

[3]X-ray Science Division, Argonne National Laboratory, Argonne, Lemont, IL 60439, USA

[4]Department of Chemistry, Texas A&M University, College Station, TX 77843, USA

[5]Department of Materials Science and Engineering, Texas A&M University, College Station, TX 77843, USA

Corresponding Authors: balbuena@tamu.edu; adjire@tamu.edu

# indicates co-first author

Methods

Proposed electrochemical reaction mechanism of Mars–van Krevelen Mechanism

A heterogeneous Mars-van Krevelen (MvK) mechanism includes two main processes, where two ammonia (NH_3) molecules are formed in a complete reaction cycle. 1) An initial NH_3 molecule arises through the process of protonation of a surface N atom. 2) This vacancy is subsequently refilled by the dissolved N_2 from the electrolyte solution. The major benefit of the MvK mechanism is the N≡N energy barrier can be largely decreased after one of the N atoms bonds with the surface vacancy.

During the traditional electrochemical NRR, the half reactions at the cathode and anode are as follows: At the anode, protons (H^+) are solvated in electrolyte and transported from the anode to cathode through the electrolyte phase. The catalyst surface undergoes sequential hydrogenation with one H^+ and electron (e^-) added at a time from electrode through wire.

$$H_2 \leftrightharpoons 2H^+ + 2e^- \qquad (1)$$

The cathode reaction is:

$$N_2 + 6H^+ + 6e^- \leftrightharpoons 2NH_3 \qquad (2)$$

In this study, we focus on the cycle of replenishing the Ti_2NT_x MNene surface on the edge site. For the MvK mechanism in this study, we focus on 1) **associative Heyrovsky MvK mechanism** where reaction occurs fully on the N-site, 2) **dissociative Heyrovsky MvK mechanism** where N_2 dissociates on N-site and then undergoes associative on Ti-bridge site, 3) **associative-dissociative Heyrovsky MvK mechanism** where dissociation occurs after NH_2 forms and migrates to Ti bridge site to undergo the final step of NRR on the bridge site. Therefore, for the latter cycle of half reactions of replenishment in MvK mechanism, the half reactions at the anode remain

$$H_2 \leftrightharpoons 2H^+ + 2e^- \tag{1, revisited}$$

And the cathode reaction becomes

$$*_{vac} + N_2 + 3H^+ + 3e^- \leftrightharpoons * + NH_3 \tag{3}$$

where $*$ represents a surface site on top of the N-site.

First, if we consider the MvK mechanism reaction only on the nitrogen vacancy site without migration as **associative-MvK (MvK_{as})**, where $*$, $*_{vac}$, $*N_V$ represent surface site on top of N-site, the nitrogen vacancy, and surface site vacancy replenished with N_2, respectively.

$$*_{vac} + 3(H^+ + e^-) + N_2 \leftrightharpoons *N_V + 3(H^+ + e^-) \tag{4}$$

$$*N_V + 3(H^+ + e^-) \leftrightharpoons *N_VH + 2(H^+ + e^-) \tag{5}$$

$$*N_VH + 2(H^+ + e^-) \leftrightharpoons *N_VH_2 + (H^+ + e^-) \tag{6}$$

$$*N_VH_2 + (H^+ + e^-) \leftrightharpoons *N_VH_3 \tag{7}$$

$$*N_VH_3 \leftrightharpoons * + NH_3(g)\uparrow \tag{8}$$

Second, if we consider the dissociative Heyrovsky mechanism where Nitrogen molecule first dissociates after the vacancy is filled and moves to nearby bridge sites as **dissociative MvK (MvK_{dis})**, so the MvK mechanism becomes

$$*_{vac} + 3(H^+ + e^-) + N_2 \leftrightharpoons *N_V + 3(H^+ + e^-) \tag{4, revisited}$$

$$*N_V + 3(H^+ + e^-) \leftrightharpoons *_{bri}N_V + 3(H^+ + e^-) \tag{9}$$

$$*_{bri}N_V + 3(H^+ + e^-) \leftrightharpoons *_{bri}N_VH + 2(H^+ + e^-) \tag{10}$$

$$*_{bri}N_VH + 2(H^+ + e^-) \leftrightharpoons *_{bri}N_VH_2 + (H^+ + e^-) \tag{11}$$

$$*_{bri}N_VH_2 + (H^+ + e^-) \leftrightharpoons *_{bri}N_VH_3 \tag{12}$$

$$*_{bri}N_VH_3 \leftrightharpoons * + NH_3(g)\uparrow \quad (13)$$

where $*_{bri}$ represent the surface Ti bridge sites.

Third, during the Ab initio molecular dynamics simulation, we found the dissociation and diffusion of NH/NH_2 to different sites before completion of NRR to form NH_3. Therefore, if we further consider the dissociation of N_2H_2 on the vacancy site after two protonation steps and NH_2 molecule on original vacancy sites moves to nearby bridge sites as **associative-dissociative MvK ($MvK_{as\text{-}dis}$)**:

$$*_{vac} + 3(H^+ + e^-) + N_2 \leftrightharpoons *N_V + 3(H^+ + e^-) \quad \text{(4, revisited)}$$

$$*N_V + 3(H^+ + e^-) \leftrightharpoons *N_VH + 2(H^+ + e^-) \quad \text{(5, revisited)}$$

$$*N_VH + 2(H^+ + e^-) \leftrightharpoons *N_VH_2 + (H^+ + e^-) \quad \text{(6, revisited)}$$

$$*N_VH_2 + (H^+ + e^-) \leftrightharpoons *_{bri}N_VH_2 + (H^+ + e^-) \quad (14)$$

$$*_{bri}N_VH_2 + (H^+ + e^-) \leftrightharpoons *_{bri}N_VH_3 \quad \text{(12, revisited)}$$

$$*_{bri}N_VH_3 \leftrightharpoons * + NH_3(g)\uparrow \quad \text{(13, revisited)}$$

Electrochemical pH and Electrode potential correction for DFT profile

The reference potential is set to the standard hydrogen electrode (SHE). At pH = 0 and $U = 0\,V$, 1 bar of H_2 in gas phase at 298 K. The free energy change of intermediates ΔG_0 is calculated at zero potential and pH = 0 when reference to SHE as follows:

$$\Delta G_0 = \Delta E + \Delta E_{ZPE} - T \times \Delta S \quad (15)$$

Where ΔE is the reaction energy between two intermediate states, and ΔE_{ZPE} and ΔS are difference of zero-point energy and entropy between adsorbed species and gas phase molecules in products and reactant, respectively. These are calculated with normal mode analysis by DFT harmonic vibrational frequency calculations. When the free energy of the system is calculated under applied potentials, U, an applied potential bias, U, will be considered for all reactions involving electrons by shifting by free energy, -neU, where n is the number of electrons, and U < 0.

$$\Delta G(U) = \Delta G_0 - neU \quad (16)$$

For pH value different from 0, the free energy of H^+ should be corrected with concentration dependence of the entropy and simplified as $\Delta G(U,pH) = \Delta G(U) + k_BTln\alpha_{H^+}$

$$\Delta G(U,pH) = \Delta G_0 - neU + nk_BTln\alpha_{H^+} \quad (17)$$

The pH affects the adsorption energies of reaction intermediates by altering the electric potential. This change in pH leads to shifts in the electric potential on the SHE scale also change with a fixed potential on the reversible hydrogen electrode (RHE) scale, as described by the following equation, where n is the number of electron transfers during reactions, e is electron charge constant

$$U_{RHE} = U_{SHE} - \frac{k_B T ln\alpha_{H^+}}{e} = U_{SHE} + \frac{k_B T ln(10)pH}{e} \quad (18)$$

The electrode potential (*U*) referenced to that of RHE and SHE are given by the arranged original equation, where T= 298 K for SHE and RHE

$$\Delta G(U) = \Delta G_0 - neU = \Delta G_0 - neU_{RHE} = \Delta G_0 - neU_{SHE} - nk_B T ln(10)pH \quad (19)$$

Therefore, overall equation considers potential and pH value compared to reference surface at T= 298 K,

$$\Delta G(U,pH) = \Delta G_0 + neU_{SHE} + k_B T ln\alpha_{H^+}$$

$$= \Delta E + \Delta E_{ZPE} - T \times \Delta S - neU_{SHE} - 0.0592 ln \times pH \quad (20)$$

Edge MNene Model Setup Feasibility

In 2D multilayer structures, the constraints imposed by plane waves and the computational limitations on the number of atoms necessitate determining the minimum number of surfaces and fixed layers required to preserve properties comparable to those of infinitely extended 2D materials. To address this, we constructed supercells with dimensions of 1 × 2 × 5, 1 × 2 × 7, and 1 × 2 × 9 edge slab from the unit cell. Concurrently, we examined the impact of the number of fixed bottom surfaces, number of fixed layers in optimized cell as shown in **Figure S57**. The adsorption energy of H on top of N was defined as follows:

$$E_{ads} = E_{sur+H} - E_{sur} - E_H \quad (21)$$

The calculated nitrogen vacancy formation energies are defined as:

$$E_{vac} = E_{sur-N} - \frac{1}{2} E_{N_2} - E_{sur} \quad (22)$$

The convergence trend of adsorption energies E_{ads} was calculated to obtain the optimal number of layers and necessary fixed layer for maintaining the desired properties during adsorption. Overall, we found 1 × 2 × 9 slab model with fixbot4 setup can maintain the change of adsorption energy within 0.02 eV when we further increase system layers. Therefore, we utilize 1 × 2 × 9 slab

model as our main setup for most of AIMD and energy profile calculations. However, when we consider the $Ti_2N(OH)O$ (OHO_edge_O) MNene model, we further consider the 2 × 2 × 9 slab model due to higher vacancy defect ratio on the edge sites.

Discussion of the MvK mechanisms on various edge terminations

Previous research on the $Ti_2N(OH)O$ [1] suggested the surface to be more stable due to strong H bonding between interlayer -O/OH terminations. These edge-exposed surfaces (**Figure S24a**, supporting information) were shown to be preferable for MvK_{as} over MvK_{dis} and $MvK_{as\text{-}dis}$ without applied potential during the 2nd NH_3 cycle. The hydrogenation of $^*NH_2$ adsorbed in the vacancy site (v_NH_2) is the rate limiting step due to the more stable configuration of v_NH_2 when NH_2 adsorbs on the Ti_{bri} sites. Overall, 3.73 eV are required for total hydrogenation from N to NH_3 and to create a vacancy site on the $Ti_2N(OH)O$ surface. Therefore, even though the MvK_{as} is thermodynamically favorable for all steps in the second NH_3 cycle of NRR, it still requires overcoming a higher energy barrier in pH=7 or it requires a greater applied potential in pH=0 to create a nitrogen vacancy (**Figure S24b-c**, supporting information). While the MvK_{as} mechanism is preferable on the $Ti_2N(OH)O$ edge surface in acidic electrolyte, it is more preferred in neutral electrolyte due to the higher energy needed when creating vacancy sites. Furthermore, the NH_3 desorption energy on the $Ti_2N(OH)O$ edge surface requires 1.98-2.32 eV depending on the adsorption site, which is higher than other possible edge surfaces.

As for the $Ti_2N(OH)_2$ edge surface without applied potential (**Figure S25a**, supporting information), during the 2nd NH_3 cycle, we found MvK_{as}, is preferable over MvK_{dis} and $MvK_{as\text{-}dis}$ because the hydrogenation of v_NH_2 step is rate limiting similar to the $Ti_2N(OH)O$ edge surface. Therefore, during the $MvK_{as\text{-}dis}$ mechanism, if NH_2 could stay on the vacancy sites, the hydrogenation of NH_2 becomes easier than with v_NH_2 adsorbed on Ti_{bridge} sites. Furthermore, even under high nitrogen vacancy ratio ($\theta = 1/2$), creating a nitrogen vacancy on $Ti_2N(OH)_2$ edge surface requires 3.65 eV in vacuum. Moreover, creating a nitrogen vacancy on the $Ti_2N(OH)_2$ edge surface requires overcoming a slightly lower energy barrier compared to on the $Ti_2N(OH)O$ edge surface. On average, 1.216 eV is required for each hydrogenation step in the 1st NH_3 cycle, which is lower than the 1.24 eV per hydrogenation step in the $Ti_2N(OH)O$ case. All hydrogenation steps for the 2nd NH_3 cycle are spontaneous under neutral condition with U=-0.415V vs SHE for $MvK_{as\text{-}dis}$ (**Figure S25b**, supporting information). Furthermore, the overpotential of the 2nd NH_3 cycle only

requires an overpotential of η =0.205 V for $MvK_{as\text{-}dis}$ to overcome the Potential-Determining Step (PDS) **(Figure S25b**, supporting information).

For the Ti_2NO_2 edge surface with no applied potential (**Figure S26a**, supporting information), during the 2nd NH_3 cycle, the surface prefers to spontaneously undergo MvK_{as}. With an overpotential of 0.352 eV, however, both $MvK_{as\text{-}dis}$ and MvK_{dis} become easier (**Figure S26b-c**, supporting information). For configurations with nitrogen vacancies, we observe surface diffusion of oxygen which lowers the free energy but can create steric effects that prevent nitrogen gas from filling the vacancy. Under experimental conditions (pH=7, U=0.415V), all proposed MvK mechanisms can overcome the PDS.

As for the Ti_2NO_2 with -OH edge terminations during the 2nd NH_3 cycle, the MvK_{as} mechanism is spontaneously preferred with a negligible overpotential of 0.008 eV, whereas the overpotential of the $MvK_{as\text{-}dis}$ and MvK_{dis} mechanisms are 0.637eV (**Figure S27**, supporting information). Contrary to Ti_2NO_2, the edge -OH terminations prevent surface dislocation characteristic of oxides. Interestingly, a proton moves from O to N on the surface forming NH during DFT optimization thus stabilizing the surface (**Figure S27** step 4-1, supporting information). This further verified our observation that -OH termination could serve as a hydrogen source during the reaction. Under experimental conditions, all proposed MvK mechanisms can overcome the PDS. Additional edge -OH terminations on Ti_2NO_2 increase the free energy required for vacancy creation by 2.3 eV. Therefore, we found the functionalized edge surface would be more crucial for promoting adsorption energy rather than in their role as interlayer terminations, where hydrogen bonding highly influences the stability of the Ti_2NT_x MNene structure [1].

Overall, by comparing $Ti_2N(OH)O$, $Ti_2N(OH)_2$, Ti_2NO_2, and Ti_2NO_2 with edge -OH terminations altogether (**Figure S24-S27**, supporting information), we find MvK_{as} is the most favorable mechanism for all cases. However, once the N≡N bond (from vac_NNH_2) breaks, if there is no proton to form NH_3, the -NH_2 may diffuse to the Ti_{bridge} site and further become v_NH_2 undergoing the $MvK_{as\text{-}dis}$ path. Therefore, the MvK_{as} and $MvK_{as\text{-}dis}$ mechanisms are likely to happen concurrently. Furthermore, among the four edge MNene surfaces, $Ti_2N(OH)O$ possesses the highest energy barrier to form a nitrogen vacancy surface with 2.74 eV, whereas Ti_2NO_2 only requires 1.66 eV due to the oxygen on the edge surface dislocating to form more Ti-O moieties, which is prevented on the fully –OH terminated material. Furthermore, comparing the edge surface of Ti_2NO_2 and Ti_2NO_2 with -OH terminations, we found that an even distribution of -OH

terminations on the edge sites of Ti_2NO_2 might increase the energy barrier to form a nitrogen vacancy on the surface, thus limiting the MvK mechanism. Therefore, by controlling the pH value of the electrolyte, protonation of oxygen on the edge surface can be modified, further promoting the MvK mechanism by decreasing the energy to form a nitrogen vacancy. However, in previous AIMD results without -OH terminations, no sign of nitrogen vacancy refilling is observed on Ti_2NO_2, whereas Ti_2NO_2 with -OH terminations allow spontaneous protonation to form -N_2H_2 on vacancy sites. Therefore, a tradeoff exists between formation of nitrogen vacancies and hydrogenation of replenished vacancies based on the ratio of –OH edge terminations on the Ti_2NO_2 surface.

Validation of Mars-van Krevelen Mechanism on vacancy filling and NH_3 desorption

This study primarily aimed to investigate the refilling of the vacancy and the subsequent NH_3 desorption in the context of the Mars-van Krevelen mechanism. Our investigation focused on four key steps and factors, which are outlined as follows:

1. The step evaluates the cases of NH_3 removal after the vacancy site has been refilled with N, aiming to gain insights into the efficiency of NH_3 desorption (* +NH_3) on various terminated Ti_2NT_x surfaces. The results are shown in **Figure S45, S47, S59** and summarize in **Figure S38**.
2. The step involves examining whether the product NH_3 from MvK or NRR will filling back to nearby vacancy sites after of one surface N atom transforms into NH_3 (* Vac +NH_3) on various terminated Ti_2NT_x surfaces. The results are shown in **Figure S46, S60, S61** and summarized in **Figure S38**.
3. The step explores the replenishment of Nitrogen vacancy sites with adsorbed N_2 molecules in the presence of nearby NH_3 before undergoing desorption (*Vac +N_2 +NH_3) on various terminated Ti_2NT_x surfaces with different electrolytes. The results of acidic and neutral electrolytes are shown in **Figure S28, S32, S40, S41-S43,** summarized in **Figure S44** and result of water are shown in **Figure S22, S48, S62,** summarized in **Figure S38**.
4. The step explores the replenishment of the surface with adsorbed N_2 molecules without NH_3 that attached nearby and MvK mechanism (* Vac +N_2) in 2×2×9 supercell on various terminated Ti_2NT_x surfaces with different electrolytes. The detailed results of acidic and neutral electrolytes are shown in **Figure 4a-l, S30, S33, S35-S37,** summarized in **Figure 5** and result of water are shown in **Figure S23, S29, S34, S63** and summarized in **Figure S39**.

From analysis above AIMD process, we focus on three main local enviroment factors that determine the feasiabiltiy of refilling the vacancy, including 1. Timing of hydrogenation & Hydrogenation Number of N_2, 2. -OH termination importance on edge surface & concentration of H source 3. nearby NH_3 adsorption

Presence of Nearby NH_3 Adsorption

Here, we also conduct a comparative analysis on the influence of acidic electrolytes (**Figure 4a-f, S28, S30, S35, S36, S40 and S41,** supporting information) versus neutral electrolytes for MvK, as shown in (**Figure 4g-l, S31, S32, S33, S37, S42, S43,** supporting information) and summarized in **Figure 5 and Figure S44**. Interestingly, we observe that a higher hydronium concentration accelerates the reduction of N_2. However, excessively rapid hydrogenation prior to vacancy filling may hinder vacancy replenishment, leading to migration towards nearby Ti bridge sites, as discussed earlier. Consequently, the control of proton concentration near the surface becomes crucial, significantly affecting the replenishment of vacancies. Additionally, besides water splitting and hydronium acting as proton sources, we observe the SO_4^{2-} ions could stabilize and balance hydronium through facilitating the transfer of H^+ from solution, termination groups, or water splitting to surface nitrogen atoms. In some cases, SO_4^{2-} and HSO_4^- aid in proton transfer from water or nearby -OH terminations to N_2.

Several key observations emerged from AIMD studies on NH_3 desorption and filling during nitrogen vacancy tests. We found that NH_3 desorption and hopping occurs on the $Ti_2N(OH)O$ (OHO_edge_O) surface regardless of the presence of vacancies (**Figure S45, S46**, supporting information). Interestingly, neither NH_3 nor H_2O embed the defect in Ti_2NT_x for all studied surfaces, instead, they shift closer to nearby interlayers when N_2 or N_2H is present (**Figure S47, S48, S28, S32,** and **S41**, supporting information). It was found that NH_3 desorption is hindered from the replenishment of N_2. Furthermore, instead of desorbing, the NH_3 could serve as a proton source to assist the hydrogenation of nearby NH or NH_2. It is observed that NH_3 deprotonates to NH_2 and transfers the H^+ to nearby NNH, thus forming NNH_2. This NNH_2 starts to fill the vacancy, breaking the N-N bond to form NH_2, which then reforms NH_3 near the $Ti_2N(OH)_2$ interlayer (**Figure S28**, supporting information). However, through comparison of (O_edge_OH) and (OHO_edge_O) surfaces with the presence of NH_3, N_2 tends to undergo

traditional NRR pathways, thus forming N_2H_2 or N_2H at the Ti bridge site rather than replenishing the vacancy site (**Figure S38**). Conversely, in the absence of NH_3 on the surface, N_2 and N_2H filling becomes more facile (**Figure S39)**. These findings suggest a complex interplay between NH_3, N_2, and N_2H in the desorption and vacancy filling processes.

Supplementary Figures

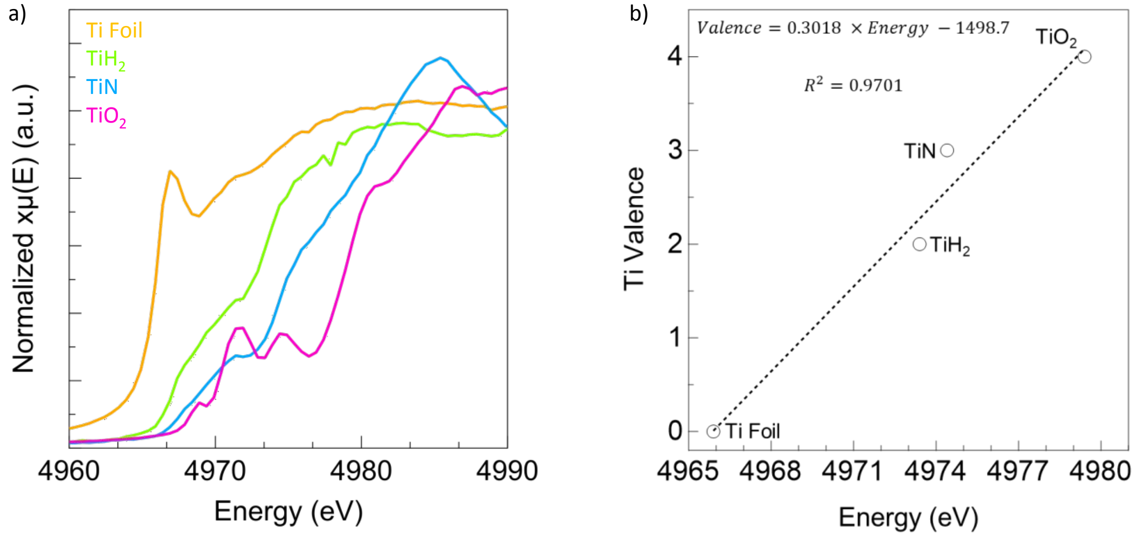

Figure S1. a) XANES region of the normalized Ti K-edge XAS spectra for Ti foil (orange), TiH_2 (green), TiN (blue), and TiO_2 (magenta) materials to be used as reference materials. b) Edge position determined from XANES spectra of the Ti reference materials as a function of Ti-valency.

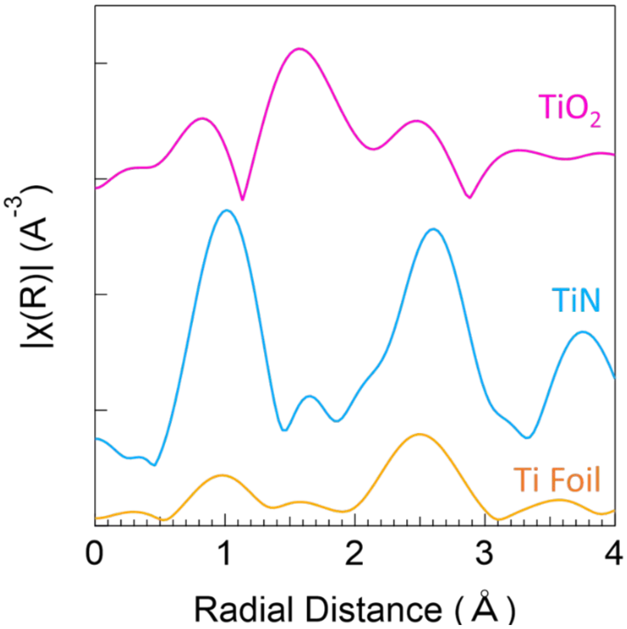

Figure S2. Fourier transform of the normalized EXAFS spectra for Ti foil (orange), TiN (blue), and TiO_2 (purple). A k-weight of 2 was used.

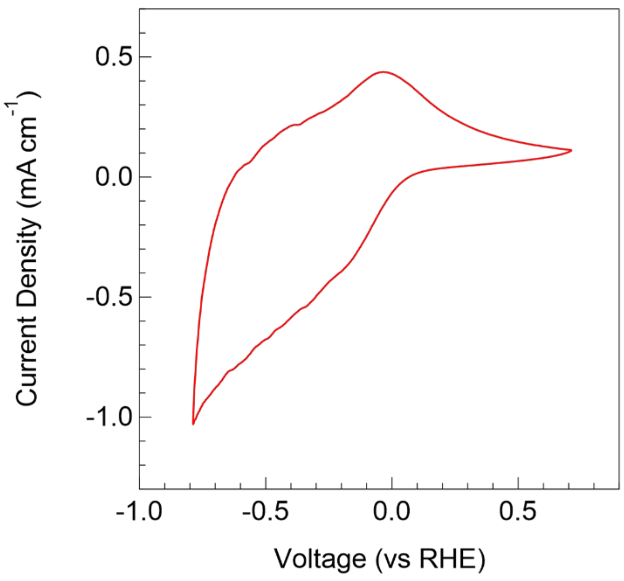

Figure S3. Cyclic voltammogram of Ti_2NT_x MNene catalyst in N_2-saturated 0.1M Na_2SO_4 electrolyte. CV was conducted at a scan rate of 50 mV/s over a 1.5V window.

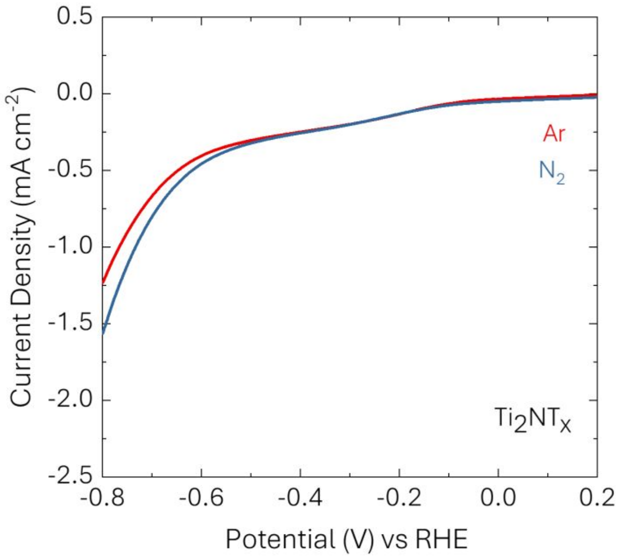

Figure S4. Linear sweep voltammetry of Ti_2NT_x MNene catalyst in Ar and N_2-saturated 0.1M Na_2SO_4 electrolyte. LSV was conducted at a scan rate of 5 mV/s.

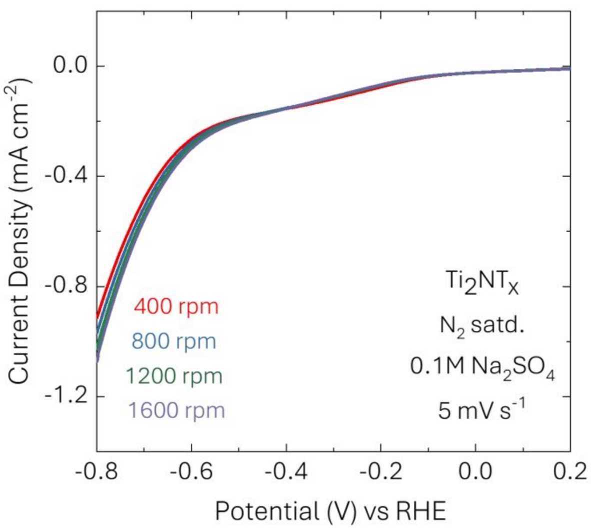

Figure S5. Linear sweep voltammetry of Ti_2NT_x MNene catalyst in N_2-saturated 0.1M Na_2SO_4 electrolyte carried out in rotating disk electrode at different rpm. LSV was conducted at a scan rate of 5 mV/s.

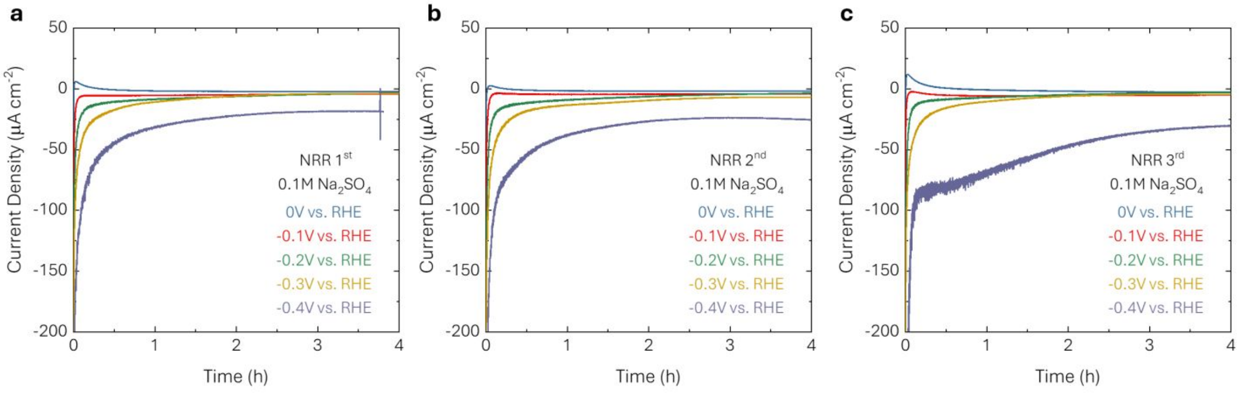

Figure S6. I-*t* curves for 4 h NRR chronoamperometry experiments in N_2-saturated 0.1M Na_2SO_4 electrolyte at varying potentials.

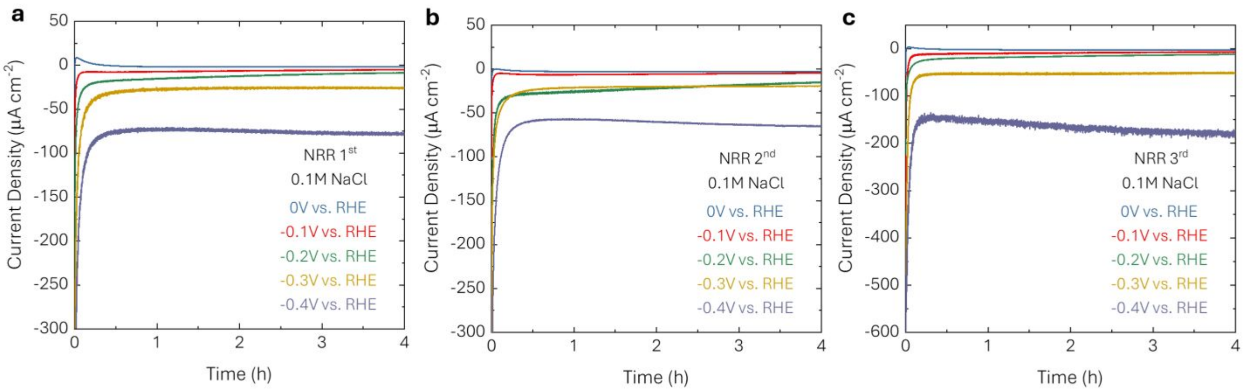

Figure S7. I-*t* curves for 4 h NRR chronoamperometry experiments in N_2-saturated 0.1M NaCl electrolyte at varying potentials.

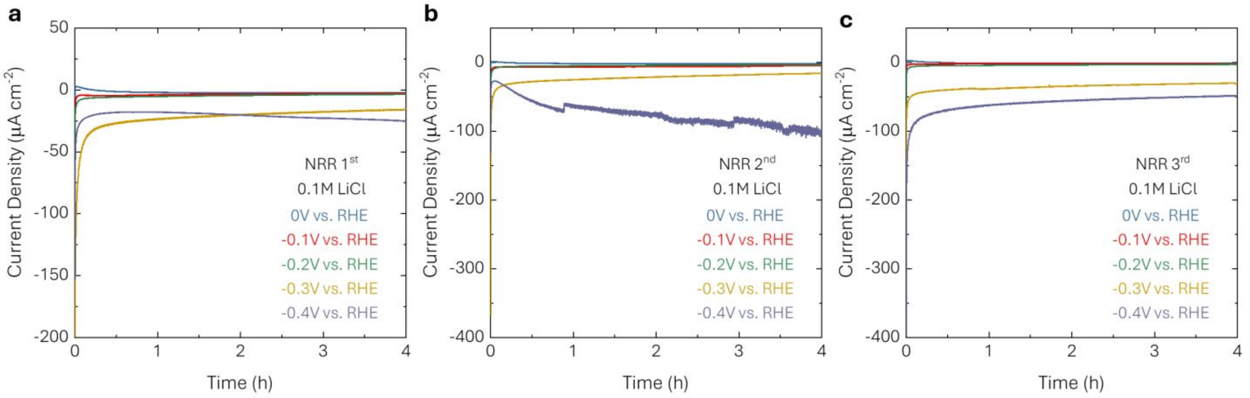

Figure S8. I-*t* curves for 4 h NRR chronoamperometry experiments in N_2-saturated 0.1M LiCl electrolyte at varying potentials.

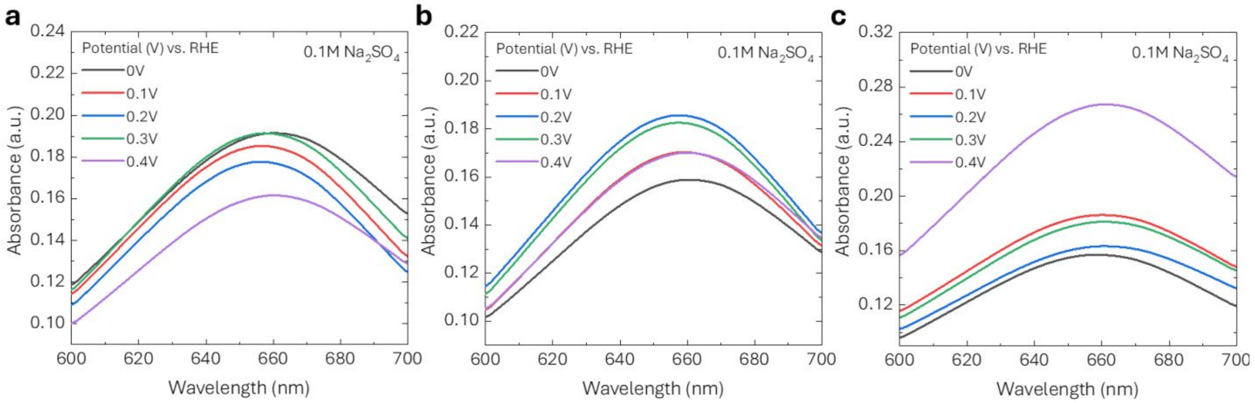

Figure S9. UV-Vis absorption spectra after 4 h NRR chronoamperometry experiments in N_2-saturated 0.1M Na_2SO_4 electrolyte at varying potentials.

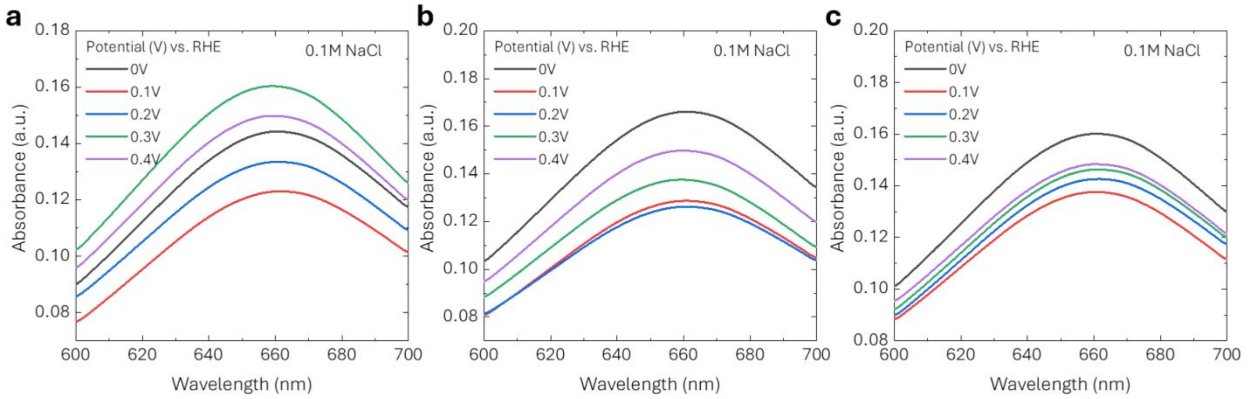

Figure S10. UV-Vis absorption spectra after 4 h NRR chronoamperometry experiments in N_2-saturated 0.1M NaCl electrolyte at varying potentials.

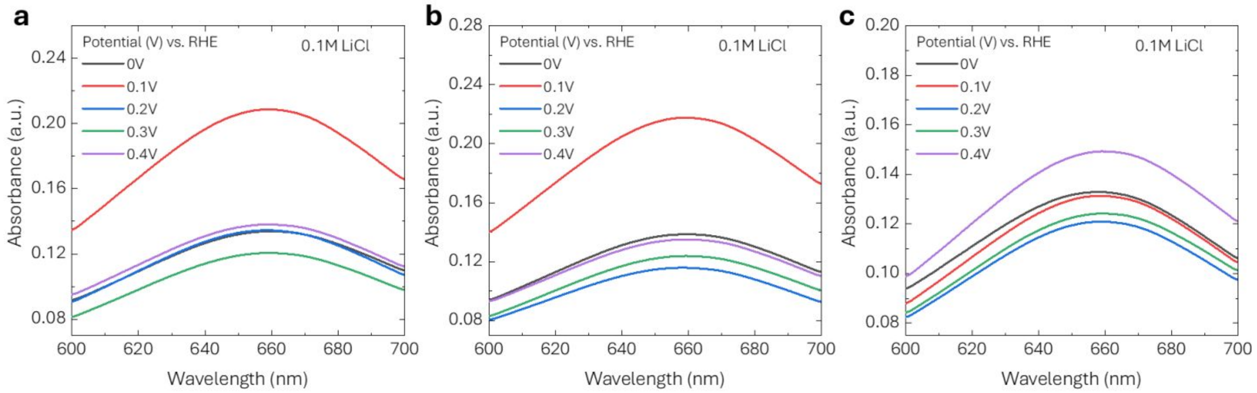

Figure S11. UV-Vis absorption spectra after 4 h NRR chronoamperometry experiments in N_2-saturated 0.1M LiCl electrolyte at varying potentials.

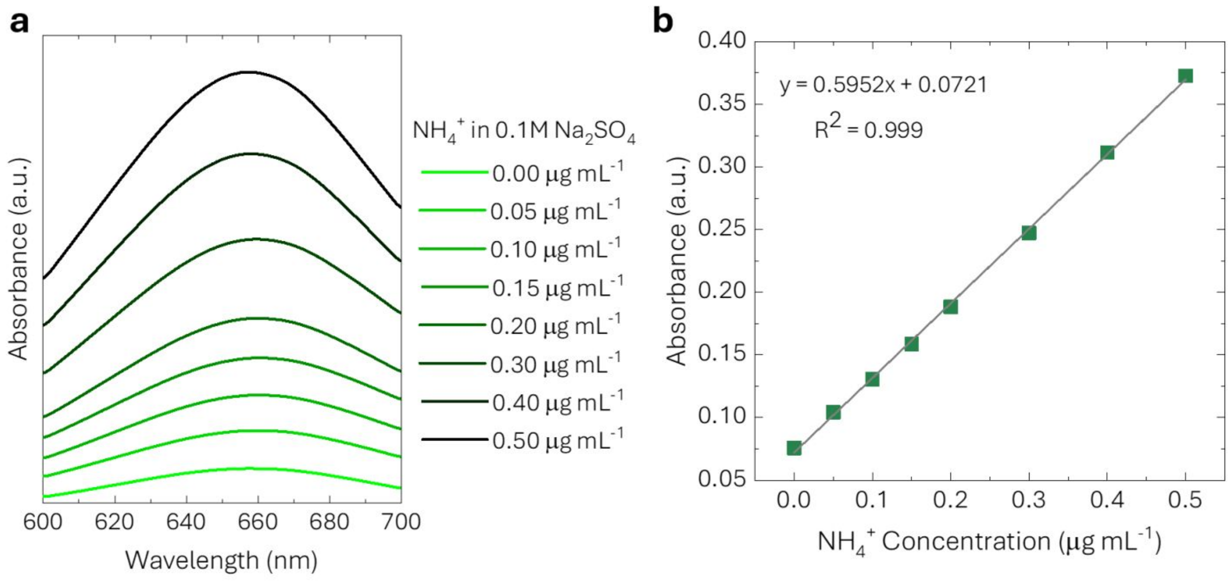

Figure S12. Berthelot method of NH_3 quantification in 0.1M Na_2SO_4. **a)** UV-Vis absorption spectra of various concentrations of standard NH_4^+. **b)** Corresponding calibration curve.

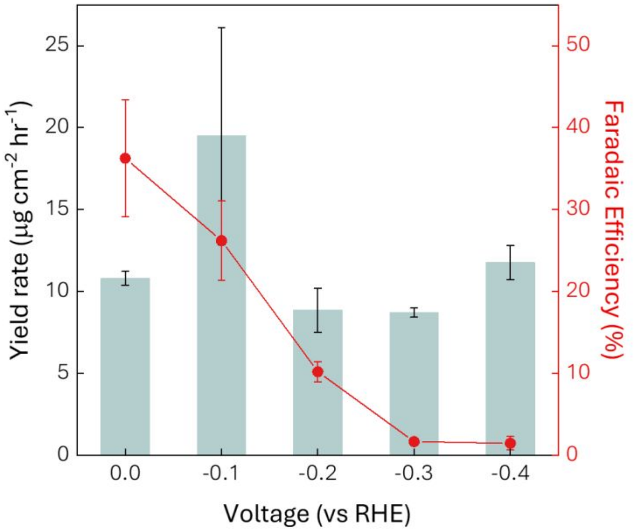

Figure S13. NH_3 yield and Faradaic efficiency of Ti_2NT_x MNene in N_2-saturated 0.1M LiCl electrolyte at varying potentials after 4 h chronoamperometry experiments. All bars correspond to yield values (read to the left) while lines with markers correspond to FE values (read to the right).

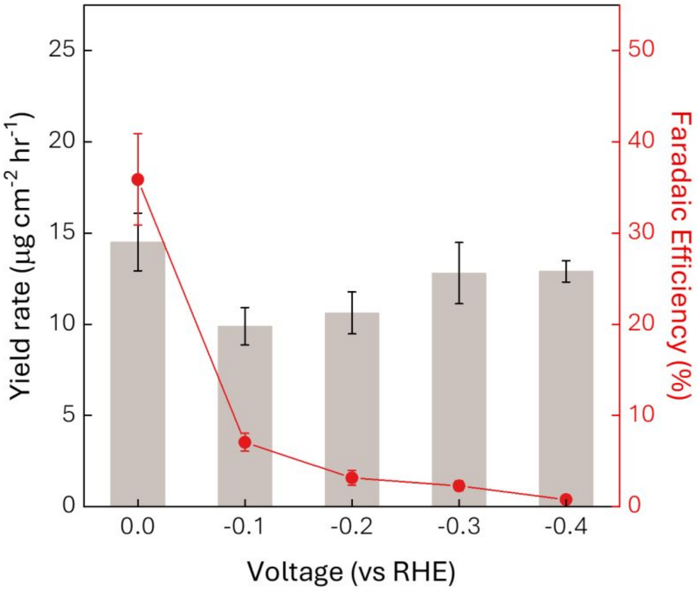

Figure S14. NH_3 yield and Faradaic efficiency of Ti_2NT_x MNene in N_2-saturated 0.1M NaCl electrolyte at varying potentials after 4 h chronoamperometry experiments. All bars correspond to yield values (read to the left) while lines with markers correspond to FE values (read to the right).

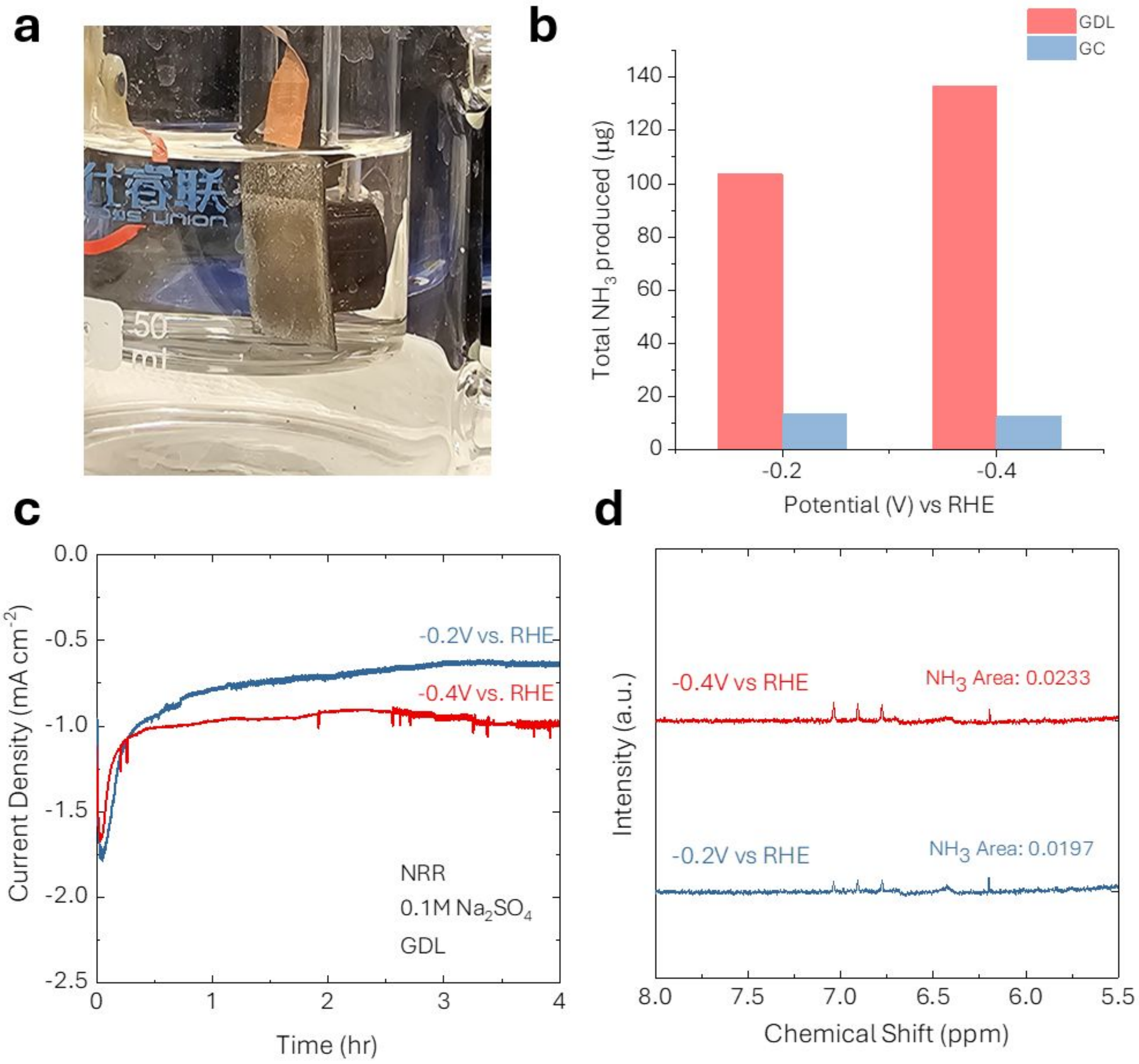

Figure S15. NRR experiment using a Ti_2NT_x spray-coated gas diffusion layer (GDL) with an active area of 4 cm^2. To address the low solubility of N_2 in aqueous electrolyte, N_2 gas was supplied through the backside of the GDL by attaching a custom lid, maintaining a flow rate of 2 mL/min to ensure continuous N_2 availability at the catalyst surface during NRR. **a)** Photograph of the GDL setup showing the attached lid on the backside. **b)** Comparison of total NH_3 yield of the Ti_2NT_x MNene in 0.1 M Na_2SO_4 electrolyte using GDL and GC setup (4 cm^2 vs 0.192 cm^2 electrode geometric area) **c)** Corresponding chronoamperometry curves in GDL setup. **d)** ^1H NMR analysis of the electrolyte after the experiments for NH_3 quantification. Overall, this study shows increasing total NH_3 production with increasing electrode area indicating that produced NH_3 is from electrochemical NRR.

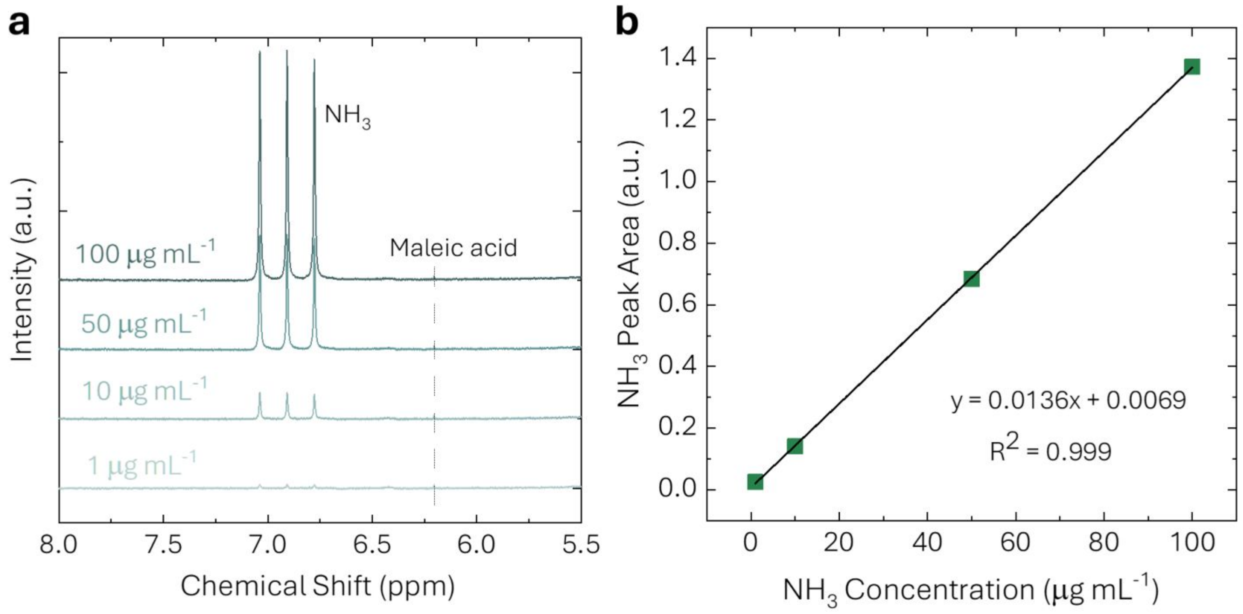

Figure S16. a) ^{1}H NMR spectra of standard NH_4^+ solutions at varying concentrations prepared in 0.1M Na_2SO_4 electrolyte. **b)** Corresponding calibration curve showing the linear fit of the integrated NH_3 peak area versus NH_4^+ concentration. For sample preparation, 600 μL of analyte was mixed with 100 μL of 100 mM maleic acid, 50 μL 1M H_2SO_4 and 50 μL of DMSO-d_6.

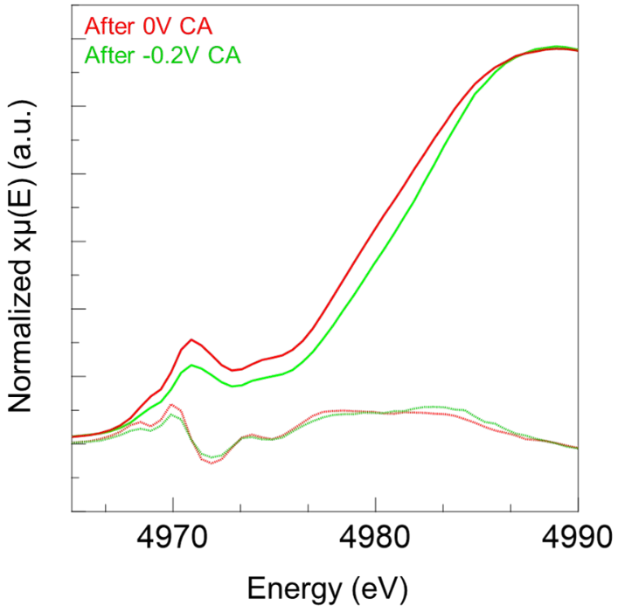

Figure S17. XANES region of the normalized Ti K-edge XAS spectra for Ti_2NT_x catalyst after NRR electrocatalysis performed at 0V vs RHE (red) and -0.2V vs RHE (green).

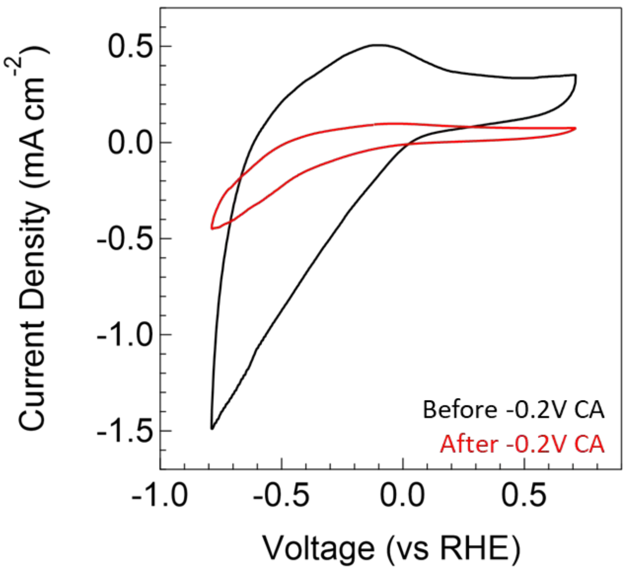

Figure S18. Cyclic voltammograms before (black) and after (red) 4 h chronoamperometry experiments at -0.2V vs. RHE. CV experiments were conducted at scan rate of 50 mV s^{-1}.

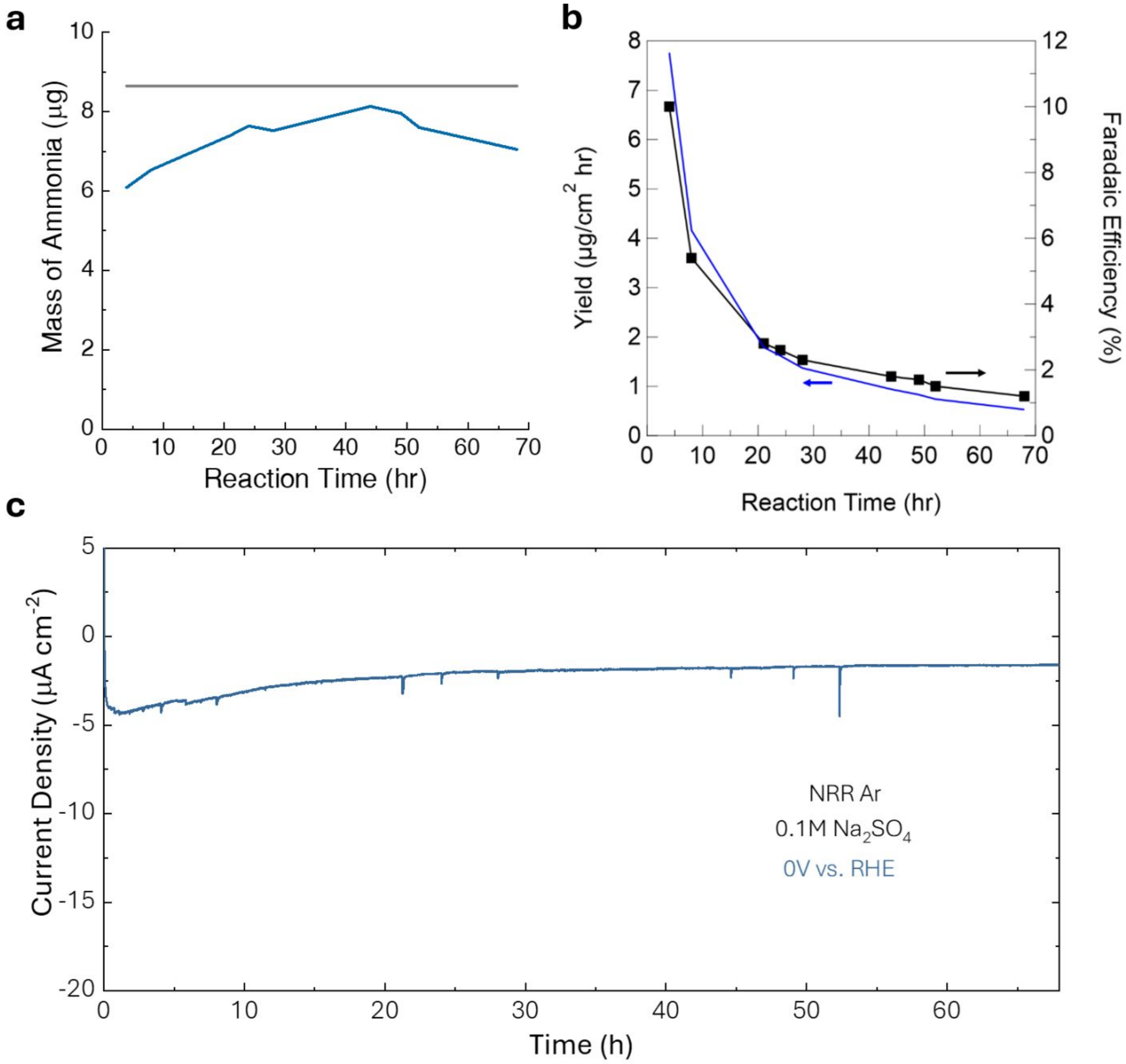

Figure S19. a) Experimental (blue) amount of NH_3 produced compared against the maximum theoretical amount (grey) based on complete lattice N conversion during a 68-hour period chronoamperometry under an inert Ar atmosphere. **b)** Faradaic efficiency (black) and yield (blue) as a function of time during a 68-hour period chronoamperometry under an inert Ar atmosphere. Faradaic efficiency is read to the right and yield to the left. **c)** Corresponding chronoamperometry curve for 68 h in 0.1M Na_2SO_4 electrolyte.

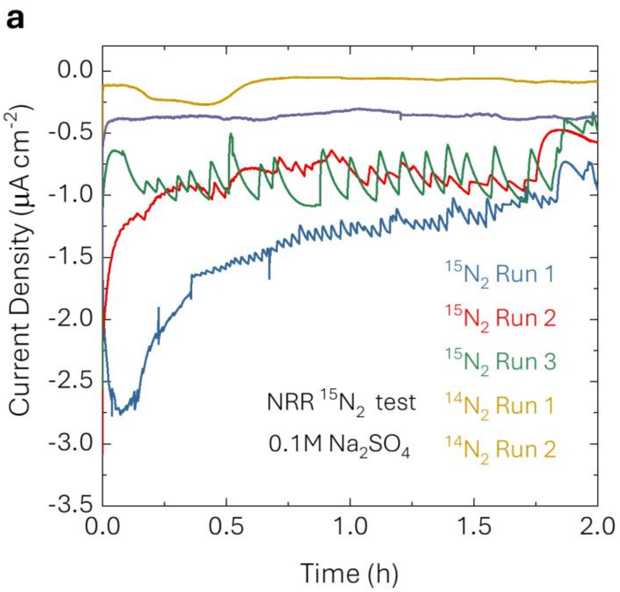

Figure S20. a) Chronoamperometry curves for isotopic exchange experiments for **Figure 3** in main manuscript. **b)** Peak area ration of $^{15}NH_3$ /$^{14}NH_3$ plotted as function of NRR experiment time. First three data points were obtained from **Figure 3**, whereas the last data point was calculated from a separate 24 h long $^{15}N_2$ NRR experiment.

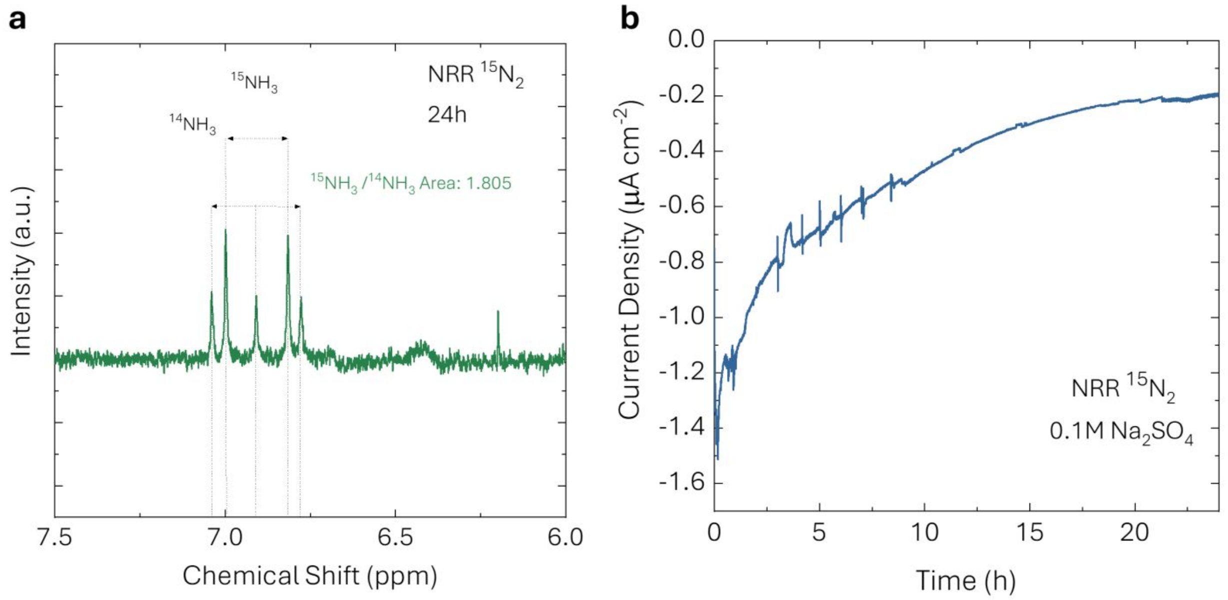

Figure S21. a) 1H-NMR results from straight 24 h NRR carried out using $^{15}N_2$ isotope. **b)** Corresponding chronoamperometry curve.

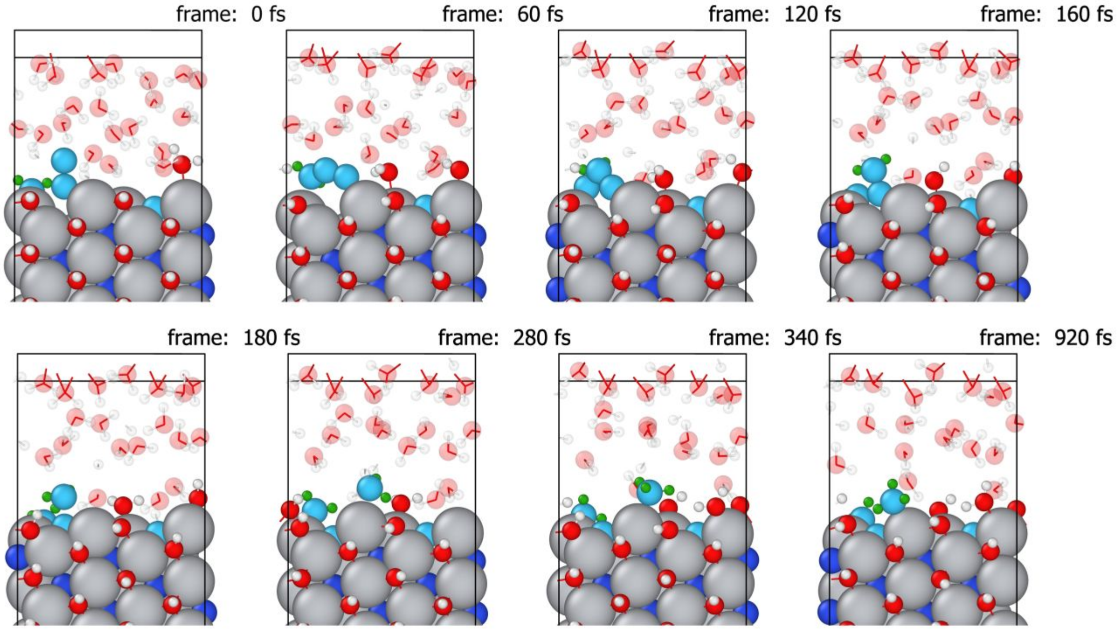

Figure S22. Snapshots from AIMD simulations of NH_3 desorption test and N_2 MvK NRR pathway test on $Ti_2N(OH)_2$ model surface in water with vacancy ($\theta = \frac{1}{2}$). Where cyan, blue, white, green, grey, and red represent nitrogen atoms above the first layer, nitrogen atoms below the first layer, hydrogen, and hydrogen adsorbed on nitrogen, titanium, and oxygen, respectively.

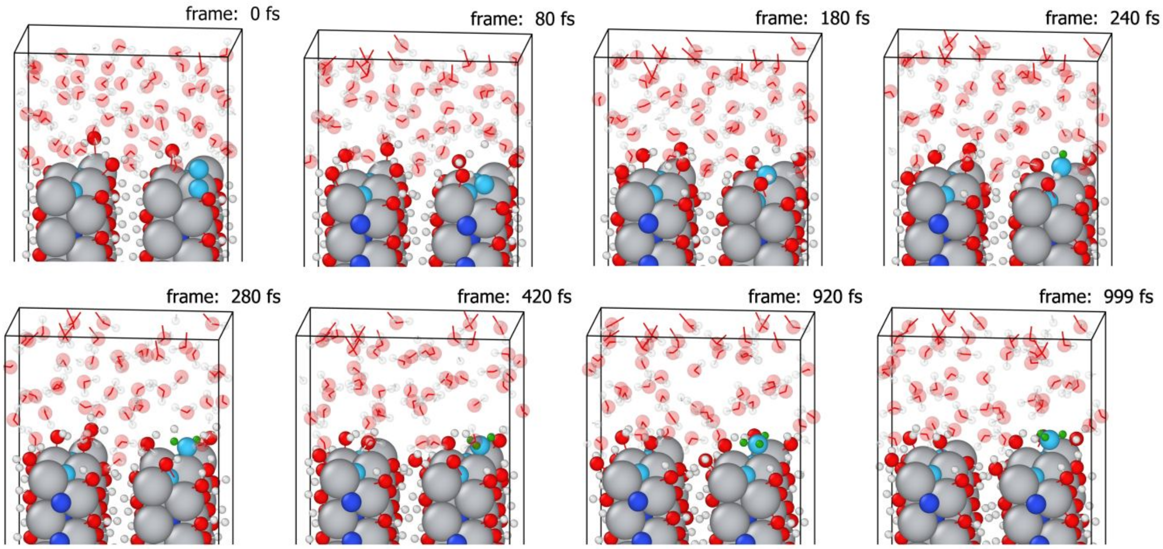

Figure S23. Snapshots from AIMD simulations of N_2 undergo MvK pathway on $Ti_2N(OH)_2$ bilayer model surface in water with nitrogen vacancy ($\theta = \frac{1}{4}$). Where cyan, blue, white, green, grey, and red represent nitrogen atoms above the first layer, nitrogen atoms below the first layer, hydrogen, and hydrogen adsorbed on nitrogen, titanium, and oxygen, respectively.

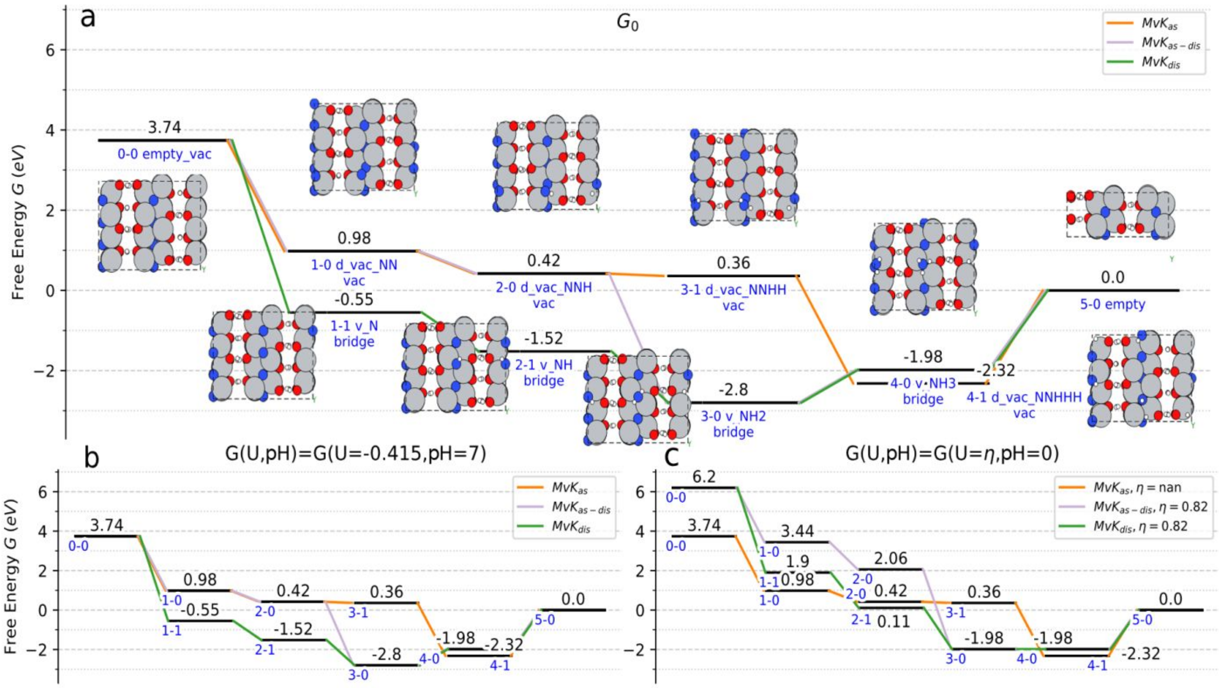

Figure S24. Free energy profile for NRR through different MvK path on $Ti_2N(OH)O$ surface with Nitrogen vacancy $*_{vac}$. Energy profile undergoes associative MvK mechanism, dissociative MvK mechanism and associative-dissociative mechanism are shown as MvK_{as} in orange, $MvK_{as\text{-}dis}$ in purple and MvK_{dis} in green, which are considering different applied bias potentials and pH values. **a)** The energy profiles with pH = 0 at 298 K when there is no applied bias, U = 0 V. **b)** The energy profiles with pH = 7 at 298 K with U = -0.415 applied bias matches experimental best performance potential bias with 1.25M Na_2SO_4 and -0.612 V vs Ag/AgCl (0 V vs RHE). **c)** The energy profiles with pH = 0 at 298 K and with the applied bias potentials that overcome the PDS are presented with each pathway. After applied overpotential η, all the elementary steps yield either no change or a decrease in free energy. The free energy in this figure is normalized and divided by two due to two hydrogenations happening between each step. Overall, $Ti_2N(OH)O$ likely undergoes MvK_{as} without applied potential bias.

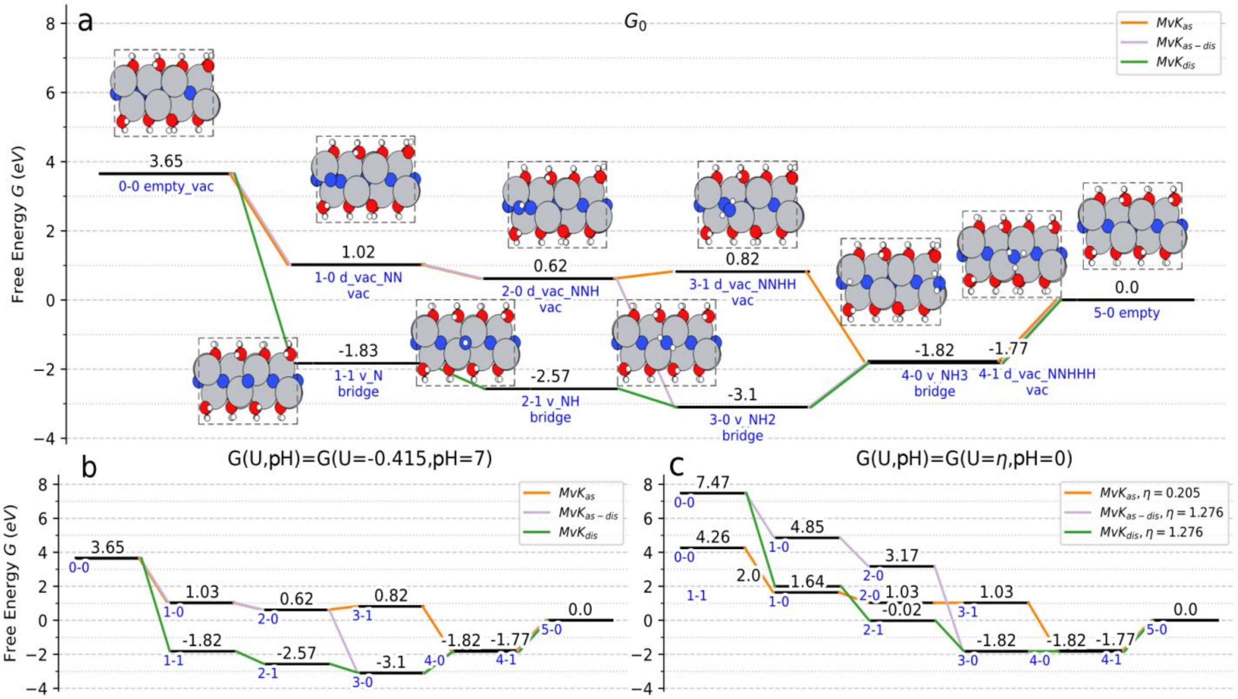

Figure S25. Free energy profile for NRR through different MvK path on $Ti_2N(OH)_2$ surface with Nitrogen vacancy $*_{vac}$. Energy profile undergoes associative MvK mechanism, dissociative MvK mechanism and associative-dissociative mechanism are shown as MvK_{as} in orange, $MvK_{as\text{-}dis}$ in purple and MvK_{dis} in green, which are considered under different applied bias potentials and pH value. **a)** The energy profiles with pH = 0 at 298 K when there is no applied bias, U = 0 V. **b)** The energy profiles with pH = 7 at 298 K with U = -0.415 applied bias matches experimental best performance potential bias with 1.25M Na_2SO_4 and -0.612 V vs Ag/AgCl (0 V vs RHE). **c)** The energy profiles with pH = 0 at 298 K and with the applied bias potentials that overcome the PDS are presented with each pathway. After applied overpotential η, all the elementary steps yields either no change or a decrease in free energy. Overall, $Ti_2N(OH)_2$ prefers to undergo MvK_{as} mechanism.

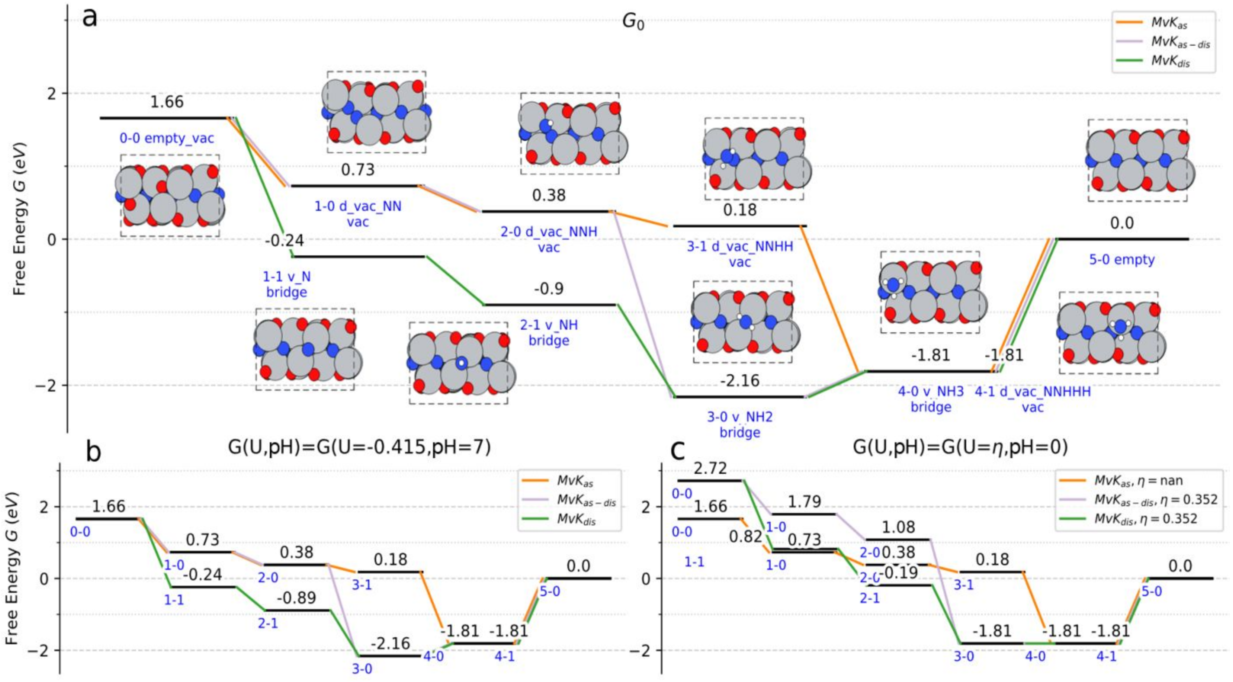

Figure S26. Free energy profile for NRR through different MvK path on Ti_2NO_2 surface with Nitrogen vacancy $*_{vac}$. Energy profile undergoes associative MvK mechanism, dissociative MvK mechanism and associative-dissociative mechanism are shown as MvK_{as} in orange, $MvK_{as\text{-}dis}$ in purple and MvK_{dis} in green, which are considered under different applied bias potentials and pH value. **a)** The energy profiles with pH = 0 at 298 K when there is no applied bias, U = 0 V. **b)** The energy profiles with pH = 7 at 298 K with U = -0.415 applied bias matches experimental best performance potential bias with 1.25M Na_2SO_4 and -0.612 V vs Ag/AgCl (0 V vs RHE). **c)** The energy profiles with pH = 0 at 298 K and with the applied bias potentials that overcome the PDS are presented with each pathway. After applied overpotential η, all the elementary steps yields either no change or a decrease in free energy. Overall, $Ti_2N(O)_2$ surface still prefers the MvK_{as} mechanism but undergoes $MvK_{as\text{-}dis}$ and MvK_{dis} more easily compared to $Ti_2N(OH)O$ and $Ti_2N(OH)_2$ surface.

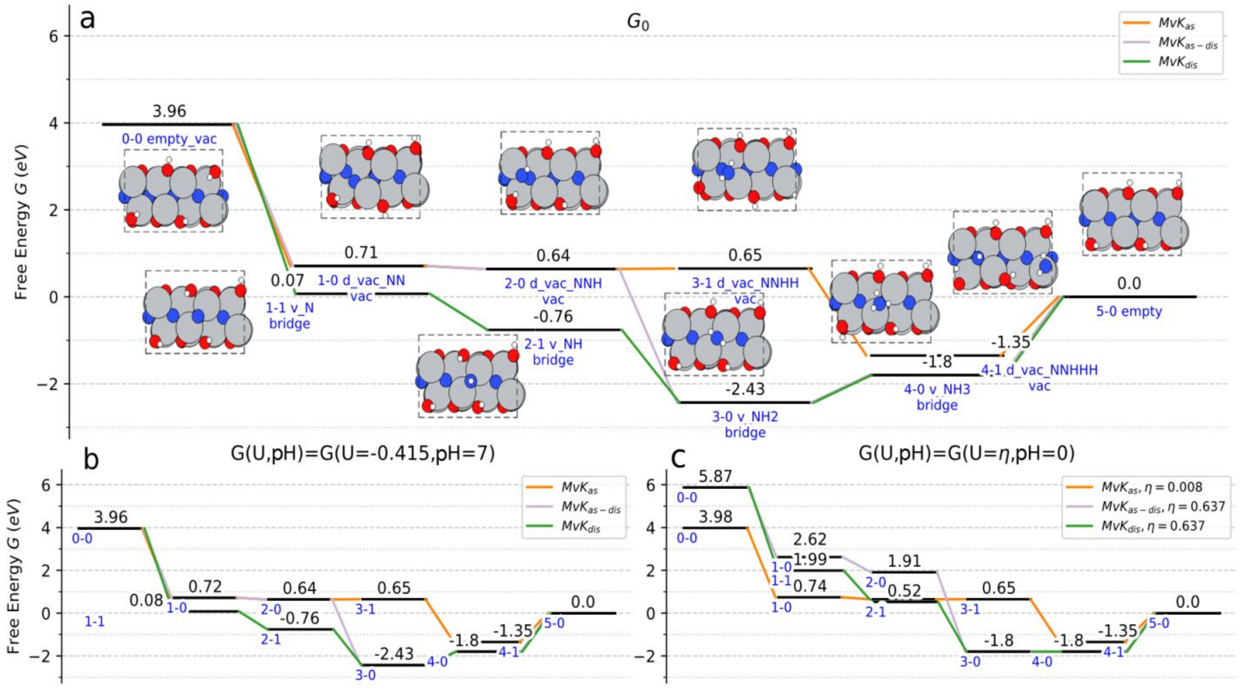

Figure S27. Free energy profile for NRR through different MvK path on Ti_2NO_2 with edge -OH termination surface with Nitrogen vacancy $*_{vac}$. Energy profile undergoes associative MvK mechanism, dissociative MvK mechanism and associative-dissociative mechanism are shown as MvK_{as} in orange, $MvK_{as\text{-}dis}$ in purple and MvK_{dis} in green, which are considered under different applied bias potentials and pH value. **a)** The energy profiles with pH = 0 at 298 K when there is no applied bias, U = 0 V. **b)** The energy profiles with pH = 7 at 298 K with U = -0.415 applied bias matches experimental best performance potential bias with 1.25M Na_2SO_4 and -0.612 V vs Ag/AgCl (0 V vs RHE). **c)** The energy profiles with pH = 0 at 298 K and with the applied bias potentials that overcome the PDS are presented with each pathway. After applied overpotential η, all the elementary steps yields either no change or a decrease in free energy. Overall, Ti_2NO_2 with edge -OH surface still prefer to undergo MvK_{as} mechanism. Interestingly, during the third hydrogenation step, we observed that hydrogen exhibits a preference for adsorption near the top nitrogen atom sites rather than further adsorbing onto the NH_2 molecules on titanium sites. This observation suggests that different hydrogenation steps may compete with one another.

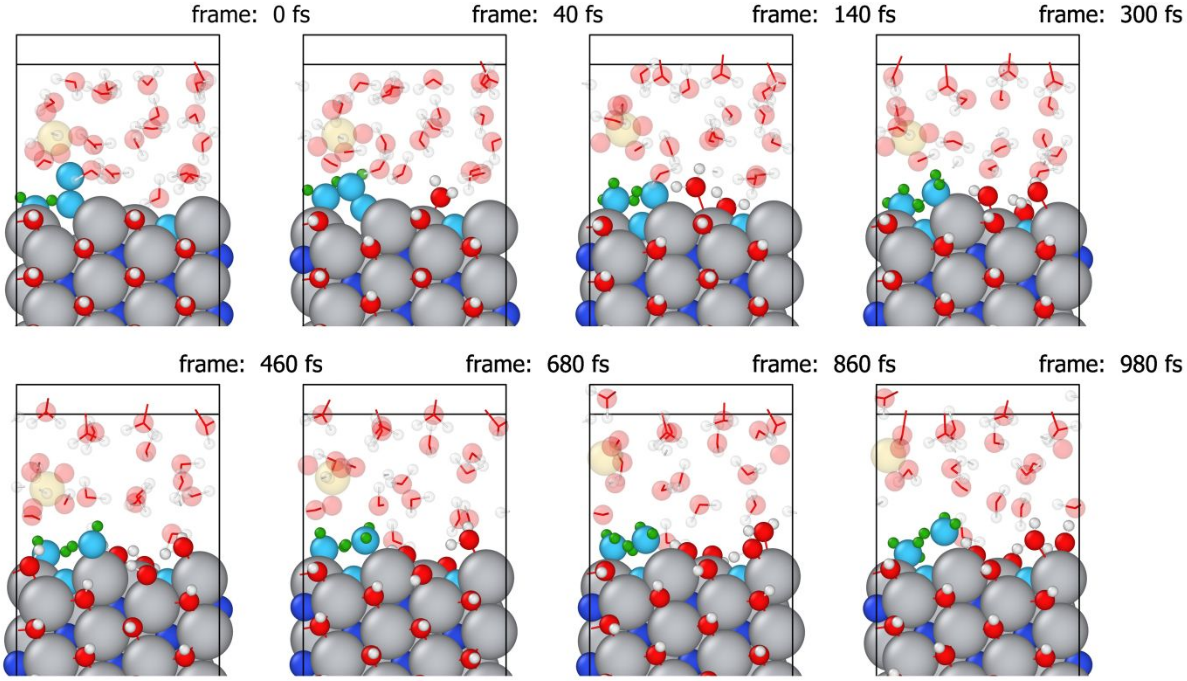

Figure S28. Snapshots from AIMD simulations of NH_3 desorption test and N_2 MvK NRR pathway test on $Ti_2N(OH)_2$ model surface in 2.5 M H_2SO_4 with vacancy ($\theta = \frac{1}{2}$). Where cyan, blue, white, green, grey, red, and yellow represent nitrogen atoms above the first layer, nitrogen atoms below the first layer, hydrogen, and hydrogen adsorbed on nitrogen, titanium, oxygen, and sulfur, respectively.

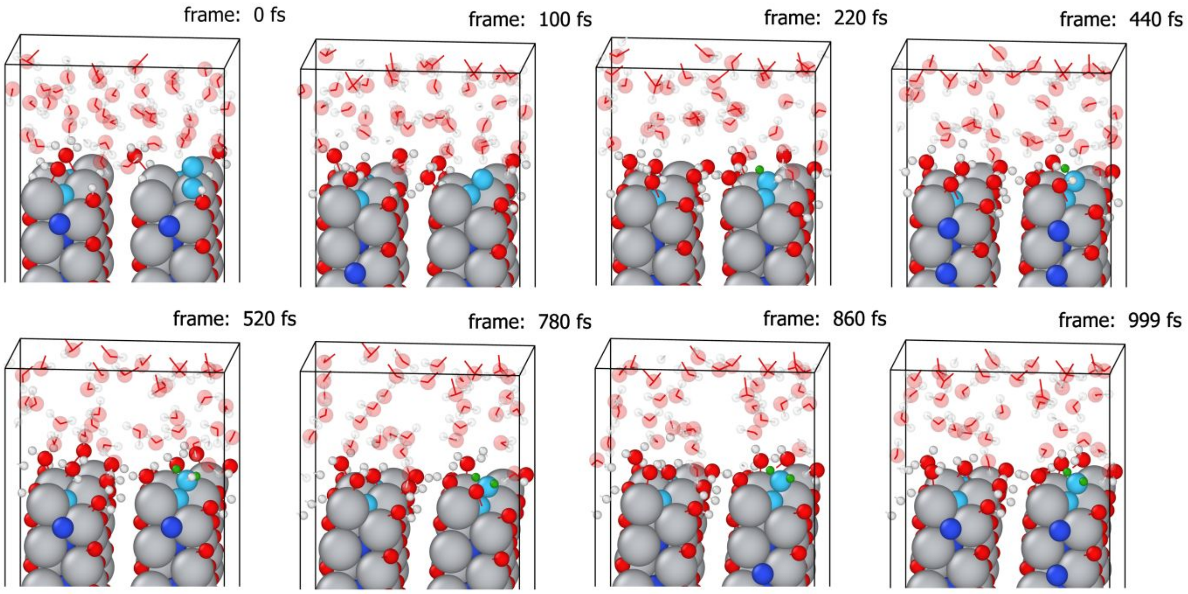

Figure S29. Snapshots from AIMD simulations of N_2 undergo MvK pathway on $Ti_2N(O)_2$ bilayer model surface with edge-facet –OH termination in water with nitrogen vacancy ($\theta = \frac{1}{4}$). Where cyan, blue, white, green, grey, and red represent nitrogen atoms above the first layer, nitrogen atoms below the first layer, hydrogen, and hydrogen adsorbed on nitrogen, titanium, and oxygen, respectively.

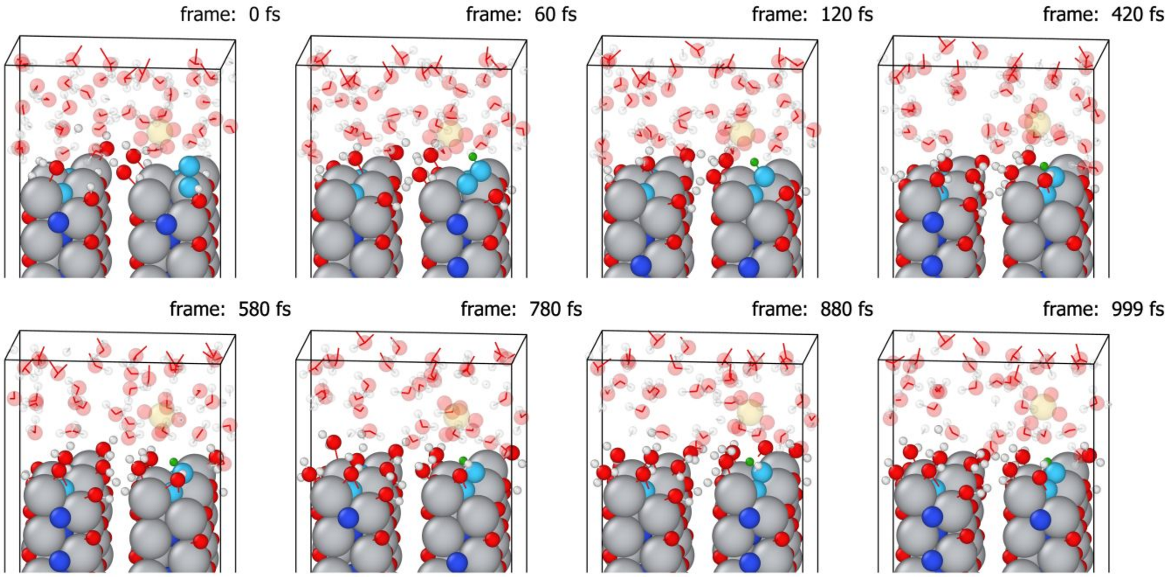

Figure S30. Snapshots from AIMD simulations of N_2 undergo MvK pathway on $Ti_2N(O)_2$ bilayer model surface with edge-facet –OH termination in 1.25 M H_2SO_4 solution with nitrogen vacancy ($\theta = \frac{1}{4}$). Where cyan, blue, white, green, grey, red, and yellow represent nitrogen atoms above the first layer, nitrogen atoms below the first layer, hydrogen, and hydrogen adsorbed on nitrogen, titanium, oxygen, and sulfur, respectively.

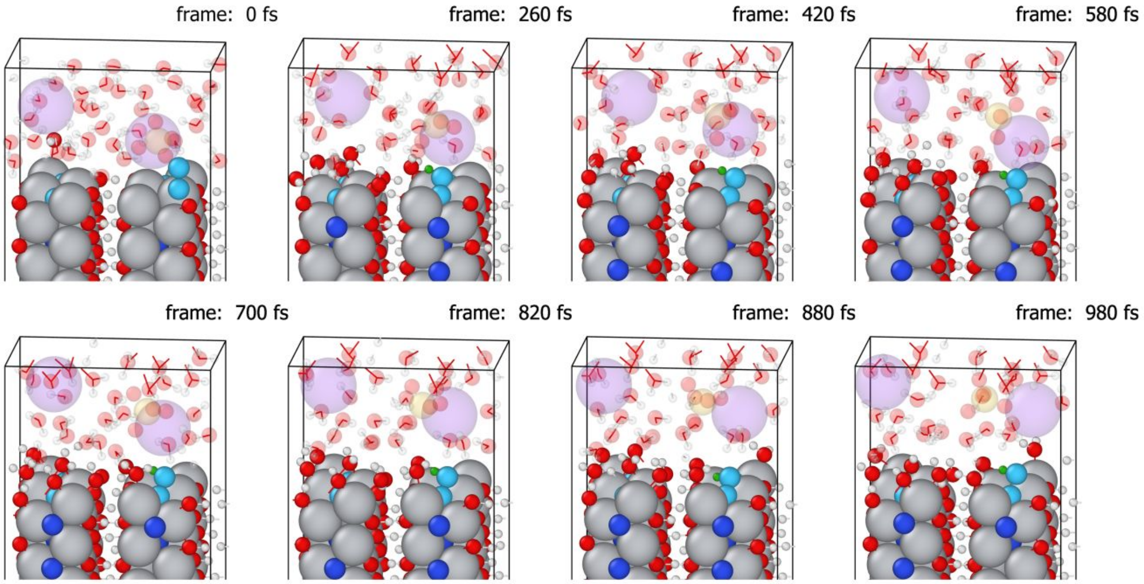

Figure S31. Snapshots from AIMD simulations of N_2 undergo MvK pathway on $Ti_2N(OH)(O)$ bilayer model surface in 1.25 M Na_2SO_4 with nitrogen vacancy ($\theta = \frac{1}{4}$). Where cyan, blue, white, green, grey, red, purple, and yellow represent nitrogen atoms above the first layer, nitrogen atoms below the first layer, hydrogen, and hydrogen adsorbed on nitrogen, titanium, oxygen, sodium, and sulfur, respectively.

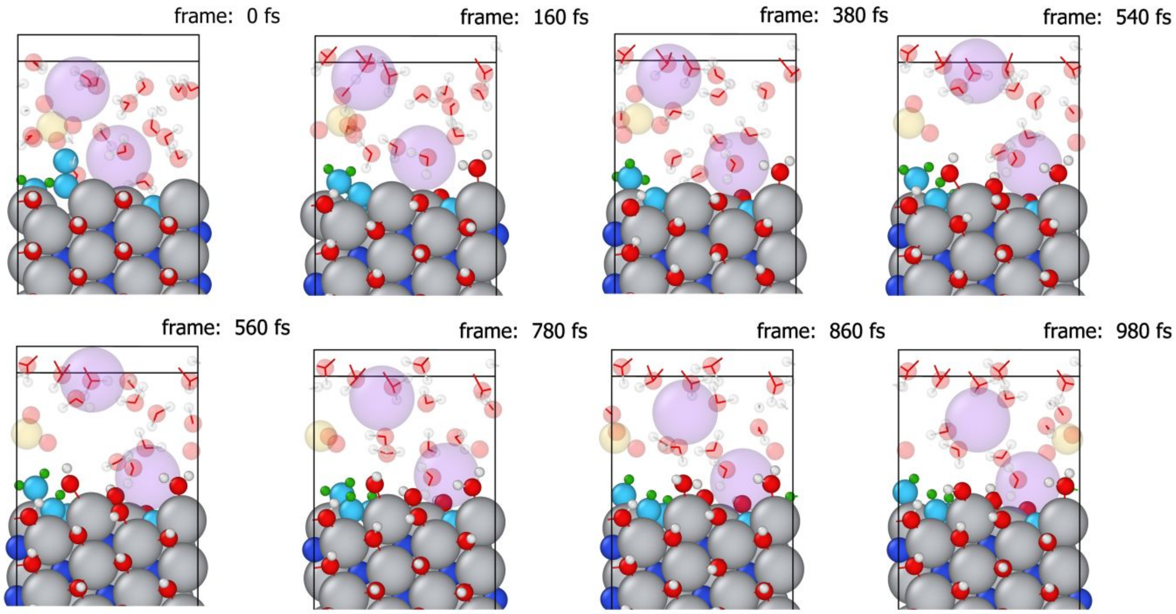

Figure S32. Snapshots from AIMD simulations of NH_3 desorption test and N_2 MvK NRR pathway test on $Ti_2N(OH)_2$ model surface in 2.5 M Na_2SO_4 with vacancy ($\theta = \frac{1}{2}$). Where cyan, blue, white, green, grey, red, purple, and yellow represent nitrogen atoms above the first layer, nitrogen atoms below the first layer, hydrogen, and hydrogen adsorbed on nitrogen, titanium, oxygen, sodium, and sulfur, respectively.

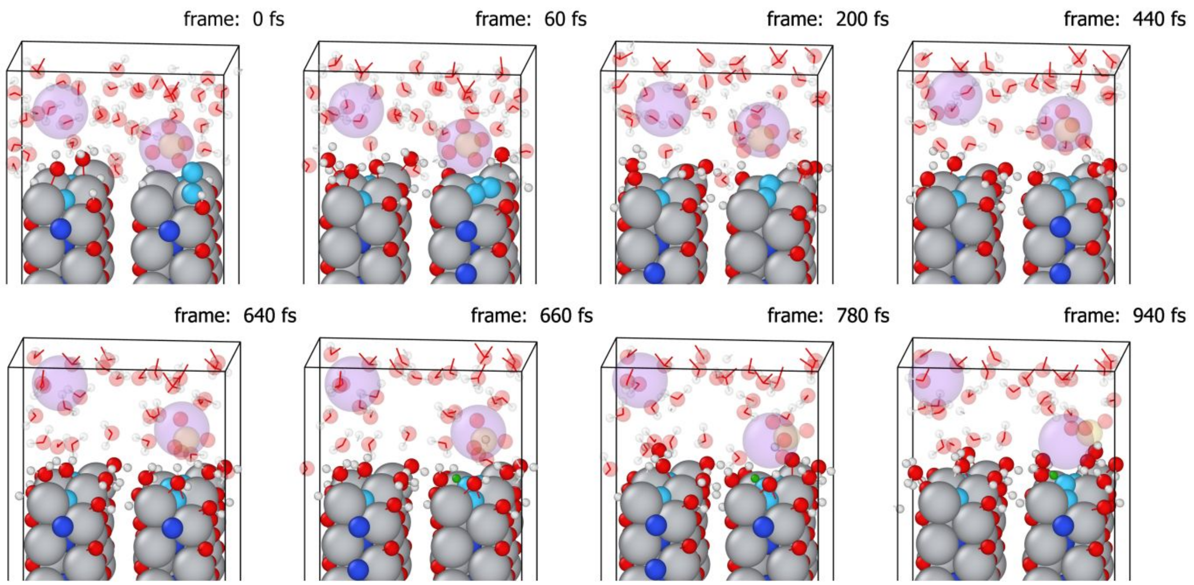

Figure S33. Snapshots from AIMD simulations of N_2 undergo MvK pathway on $Ti_2N(O)_2$ bilayer model surface with edge-facet –OH termination in 1.25 M Na_2SO_4 with nitrogen vacancy ($\theta = \frac{1}{4}$). Where cyan, blue, white, green, grey, red, purple, and yellow represent nitrogen atoms above the first layer, nitrogen atoms below the first layer, hydrogen, and hydrogen adsorbed on nitrogen, titanium, oxygen, sodium, and sulfur, respectively.

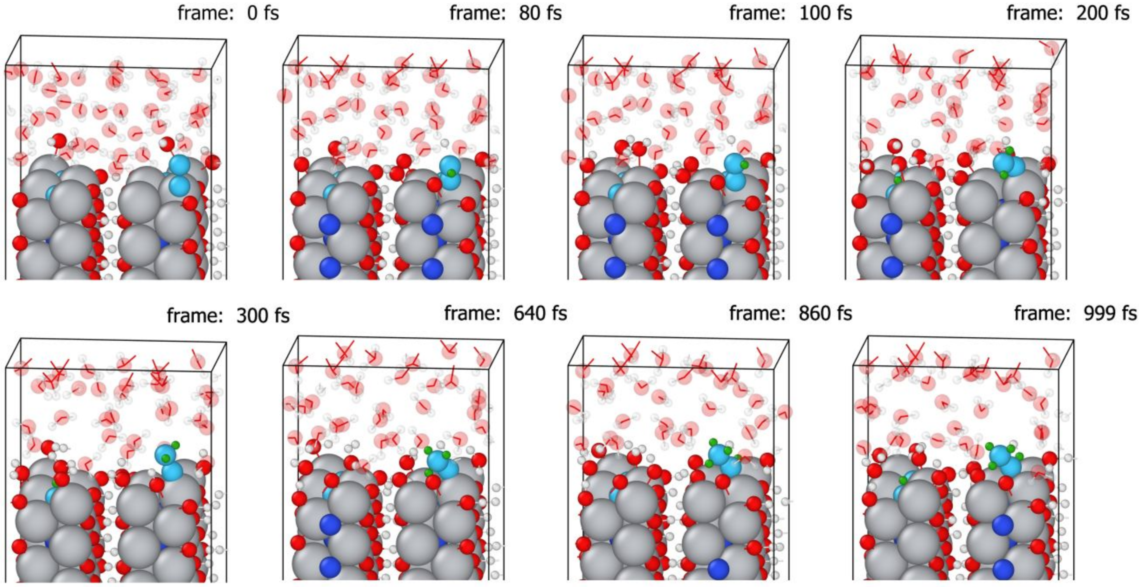

Figure S34. Snapshots from AIMD simulations of N_2 undergo alternative NRR pathway on $Ti_2N(OH)(O)$ bilayer model surface in water with nitrogen vacancy ($\theta = \frac{1}{4}$). Where cyan, blue, white, green, grey, and red represent nitrogen atoms above the first layer, nitrogen atoms below the first layer, hydrogen, and hydrogen adsorbed on nitrogen, titanium, and oxygen, respectively.

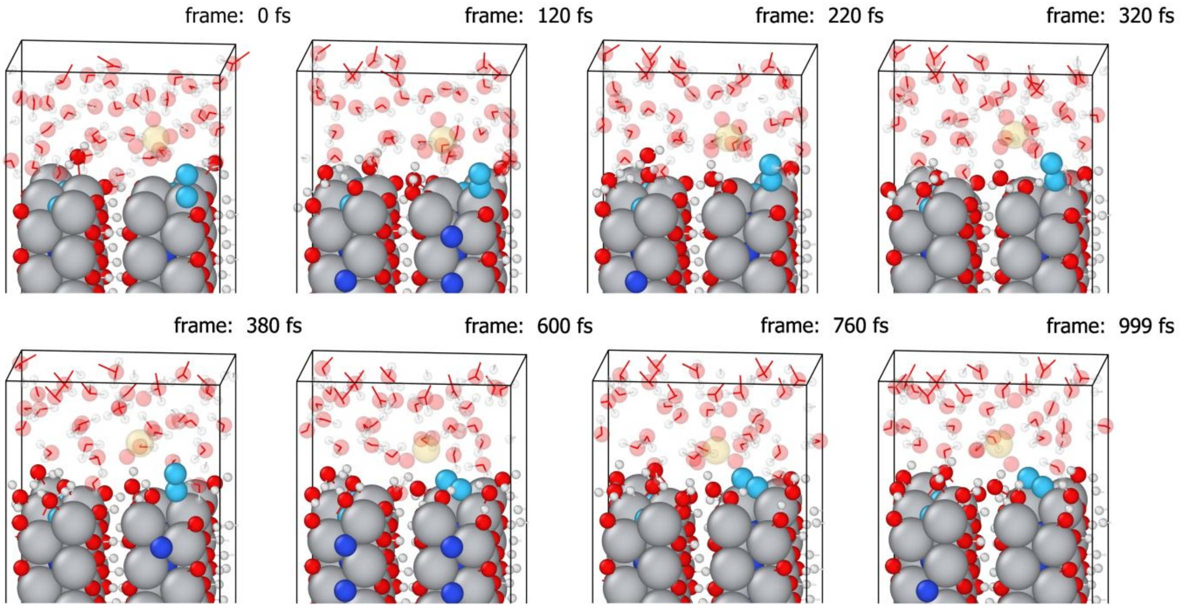

Figure S35. Snapshots from AIMD simulations of N_2 undergo alternative NRR pathway on $Ti_2N(OH)(O)$ bilayer model surface in 1.25 M H_2SO_4 solution with nitrogen vacancy ($\theta = \frac{1}{4}$). Where cyan, blue, white, green, grey, red, and yellow represent nitrogen atoms above the first layer, nitrogen atoms below the first layer, hydrogen, and hydrogen adsorbed on nitrogen, titanium, oxygen, and sulfur, respectively.

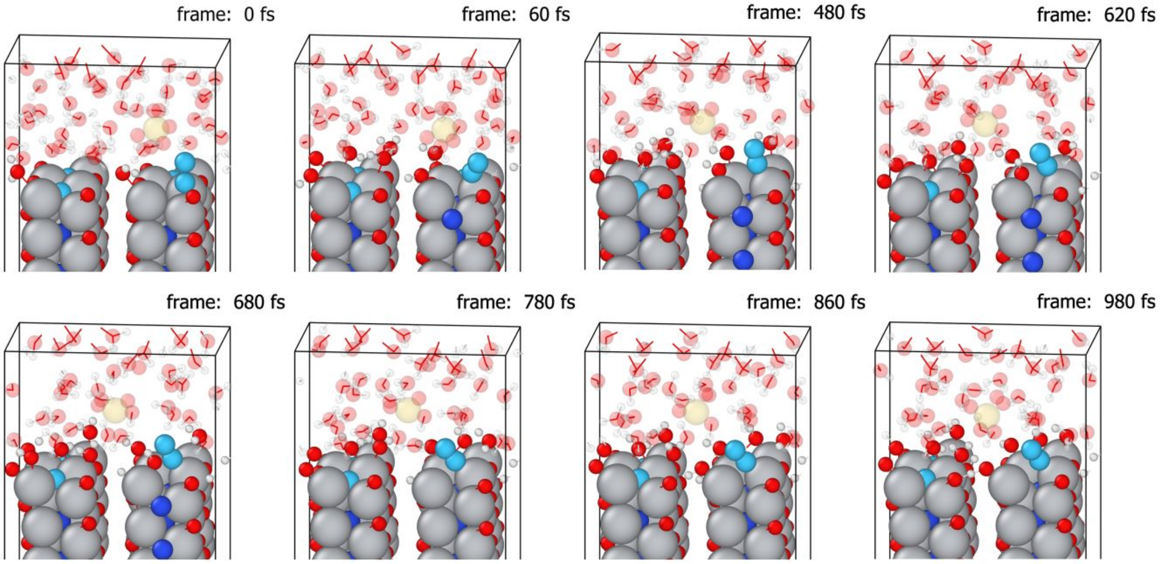

Figure S36. Snapshots from AIMD simulations of N_2 undergo MvK pathway on $Ti_2N(O)_2$ bilayer model surface in 1.25 M H_2SO_4 solution with nitrogen vacancy ($\theta = \frac{1}{4}$). Where cyan, blue, white, green, grey, red, and yellow represent nitrogen atoms above the first layer, nitrogen atoms below the first layer, hydrogen, and hydrogen adsorbed on nitrogen, titanium, oxygen, and sulfur, respectively.

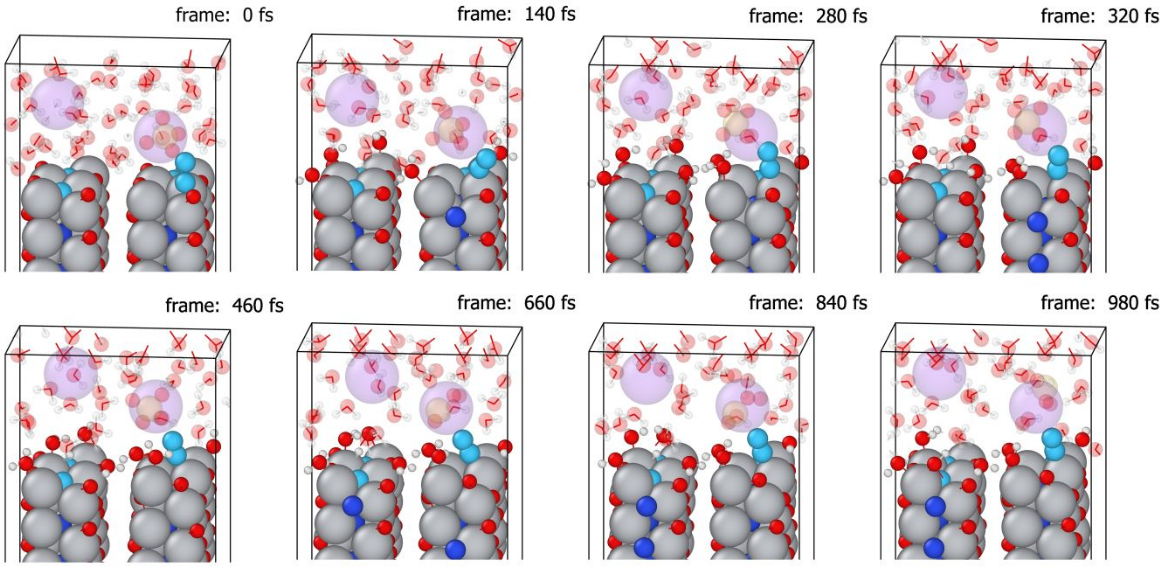

Figure S37. Snapshots from AIMD simulations of N_2 undergo alternative NRR pathway on $Ti_2N(O)_2$ bilayer model surface in 1.25 M Na_2SO_4 solution with nitrogen vacancy ($\theta = \frac{1}{4}$). Where cyan, blue, white, green, grey, red, and yellow represent nitrogen atoms above the first layer, nitrogen atoms below the first layer, hydrogen, and hydrogen adsorbed on nitrogen, titanium, oxygen, and sulfur, respectively.

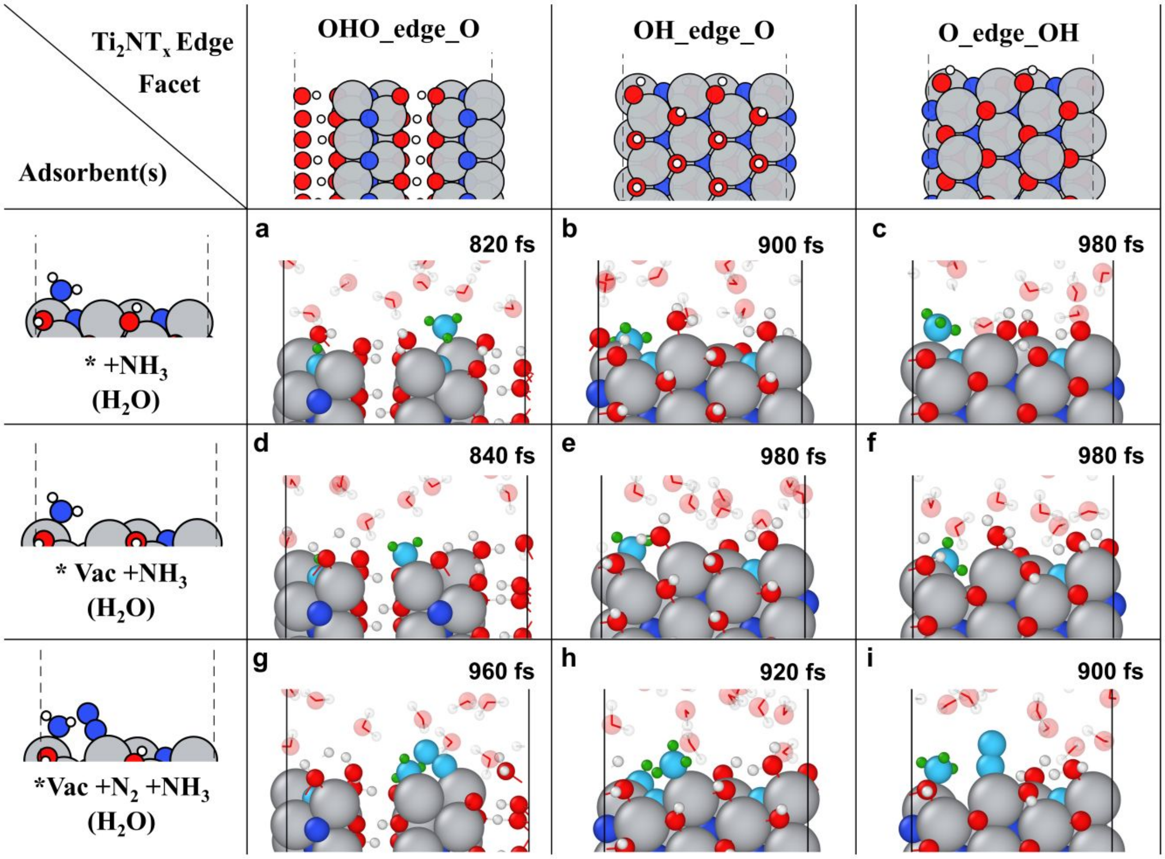

Figure S38. Summary Figure of 1ps AIMD calculation for possible MvK mechanism on different Ti_2NT_x edge facets in $1 \times 2 \times 9$ supercell in H_2O with different adsorbents (NH_3 or/and N_2) without or with nitrogen vacancy ($\theta = \frac{1}{2}$). Illustrations are selected at different snapshots for better visualization. a) Discovered NH_3 hopping and formed NH on top of farther $*N_V$. b) NH_3 did not hop. c) NH_3 did not hop. d) Discovered NH_3 hopping. e) NH_3 did not hop. f) NH_3 did not hop. g) NRR do not proceed; N_2 approached $*_{Vac}$ without NH_3 hopping. h) N_2 undergo MvK mechanism, and interestingly the first pronation of N_2 was carried out by NH_3, and it fill into $*_{Vac}$ and further formed -NH_3 follow $MvK_{as\text{-}dis}$ pathway. i) NRR do not proceed; N_2 approached $*_{Vac}$, and no NH_3 hopping discovered. Color code: gray, red, blue, cyan, white and green represent Ti, O, N below the first layer, N above the first layer, H, and H bonded with N respectively.

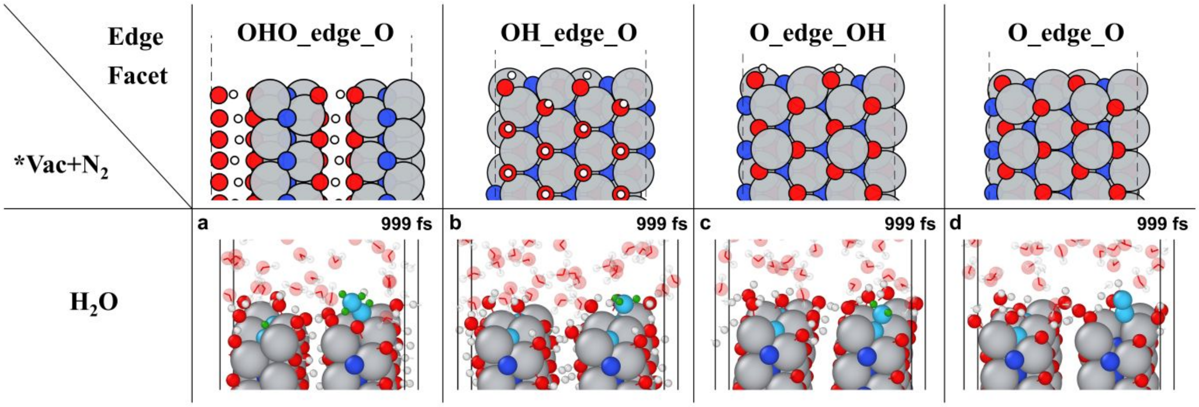

Figure S39. Summary Figure of 1ps AIMD calculation for MvK mechanism on different Ti_2NT_x edge facets in $2 \times 2 \times 9$ supercell in H_2O with nitrogen vacancy ($\theta = \frac{1}{4}$) where N_2 is adsorbed near vacancy initially. Illustrations are selected at different snapshots for better visualization. a) Underwent normal NRR, forming $NHNH_3$ & NH on other $*N_V$ site, with $NHNH_3$ staying at $*_{bri}$. b) Underwent MvK_{dis}, formed NH_3, which further hopped to Ti_{top}. c) Partially filling $*_{Vac}$ and formed NH_2. d) No NRR proceeded; N_2 stayed at $*_{bri}$ without filling $*_{Vac}$. Color code: gray, red, blue, cyan, white and green represent Ti, O, N below the first layer, N above the first layer, H, and H bonded with N respectively.

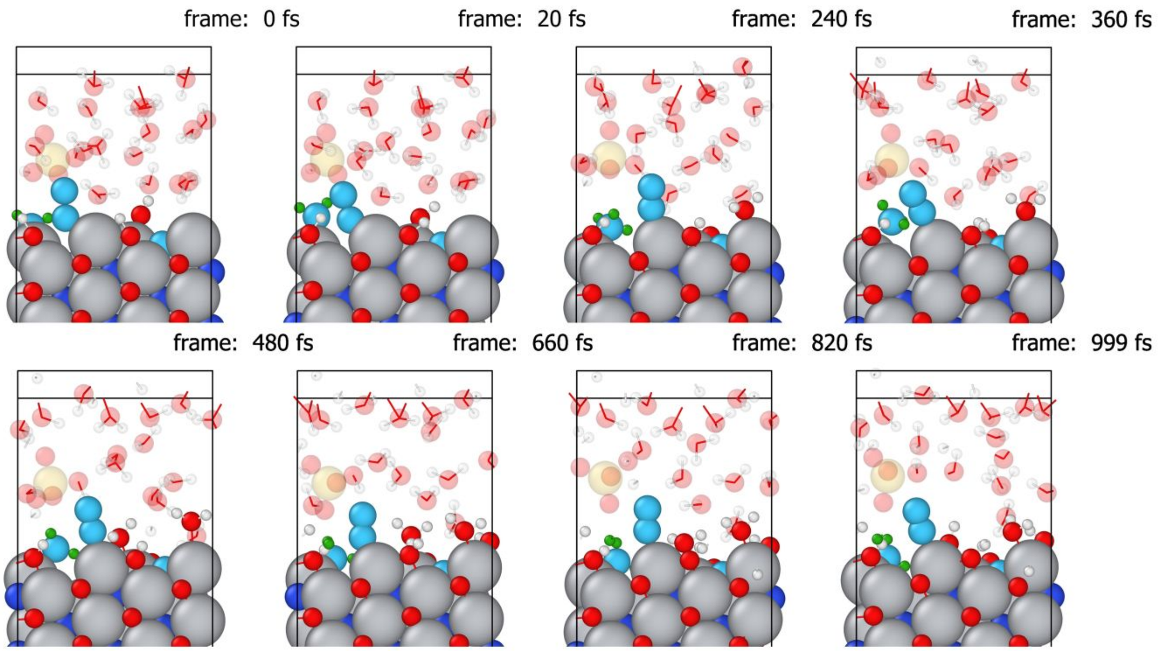

Figure S40. Snapshots from AIMD simulations of NH_3 desorption test and N_2 MvK NRR pathway test on $Ti_2N(O)_2$ model surface with edge-facet –OH termination in 2.5 M H_2SO_4 with vacancy ($\theta = \frac{1}{2}$). Where cyan, blue, white, green, grey, red, and yellow represent nitrogen atoms above the first layer, nitrogen atoms below the first layer, hydrogen, hydrogen adsorbed on nitrogen, titanium, oxygen, and sulfur.

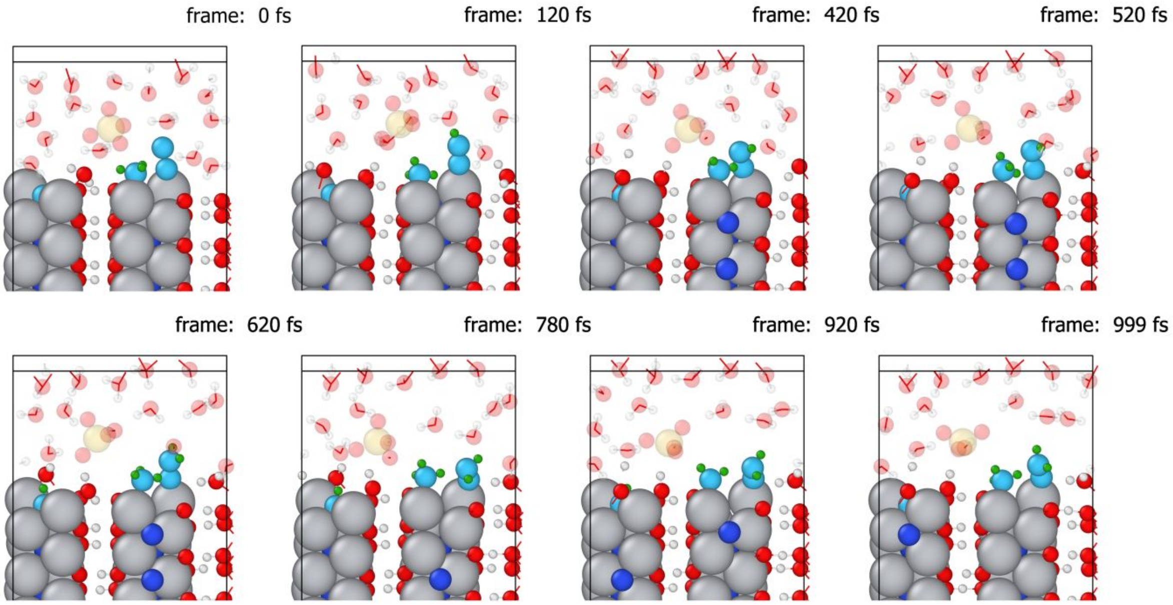

Figure S41. Snapshots from AIMD simulations of NH_3 desorption test and N_2 MvK NRR pathway test on $Ti_2N(OH)(O)$ model surface in 2.5 M H_2SO_4 with vacancy ($\theta = \frac{1}{2}$). Where cyan, blue, white, green, grey, red, and yellow represent nitrogen atoms above the first layer, nitrogen atoms below the first layer, hydrogen, and hydrogen adsorbed on nitrogen, titanium, oxygen, and sulfur, respectively.

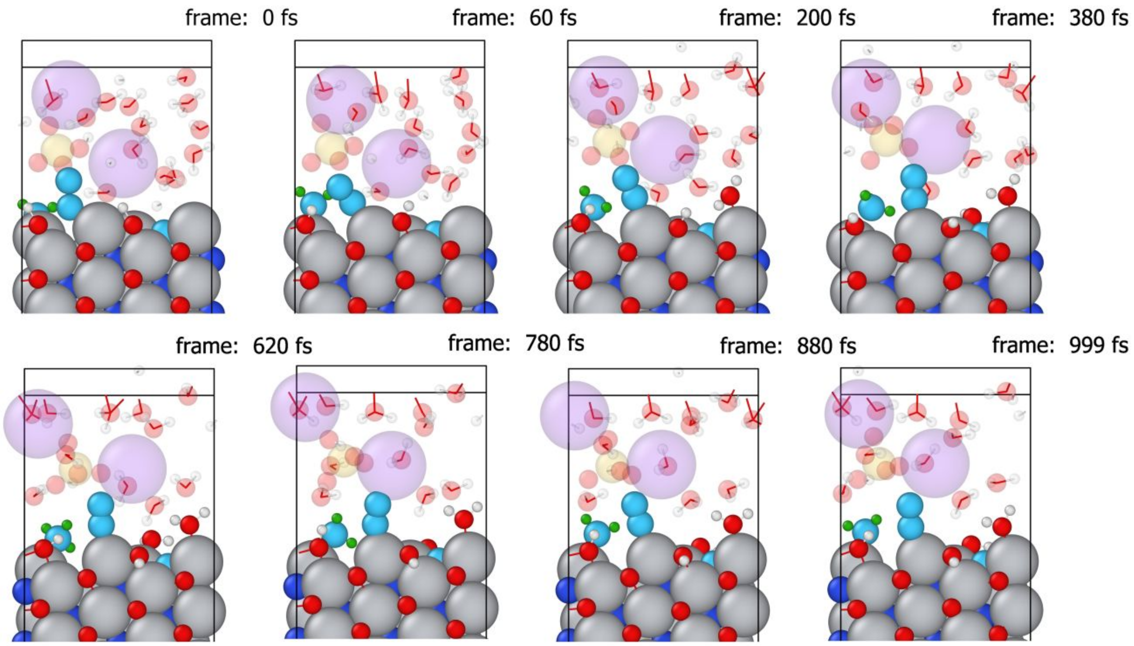

Figure S42. Snapshots from AIMD simulations of NH_3 desorption test and N_2 MvK NRR pathway test on $Ti_2N(O)_2$ model surface with edge-facet –OH termination in 2.5 M Na_2SO_4 with vacancy ($\theta = \frac{1}{2}$). Where cyan, blue, white, green, grey, red, purple, and yellow represent nitrogen atoms above the first layer, nitrogen atoms below the first layer, hydrogen, and hydrogen adsorbed on nitrogen, titanium, oxygen, sodium, and sulfur, respectively.

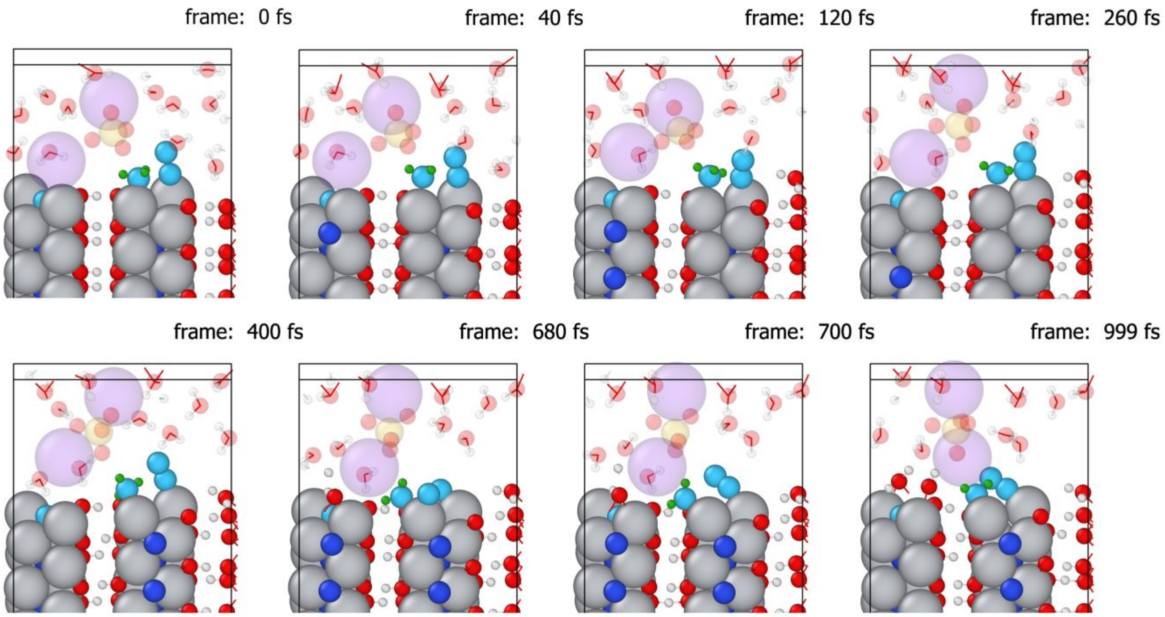

Figure S43. Snapshots from AIMD simulations of NH_3 desorption test and N_2 MvK NRR pathway test on $Ti_2N(OH)(O)$ model surface in 2.5 M Na_2SO_4 with vacancy ($\theta = \frac{1}{2}$). Where cyan, blue, white, green, grey, red, purple, and yellow represent nitrogen atoms above the first layer, nitrogen atoms below the first layer, hydrogen, and hydrogen adsorbed on nitrogen, titanium, oxygen, sodium, and sulfur, respectively.

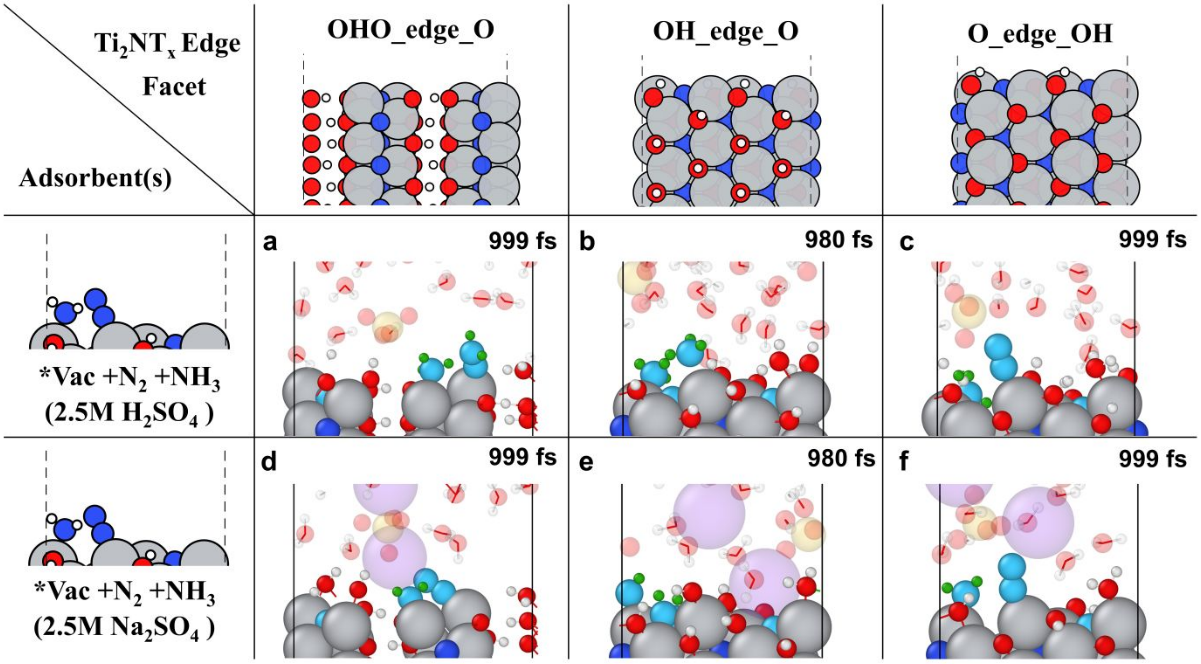

Figure S44. Summary figure of 1ps AIMD calculation for MvK mechanism on different Ti_2NT_x edge facets in $1 \times 2 \times 9$ supercell in neutral (2.5M Na_2SO_4)/acidic electrolyte (2.5M H_2SO_4) with (both NH_3 and N_2) without or with Nitrogen vacancy ($\theta = \frac{1}{2}$). a) Formed -NNH_2, which approached the Ti $*_{bri}$. b) N_2 underwent the $MvK_{as\text{-}dis}$ pathway, filled the vacancy, was protonated by H_2SO_4, and formed NH_2, then further NH_3 on $*_{bri}$. c) NRR didn't proceed; N_2 did not fill $*_{Vac}$. d) NRR didn't proceed; N_2 approached $*_{Vac}$without NH_3 hopping. e) N_2 fully filled the vacancy horizontally and formed NHNH at $*_{Vac}$. f) NRR didn't proceed; N_2 did not fill $*_{Vac}$. Illustrations are selected at different snapshots for better visualization. Color code: gray, red, blue, cyan, white, green, yellow, and purple represent Ti, O, N below first layer, N above first layer, H, and H bonded with N, S, and Na, respectively.

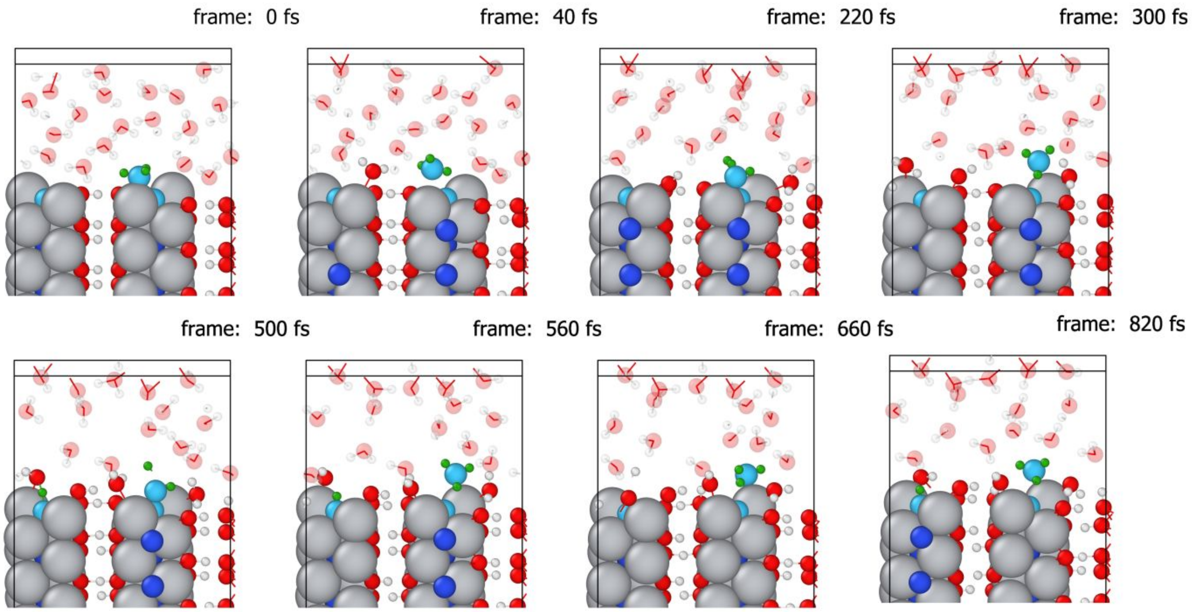

Figure S45. Snapshots from AIMD simulations of NH_3 desorption test on $Ti_2N(OH)(O)$ model surface in water without vacancy. Where cyan, blue, white, green, grey, red represent Nitrogen atoms above first layer, Nitrogen atoms below first layer, hydrogen, hydrogen adsorbed on nitrogen, titanium, oxygen.

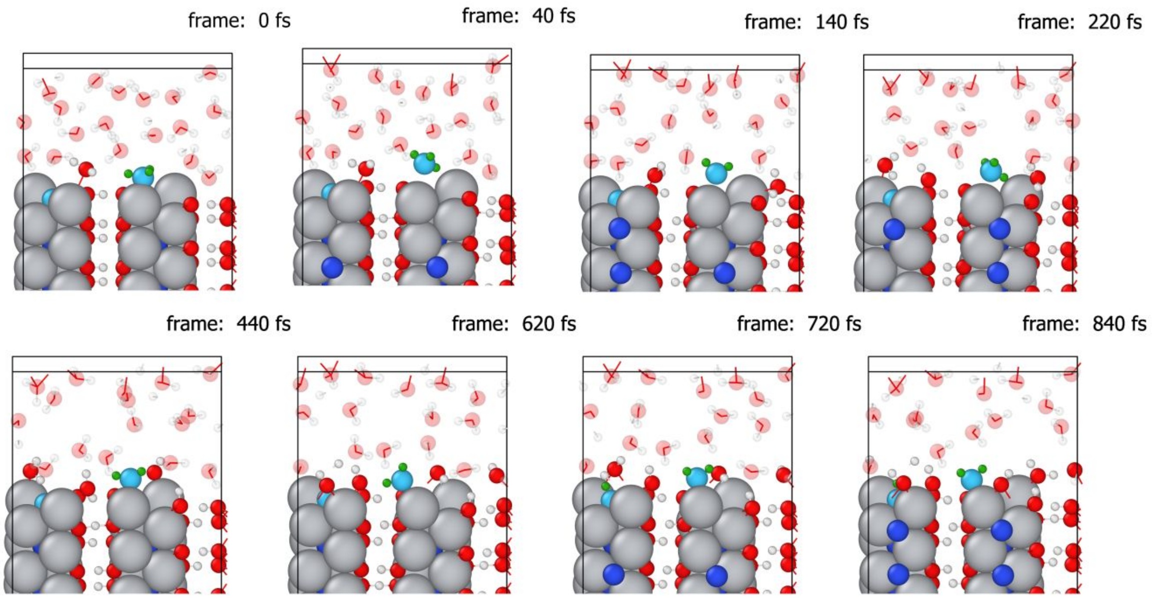

Figure S46. Snapshots from AIMD simulations of NH_3 desorption test on $Ti_2N(OH)(O)$ model surface in water with vacancy ($\theta = \frac{1}{2}$) Where cyan, blue, white, green, grey, red represent Nitrogen atoms above first layer, Nitrogen atoms below first layer, hydrogen, hydrogen adsorbed on nitrogen, titanium, oxygen.

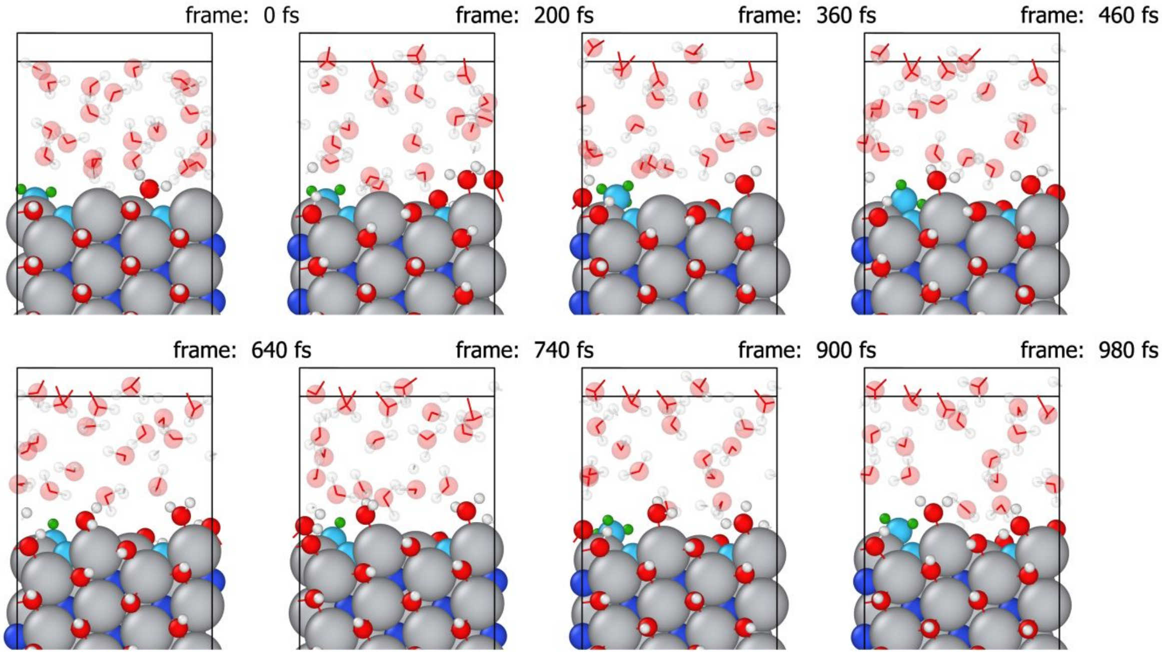

Figure S47. Snapshots from AIMD simulations of NH_3 desorption test on $Ti_2N(OH)_2$ model surface in water without vacancy. Where cyan, blue, white, green, grey, and red represent nitrogen atoms above the first layer, nitrogen atoms below the first layer, hydrogen, and hydrogen adsorbed on nitrogen, titanium, and oxygen, respectively.

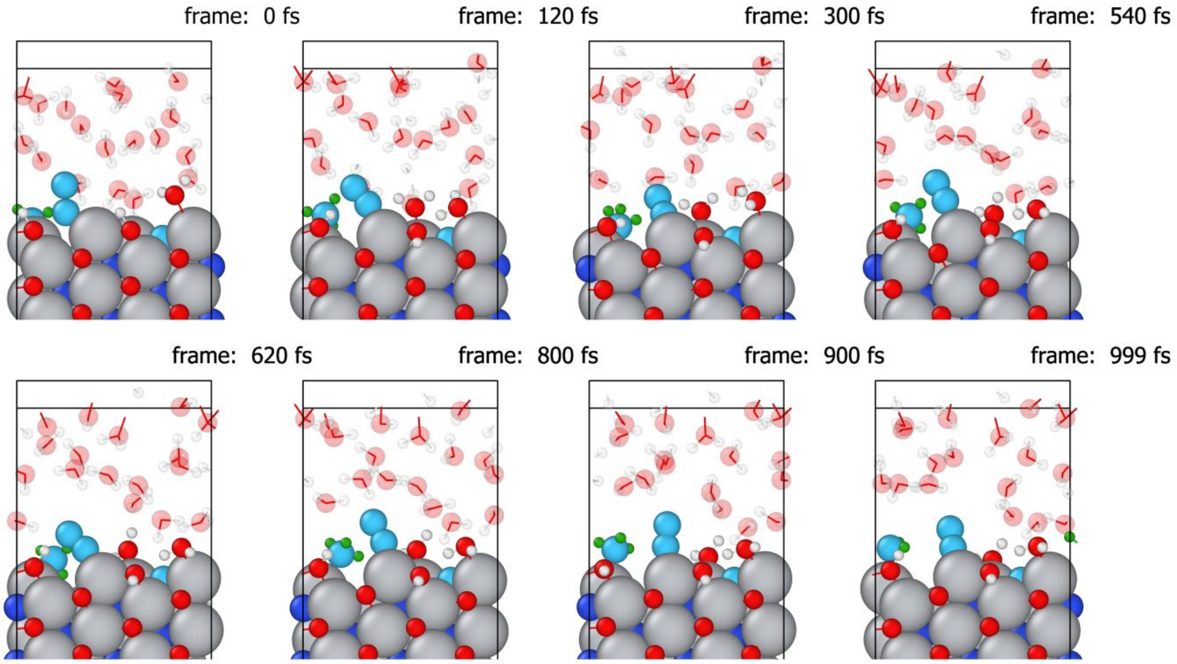

Figure S48. Snapshots from AIMD simulations of NH_3 desorption test and N_2 MvK NRR pathway test on $Ti_2N(O)_2$ model surface with edge-facet –OH termination in water with vacancy ($\theta = \frac{1}{2}$). Where cyan, blue, white, green, grey, and red represent nitrogen atoms above the first layer, nitrogen atoms below the first layer, hydrogen, and hydrogen adsorbed on nitrogen, titanium, and oxygen, respectively.

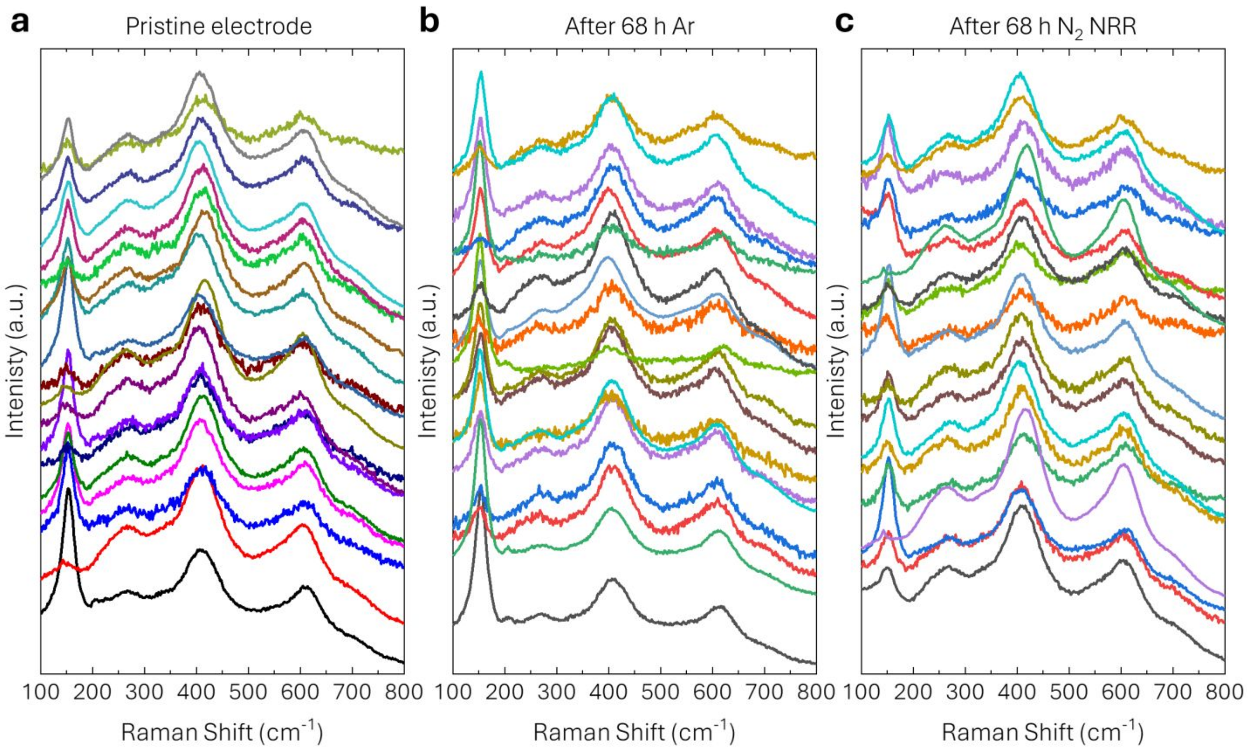

Figure S49. Raman spectra of Ti_2NT_x MNene coated on glassy carbon electrode collected at 19 different spots **a)** prior to NRR conditions, **b)** after 68-hour NRR under Ar-saturation to generate N vacancies, and **c)** after 68-hour NRR under N_2-saturation after Ar-saturation to replenish previous N vacancies.

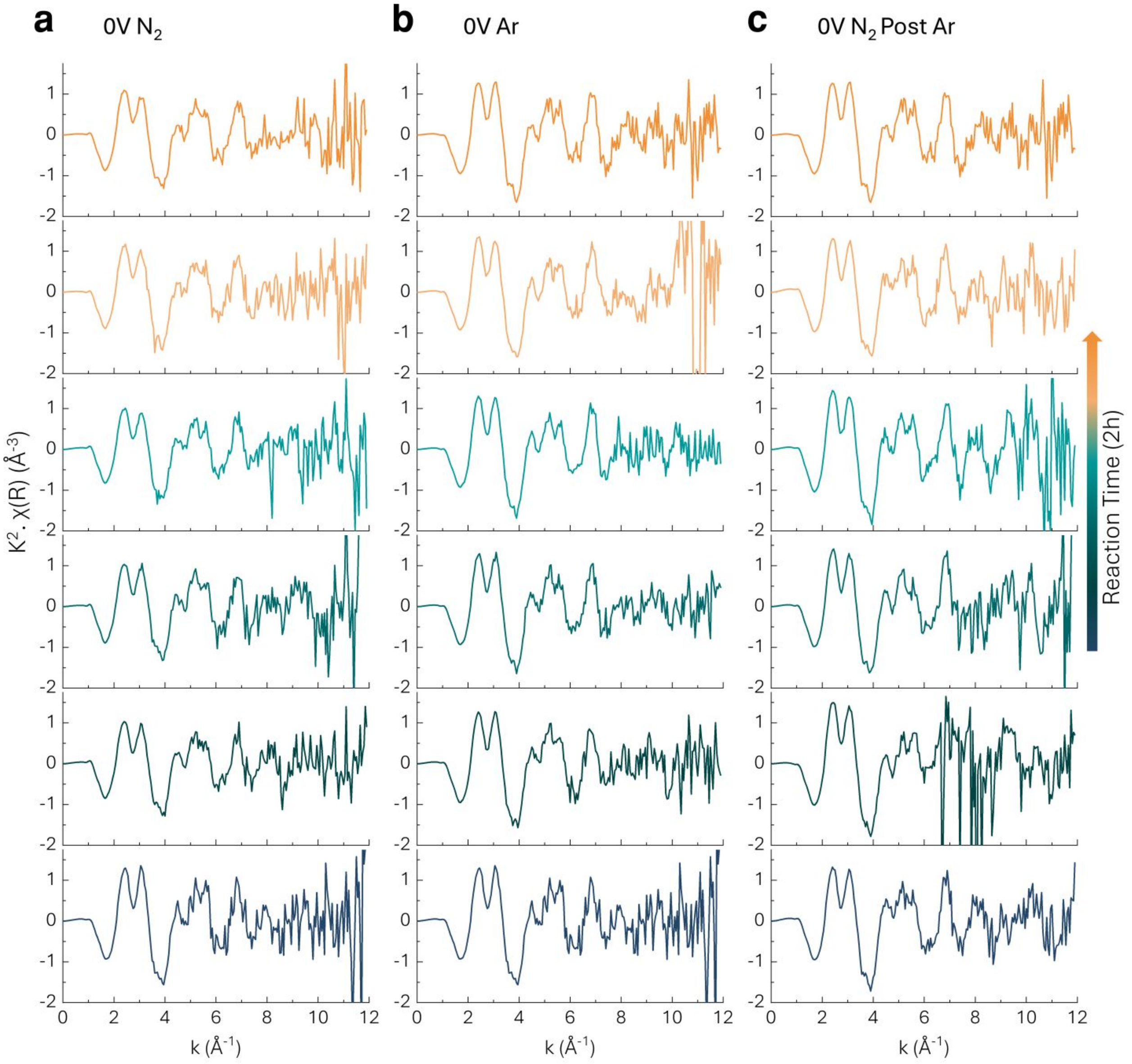

Figure S50. Operando XAS: k-space spectra for Ti_2NT_x after being exposed to **a)** N_2 as a reactant gas, **b)** Ar as an inert atmosphere gas, and **c)** N_2 reintroduction as a regeneration gas.

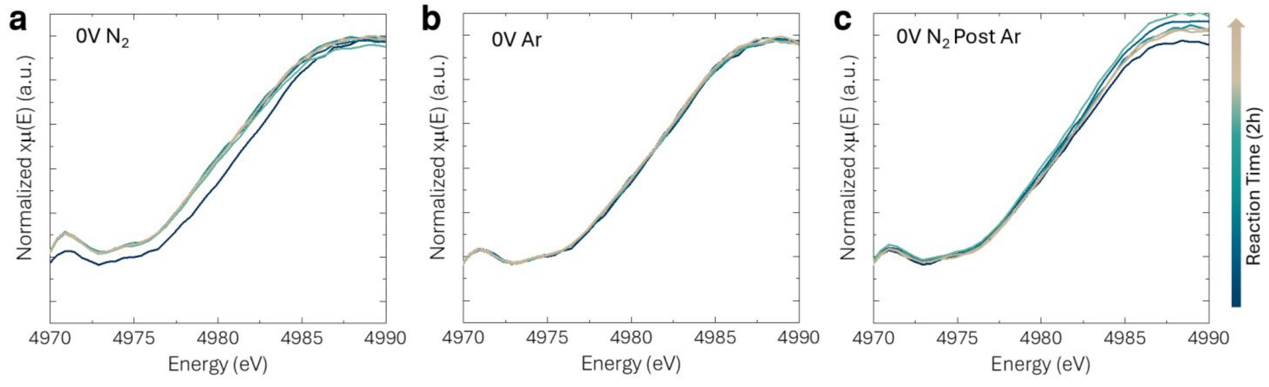

Figure S51. XANES region of the normalized Ti K-edge XAS spectra for Ti_2NT_x catalyst after being exposed to **a)** N_2 as a reactant gas, **b)** Ar as an inert atmosphere gas, and **c)** N_2 reintroduction as a regeneration gas.

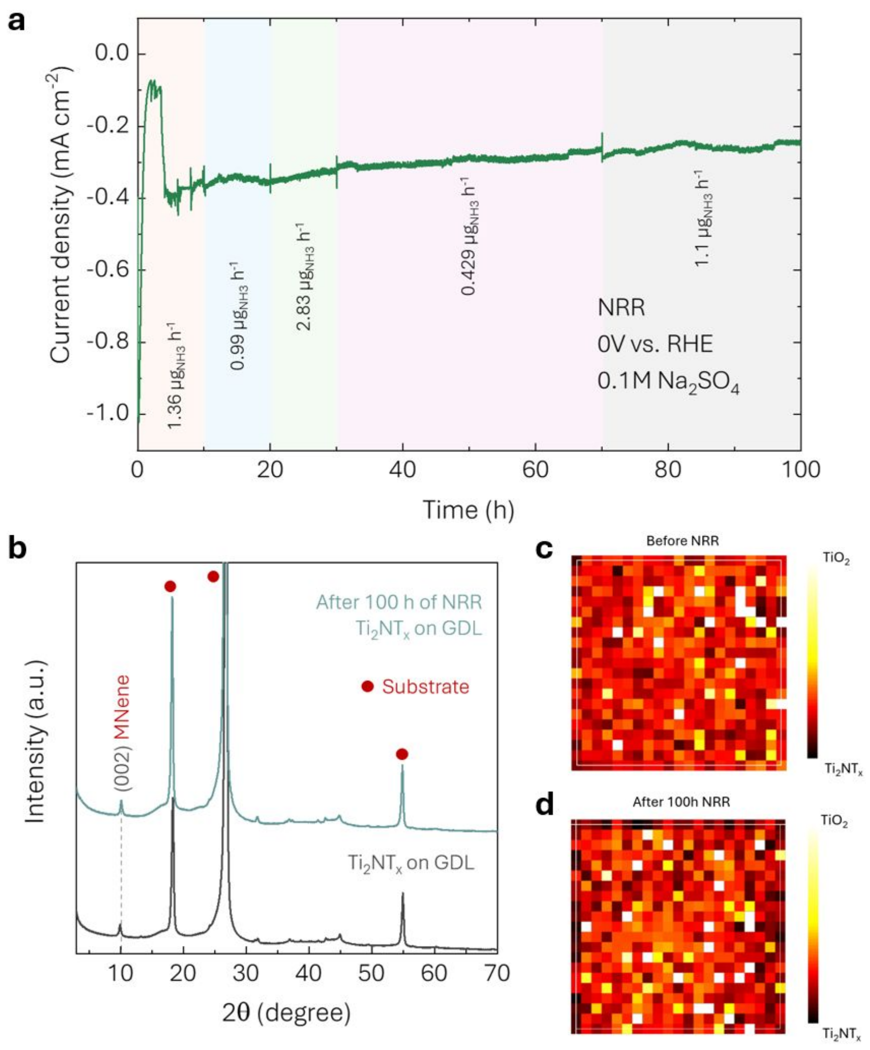

Figure S52. a) Chronoamperometry curve and ammonia yield rate of the Ti_2NT_x electrode measured periodically under continuous N_2 feed during 100 h of electrolysis in 0.1 M Na_2SO_4 at 0 V vs RHE, using a Ti_2NT_x coated gas diffusion electrode. **b)** XRD patterns of the Ti_2NT_x electrode before and after 100 h of NRR. Raman mapping of the electrode surface over a 1 × 1 cm² area, showing the intensity ratio of the TiO_2 peak (~150 cm^{-1}) to the Ti_2NT_x peak (~420 cm^{-1}) **c)** before NRR and **d)** after 100 h of NRR. A transition toward a bright yellow color indicates increased TiO_2 content relative to Ti_2NT_x at the particular spot. Mapping was performed over 441 discrete spots across the area.

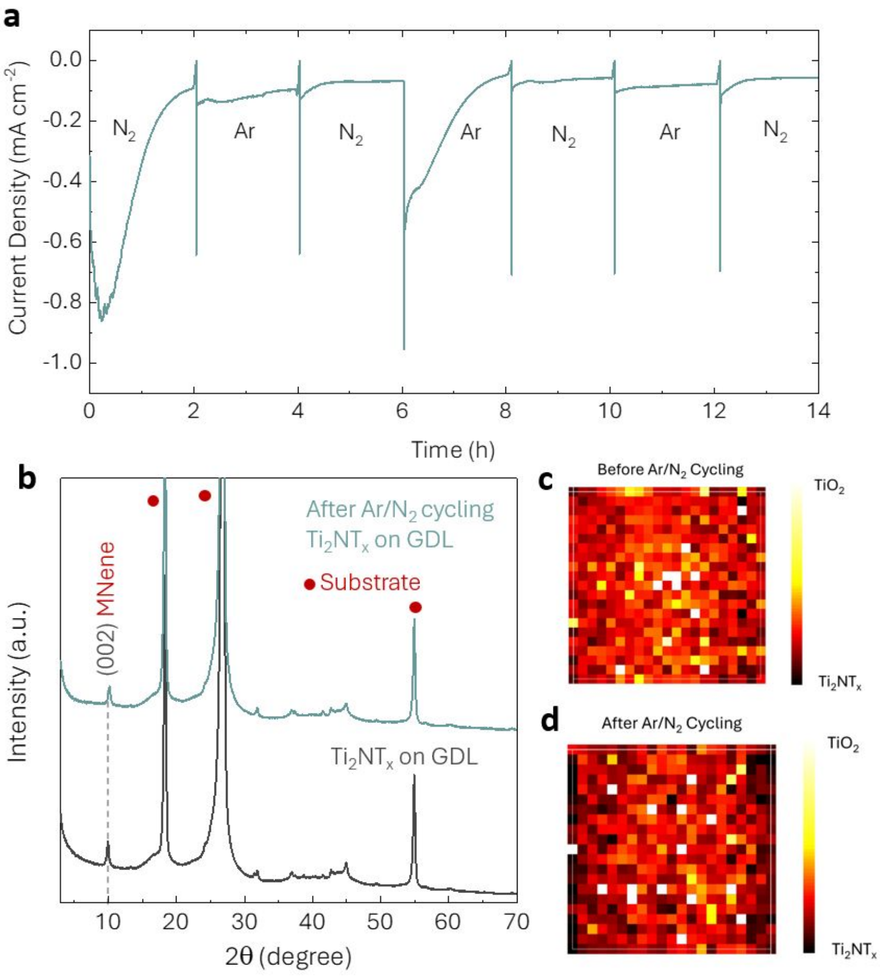

Figure S53. a) Chronoamperometry curve of the Ti_2NT_x electrode during N_2/Ar feed cycling in 0.1 M Na_2SO_4 at 0 V vs RHE. The gas feed was alternated every 2 h. The experiment was conducted using a Ti_2NT_x coated gas diffusion electrode (see **Figure S15a**). **b)** XRD patterns of the Ti_2NT_x electrode before and after 14 h of N_2/Ar cycling. **c–d)** Raman mapping of the electrode surface over a 1 × 1 cm² area, showing the intensity ratio of the TiO_2 peak (~150 cm^{-1}) to the Ti_2NT_x peak (~420 cm^{-1}). **Panel c** shows the Raman map before cycling, and **panel d** after N_2/Ar cycling.

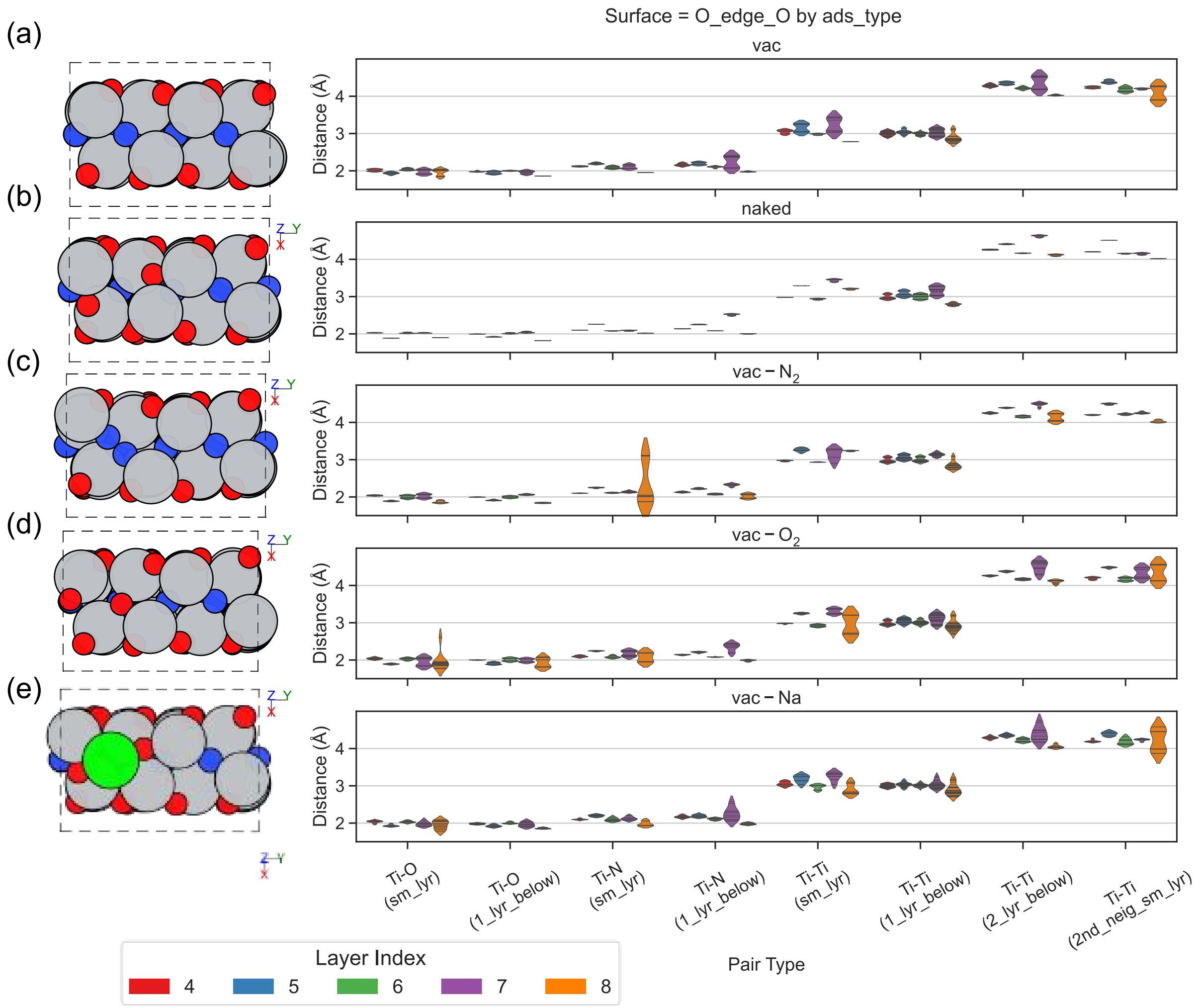

Figure S54. Violin plot of pairwise distance between Ti-O/Ti-N/Ti-Ti of different coordinates across different charges from DFT optimized geometry of grouping by surface layer index, different MNene surface and adsorption type. a-e) surface Ti_2NO_2 (O_edge_O) with nitrogen vacancy, naked surface, nitrogen vacancy filling N_2, O_2, and Na. Geometries data in each subplot includes pairwise distance of geometry with 0, 1, and 4 electron additions. The illustration included along each subplot is neutral charged. Overall, Ti-O and Ti-N pairwise distance change mainly due to nitrogen vacancy filling with N_2 or O_2 instead of charged surface, which further supports EXAFS radial distance change observation for MvK mechanism. It further shows the pairwise distance change of Ti-N due to MvK mechanism in vacancy filling step in c, and possibly deactivation by oxygen vacancy filling in d.

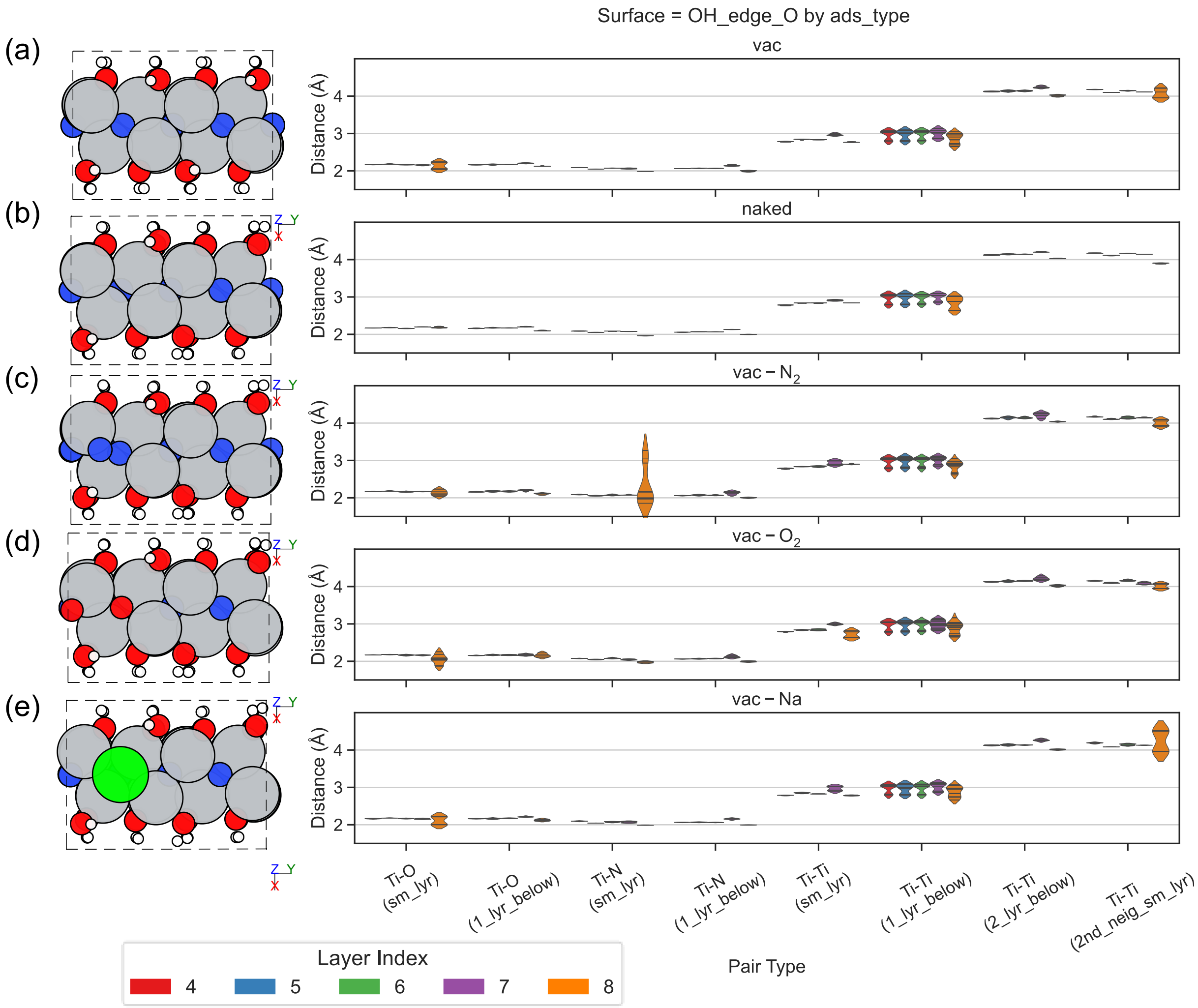

Figure S55. Violin plot of pairwise distance between Ti-O/Ti-N/Ti-Ti of different coordinates across different charges from DFT optimized geometry of grouping by surface layer index, different MNene surface and adsorption type. a-e) surface $Ti_2N(OH)_2$ (OH_edge_O) with nitrogen vacancy, naked surface, nitrogen vacancy filling N_2, O_2, and Na. Geometries data in each subplot includes pairwise distance of geometry with 0, 1, and 4 electron additions. The illustration included along each subplot is neutral charged. Overall, Ti-O and Ti-N pairwise distance change mainly due to nitrogen vacancy filling with N_2 or O_2 instead of charged surface, which further supports EXAFS radial distance change observation for MvK mechanism. It further shows the pairwise distance change of Ti-N due to MvK mechanism in vacancy filling step in c, and possibly deactivation by oxygen vacancy filling in d.

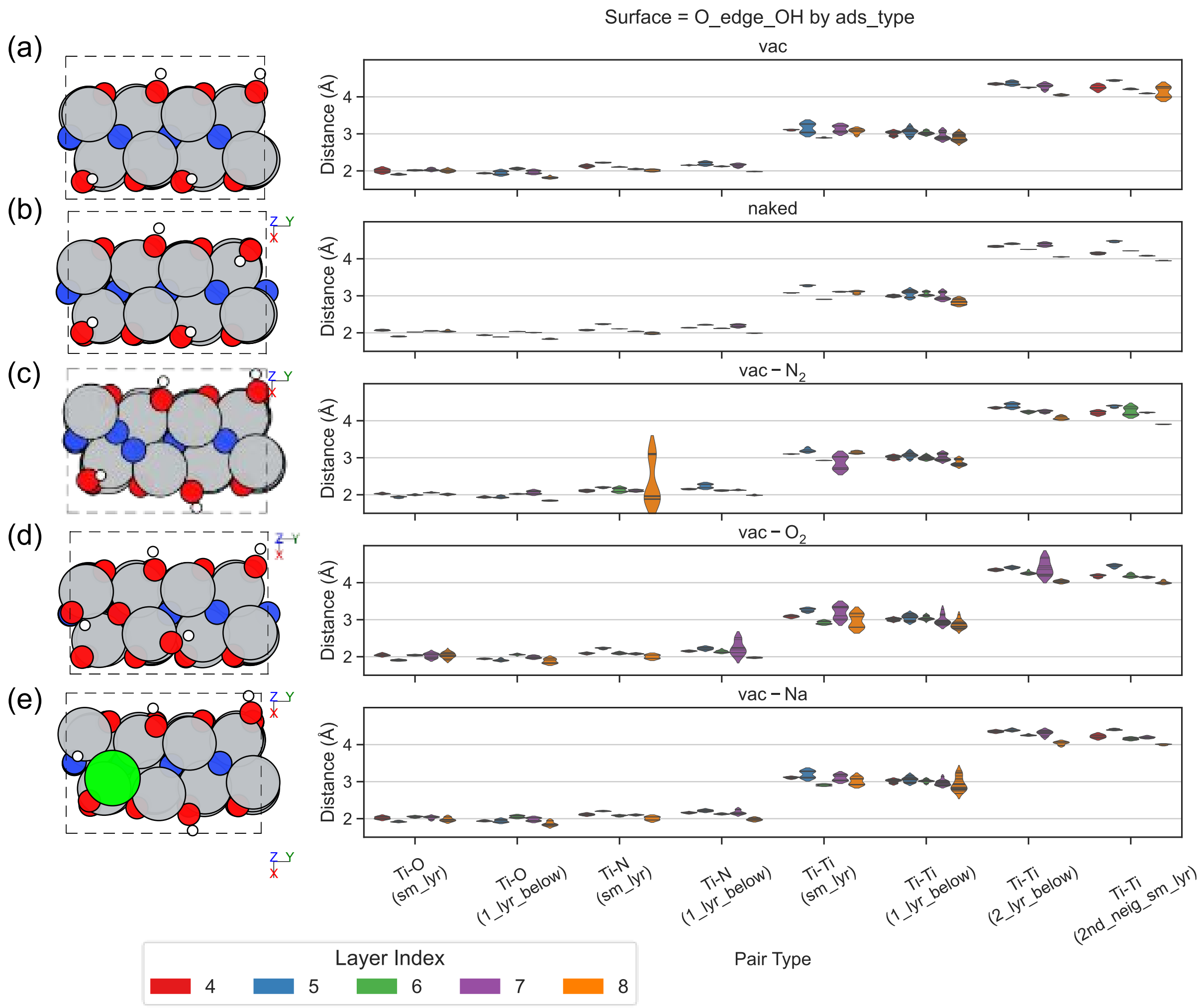

Figure S56. Violin plot of pairwise distance between Ti-O/Ti-N/Ti-Ti of different coordinates across different charges from DFT optimized geometry of grouping by surface layer index, different MNene surface, and adsorption type. a-e) surface Ti_2NO_2 with edge -OH termination (O_edge_OH) with nitrogen vacancy, naked surface, nitrogen vacancy filling N_2, O_2, and Na. Geometries data in each subplot includes pairwise distance of geometry with 0, 1, and 4 electron additions. The illustration included along each subplot is neutral charged. Overall, Ti-O and Ti-N pairwise distance change mainly due to nitrogen vacancy filling with N_2 or O_2 instead of charged surface, which further supports EXAFS radial distance change observation for MvK mechanism. It further shows the pairwise distance change of Ti-N due to MvK mechanism in vacancy filling step in c, and possibly deactivation by oxygen vacancy filling in d.

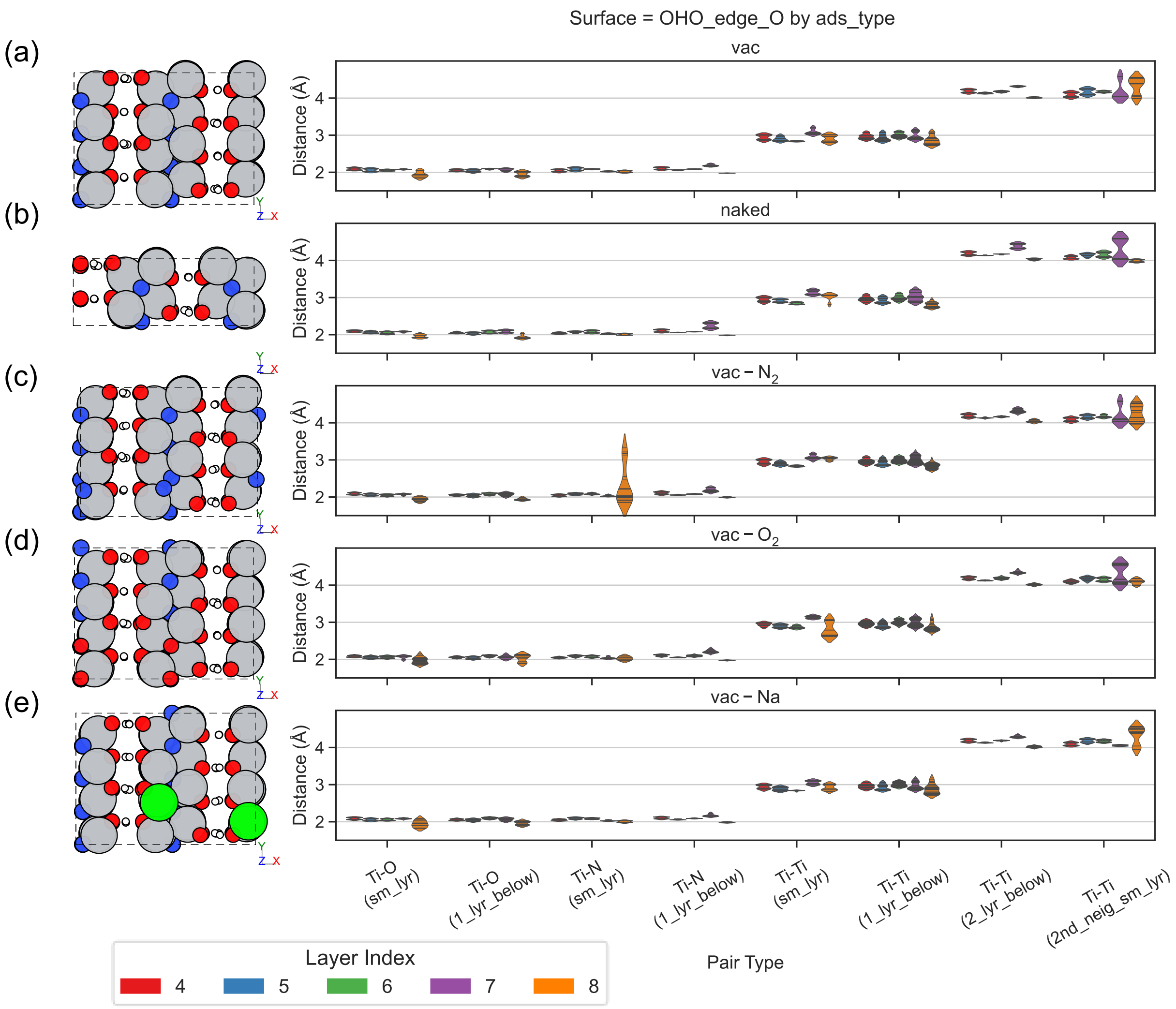

Figure S57. Violin plot of pairwise distance between Ti-O/Ti-N/Ti-Ti of different coordinates across different charges from DFT optimized geometry of grouping by surface layer index, different MNene surface, and adsorption type. a-e) surface $Ti_2N(OH)O$ (OHO_edge_O) with nitrogen vacancy, naked surface, nitrogen vacancy filling N_2, O_2, and Na. Geometries data in each subplot includes pairwise distance of geometry with 0, 1, and 4 electron additions. The illustration included along each subplot is neutral charged. Overall, Ti-O and Ti-N pairwise distance change mainly due to nitrogen vacancy filling with N_2 or O_2 instead of charged surface, which further supports EXAFS radial distance change observation for MvK mechanism. It further shows the pairwise distance change of Ti-N due to MvK mechanism in vacancy filling step in c, and possibly deactivation by oxygen vacancy filling in d.

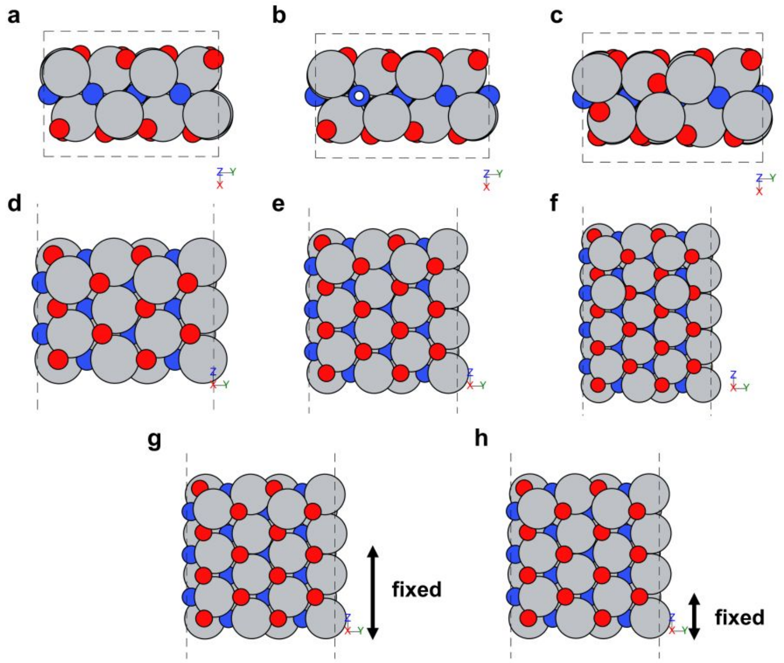

Figure S58. The illustration of convergence test for edge slab model setup. a) Ti_2NO_{2sur}. b) Ti_2NO_{2sur+H}. c) Ti_2NO_{2sur-N} d) 1 × 2 × 5 edge slab. e) 1 × 2 × 7 edge slab. f) 1 × 2 × 9 edge slab. g) fixbot4 h) fixbot2 layer setup.

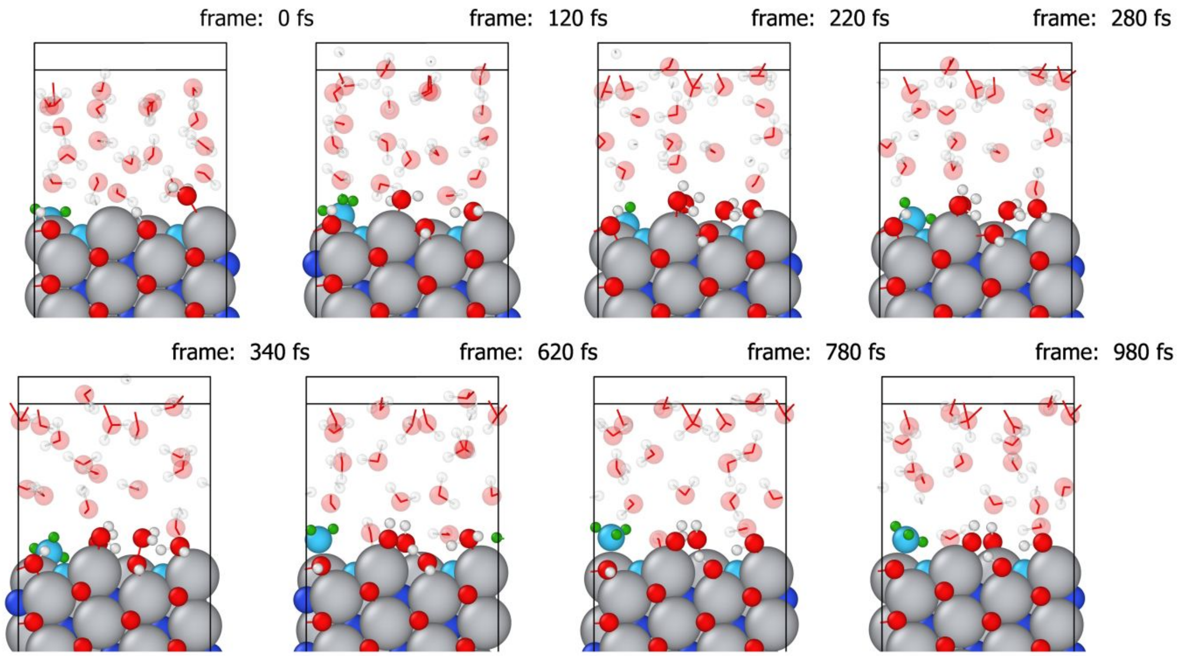

Figure S59. Snapshots from AIMD simulations of NH_3 desorption test on $Ti_2N(O)_2$ model surface with edge-facet –OH termination in water without vacancy. Where cyan, blue, white, green, grey, and red represent nitrogen atoms above the first layer, nitrogen atoms below the first layer, hydrogen, and hydrogen adsorbed on nitrogen, titanium, oxygen, respectively.

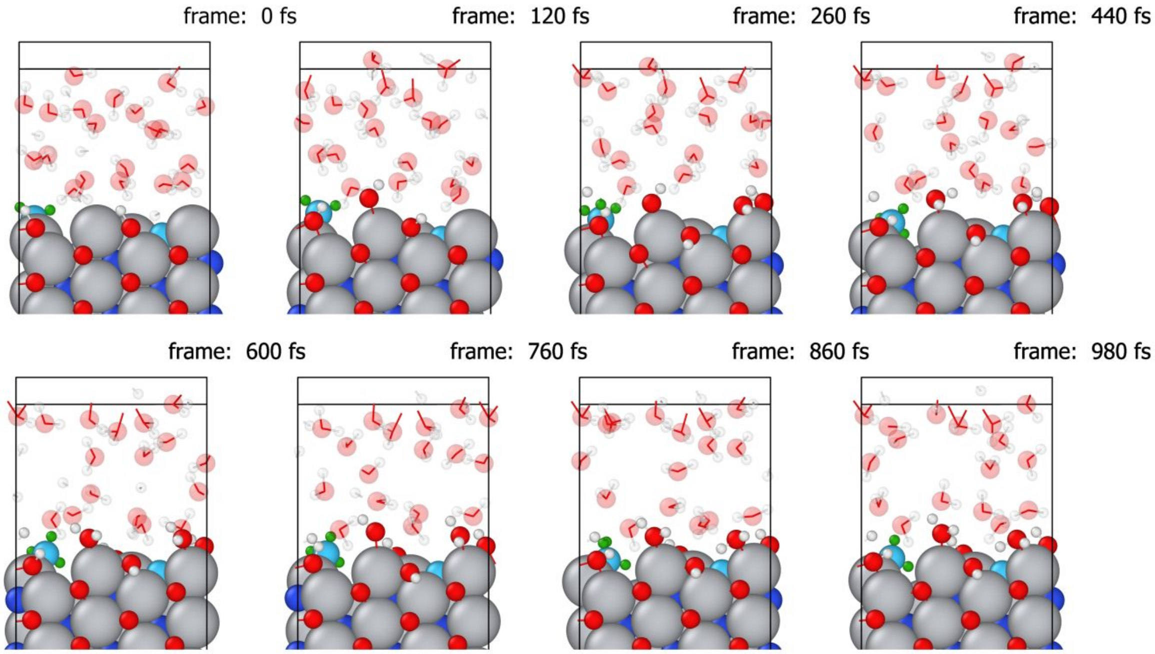

Figure S60. Snapshots from AIMD simulations of NH_3 desorption test on $Ti_2N(O)_2$ model surface with edge-facet –OH termination in water with vacancy ($\theta = \frac{1}{2}$). Where cyan, blue, white, green, grey, and red represent nitrogen atoms above the first layer, nitrogen atoms below the first layer, hydrogen, and hydrogen adsorbed on nitrogen, titanium, and oxygen, respectively.

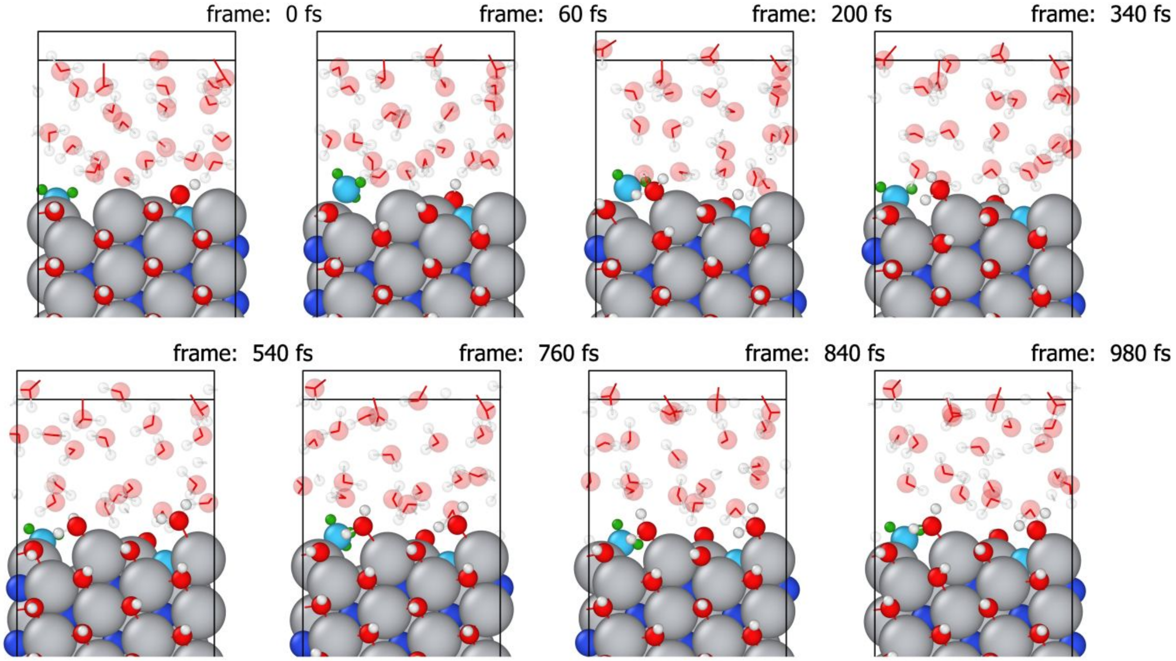

Figure S61. Snapshots from AIMD simulations of NH_3 desorption test on $Ti_2N(OH)_2$ model surface in water with vacancy ($\theta = \frac{1}{2}$). Where cyan, blue, white, green, grey, red represent Nitrogen atoms above first layer, nitrogen atoms below first layer, hydrogen, hydrogen adsorbed on nitrogen, titanium, oxygen.

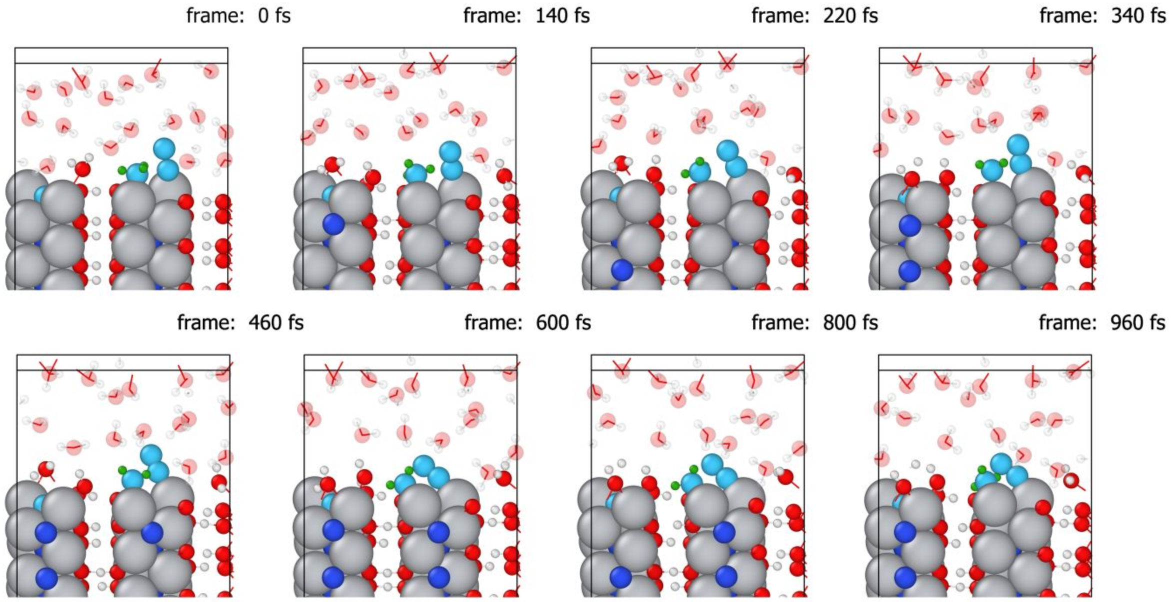

Figure S62. Snapshots from AIMD simulations of NH_3 desorption test and N_2 MvK NRR pathway test on $Ti_2N(OH)(O)$ model surface in water with vacancy ($\theta = \frac{1}{2}$). Where cyan, blue, white, green, grey, red represent Nitrogen atoms above first layer, Nitrogen atoms below first layer, hydrogen, hydrogen adsorbed on nitrogen, titanium, oxygen.

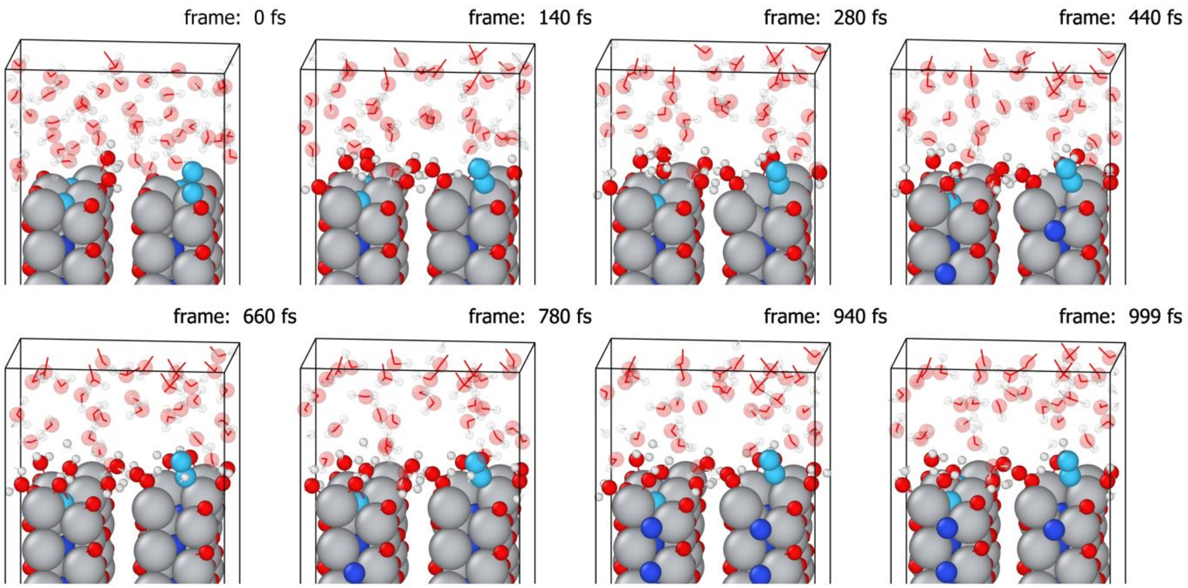

Figure S63. Snapshots from AIMD simulations of N_2 undergo MvK pathway on $Ti_2N(O)_2$ bilayer model surface in water with Nitrogen vacancy ($\theta = \frac{1}{4}$). Where cyan, blue, white, green, grey, red represent Nitrogen atoms above first layer, Nitrogen atoms below first layer, hydrogen, hydrogen adsorbed on nitrogen, titanium, oxygen.

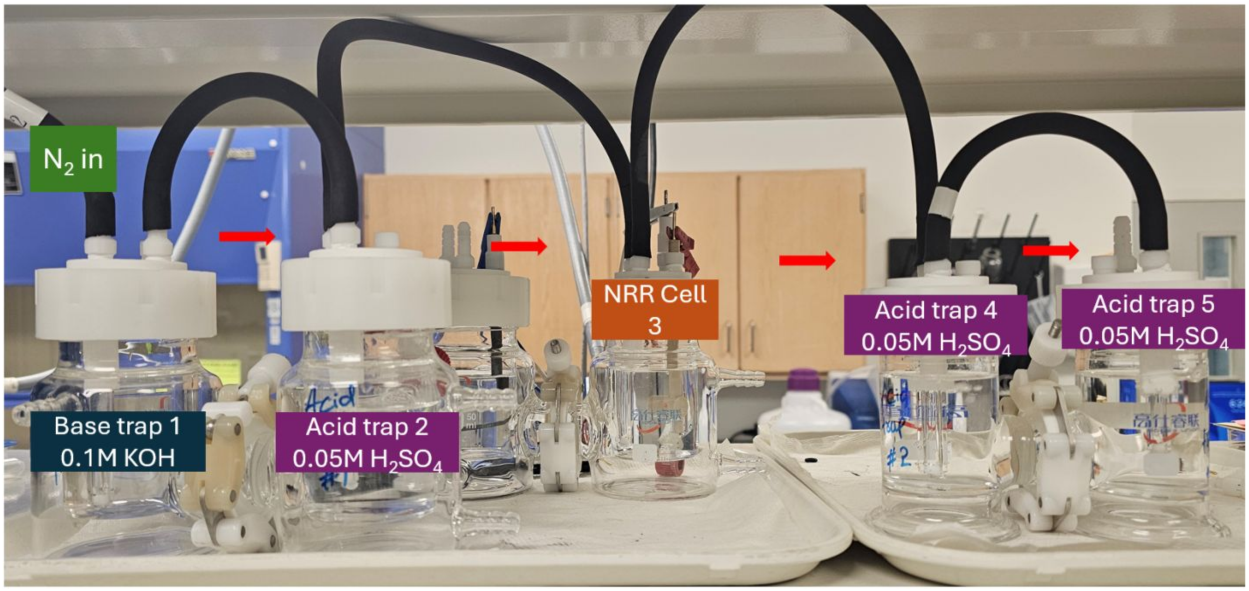

Figure S64. Photograph of NRR electrochemical setup with acid and base traps.

Supplementary Tables

Table S1. DFT energies, Zero-point energy and entropy correction at 25 °C (Values in eV). The entropies of H_2(g), N_2(g), and NH_3(g) are calculated at 1 atm. To provide accurate free energy calculation of nitrogen gas, the free energy correction nitrogen gas is calculated based on the NRR experiment reaction energy change (-0.34 eV) to correct the N_2 gas. All table energies have units of eV.

	**E_0**	**ZPE**	**TS**	**Correction to G**	**G**
NH_3	-19.558	0.9119	0.5946	0.4211	-19.137
H_2	-6.766	0.2678	0.4038	-0.0461	-6.812
N_2 (calculated)	-16.64	0.1507	0.5914	-0.3508	-16.992
N_2 (derived back from exp react energy) (N_2= -(-0.34*) +2NH_3 - 3H_2)	-	-	-	-	-17.498

*Gibbs free energy change of N_2(g) + 3H_2(g) = 2NH_3(g) at 298K from experiment is 33.2 kJ/mol [2] = 0.34eV

Table S2. Adsorption free energy calculation for different surfaces and adsorption type. Higher O_2 adsorption free energies result from O_2 bond-breaking during optimization. Overall, order of adsorption tendency on refilling MvK surface is O_2 >> N_2 > Na, suggesting the importance of isolation of oxygen during NRR to prevent deactivation.

Surface	Adsorption type	Surface E_0 (eV)	Surface ZPE (eV)	Absorbate Free energy (eV)	Vacancy energy (eV)	Adsorption free energy (eV)
$Ti_2N(OH)O$ (OHO_edge_O)	naked	-1802.869			-1778.394	
	vac-25%[#]	-1790.909			-1778.394	
	vac-N_2	-1818.548	0.380	-34.996[†]	-1778.394	-2.389[†]
	vac-Na	-1783.005	0.052	-0.530[†‡]	-1778.394	-2.015[†]
	vac-O_2	-1820.958	0.318	-19.838[†]	-1778.394	-11.204[†]
$Ti_2N(OH)_2$ (OH_edge_O)	naked	-944.038			-931.626	
	vac-25%[#]	-937.804[*]			-931.626	
	vac-N_2	-951.754	0.171	-17.498	-931.626	-2.460
	vac-Na	-933.137	0.020	-0.265[‡]	-931.626	-1.226
	vac-O_2	-953.083	0.156	-9.919	-931.626	-11.382
Ti_2NO_2 with edge -OH termination (O_edge_OH)	naked	-846.002			-833.285	
	vac-25%[#]	-839.943[*]			-833.285	
	vac-N_2	-854.025	0.211	-17.498	-833.285	-3.031
	vac-Na	-837.271	0.029	-0.265[‡]	-833.285	-3.692
	vac-O_2	-854.575	0.180	-9.919	-833.285	-11.191
Ti_2NO_2 (O_edge_O)	naked	-832.194			-821.707	
	vac-25%[#]	-826.961[*]			-821.707	
	vac-N_2	-840.187	0.225	-17.498	-821.707	-0.757
	vac-Na	-823.200	0.032	-0.265[‡]	-821.707	-1.196
	vac-O_2	-840.416	0.159	-9.919	-821.707	-8.631

† Twice adsorbate free energy and half adsorption free energy due 2 vacancy sites for $Ti_2N(OH)O$ surface, the Nitrogen vacancy site ratio is 50%.

‡ The adsorbate free energy for single Na atoms, only includes electronic energy $E_{0.}$

25% vacancy concentration (vac-25%) E_{vac_25} describes the system where a MNene sheet with 50% vacancies is stacked with a MNene sheet containing 0% vacancies. The vacancy energy of E_{vac_50} is computed based on a system containing only the 50% vacancies in the MNene sheet. The first vacancy formation energy is calculated to create 25% vacancy concentration as follows: $E_{f\_vac\_1st} = E_{vac\_25} - E_{naked} + G_{N2}/2 = E_{vac\_25} - E_{naked} + -17.498/2$ (extracted from **Table S1**) and the Second vacancy formation energy is to create 50% vacancy concentration from 25% vacancy concentration as follows $E_{f\_vac\_2nd}= E_{vac\_50} - E_{vac\_25} + G_{N2}/2 = E_{vac\_50} - E_{vac\_25} -17.498/2$. For $Ti_2N(OH)O$, $Ti_2N(OH)_2$, Ti_2NO_2 with edge -OH termination, as well as Ti_2NO_2. Their $E_{f\_vac\_1st}$ are -2.769, -2.515, -2.690 and -3.516 eV respectively. And their $E_{f\_vac\_2nd}$ are -2.492, -2.571, -2.091 and -3.495 eV respectively. We however note that the adjacent N vacancies co-existence within a close vicinity setup was considered because of computational recourses affordability.

* For $Ti_2N(OH)_2$ and Ti_2NO_2 with edge -OH termination, as well as Ti_2NO_2, achieving 25% vacancy sites, it requires computing a supercell twice the original size. Consequently, their calculated free energies (-1875.607, -1679.885, and -1653.922 eV) were divided by two to facilitate easier comparison with other surfaces.

Table S3. The convergency test of the adsorption energy E_{ads} and Nitrogen vacancy formation energies E_{vac} under different layers number setup and different fixed layers**.** These converged tests are computed by **Eq. S20** and **Eq. S21** based on optimization structures shown in **Figure S58**. As layer increase from 5 to 9, the adsorption energy change of E_{ads} from 0.02 eV to less than 0.01 eV difference when the layer increases to 9 in fixbot4 model. On the other hand, adsorption energy E_{ads} of different fixbot2 models are slightly higher as layers of model increase with around 0.02 eV. For nitrogen vacancy formation energy E_{vac}, as layer increase, the energy is smaller is larger in E_{vac} test in fixbot4 model which mainly due to increase of mobility of layer along plane direction that stabilizes Nitrogen vacancy sites.

	**Fix layers type**	**1 × 2 × 5 slab**	**1 × 2 × 7 slab**	**1 × 2 × 9 slab**
**E_{ads} (eV)**	fixbot4	0.031	-0.003	-0.006
	fixbot2	0.031	0.012	-0.016
**E_{vac} (eV)**	fixbot4	2.059	1.865	1.795
	fixbot2	2.059	1.996	2.026

References

(1) Lai, H.-E.; Yoo, R. M.; Djire, A.; Balbuena, P. B. Investigation of the Vibrational Properties of 2D Titanium Nitride MXene Using DFT. *The Journal of Physical Chemistry C* **2024**.

(2) Rivarolo, M.; Riveros-Godoy, G.; Magistri, L.; Massardo, A. F. Clean hydrogen and ammonia synthesis in Paraguay from the Itaipu 14 GW hydroelectric plant. *ChemEngineering* **2019**, *3* (4), 87.
